# Alternative Strategies to Reduce Maternal Mortality in India: A Cost-Effectiveness Analysis

**DOI:** 10.1371/journal.pmed.1000264

**Published:** 2010-04-20

**Authors:** Sue J. Goldie, Steve Sweet, Natalie Carvalho, Uma Chandra Mouli Natchu, Delphine Hu

**Affiliations:** 1 Center for Health Decision Science, Harvard School of Public Health, Boston, Massachusetts, United States of America; 2 Department of Nutrition, Harvard School of Public Health, Boston, Massachusetts, United States of America; 3 Department of Health Policy and Management, Harvard School of Public Health, Boston, Massachusetts, United States of America; 4 Pediatric Biology Centre, Translational Health Science and Technology Institute, Delhi, India; Aga Khan University, Pakistan

## Abstract

**Background:**

Approximately one-quarter of all pregnancy- and delivery-related maternal deaths worldwide occur in India. Taking into account the costs, feasibility, and operational complexity of alternative interventions, we estimate the clinical and population-level benefits associated with strategies to improve the safety of pregnancy and childbirth in India.

**Methods and Findings:**

Country- and region-specific data were synthesized using a computer-based model that simulates the natural history of pregnancy (both planned and unintended) and pregnancy- and childbirth-associated complications in individual women; and considers delivery location, attendant, and facility level. Model outcomes included clinical events, population measures, costs, and cost-effectiveness ratios. Separate models were adapted to urban and rural India using survey-based data (e.g., unmet need for birth spacing/limiting, facility births, skilled birth attendants). Model validation compared projected maternal indicators with empiric data. Strategies consisted of improving coverage of effective interventions that could be provided individually or packaged as integrated services, could reduce the incidence of a complication or its case fatality rate, and could include improved logistics such as reliable transport to an appropriate referral facility as well as recognition of referral need and quality of care. Increasing family planning was the most effective individual intervention to reduce pregnancy-related mortality. If over the next 5 y the unmet need for spacing and limiting births was met, more than 150,000 maternal deaths would be prevented; more than US$1 billion saved; and at least one of every two abortion-related deaths averted. Still, reductions in maternal mortality reached a threshold (∼23%–35%) without including strategies that ensured reliable access to intrapartum and emergency obstetrical care (EmOC). An integrated and stepwise approach was identified that would ultimately prevent four of five maternal deaths; this approach coupled stepwise improvements in family planning and safe abortion with consecutively implemented strategies that incrementally increased skilled attendants, improved antenatal/postpartum care, shifted births away from home, and improved recognition of referral need, transport, and availability/quality of EmOC. The strategies in this approach ranged from being cost-saving to having incremental cost-effectiveness ratios less than US$500 per year of life saved (YLS), well below India's per capita gross domestic product (GDP), a common benchmark for cost-effectiveness.

**Conclusions:**

Early intensive efforts to improve family planning and control of fertility choices and to provide safe abortion, accompanied by a paced systematic and stepwise effort to scale up capacity for integrated maternal health services over several years, is as cost-effective as childhood immunization or treatment of malaria, tuberculosis, or HIV. In just 5 y, more than 150,000 maternal deaths would be averted through increasing contraception rates to meet women's needs for spacing and limiting births; nearly US$1.5 billion would be saved by coupling safe abortion to aggressive family planning efforts; and with stepwise investments to improve access to pregnancy-related health services and to high-quality facility-based intrapartum care, more than 75% of maternal deaths could be prevented. If accomplished over the next decade, the lives of more than one million women would be saved.

*Please see later in the article for the Editors' Summary*

## Abstract 2

A cost-effectiveness study by Sue Goldie and colleagues finds that better family planning, provision of safe abortion, and improved intrapartum and emergency obstetrical care could reduce maternal mortality in India by 75% in 5 years.

## Editors' Summary

**Background:**

Every year, more than half a million women—most of them living in developing countries—die from pregnancy- or childbirth-related complications. About a quarter of these “maternal” deaths occur in India. In 2005, a woman's lifetime risk of maternal death in India was 1 in 70; in the UK, it was only one in 8,200. Similarly, the maternal mortality ratio (MMR; number of maternal deaths per 100,000 live births) in India was 450, whereas in the UK it was eight. Faced with the enormous maternal death toll in India and other developing countries, in September 2000, the United Nations pledged, as its fifth Millennium Development Goal (MDG 5), that the global MMR would be reduced to a quarter of its 1990 level by 2015. Currently, it seems unlikely that this target will be met. Between 1990 and 2005, global maternal deaths decreased by only 1% per annum instead of the 5% needed to reach MDG 5; in India, the decrease in maternal deaths between 1990 and 2005 was about 1.8% per annum.

**Why Was This Study Done?:**

Most maternal deaths in developing countries are caused by severe bleeding after childbirth, infections soon after delivery, blood pressure disorders during pregnancy, and obstructed (difficult) labors. Consequently, experts agree that universal access to high-quality routine care during labor (“obstetric” care) and to emergency obstetrical care is needed to reduce maternal deaths. However, there is less agreement about how to adapt these “ideal recommendations” to specific situations. In developing countries with weak health systems and predominantly rural populations, it is unlikely that all women will have access to emergency obstetric care in the near future—so would beginning with improved access to family planning and to safe abortions (unsafe abortion is another major cause of maternal death) be a more achievable, more cost-effective way of reducing maternal deaths? How would family planning and safe abortion be coupled efficiently and cost-effectively with improved access to intrapartum care? In this study, the researchers investigate these questions by estimating the health and economic outcomes of various strategies to reduce maternal mortality in India.

**What Did the Researchers Do and Find?:**

The researchers used a computer-based model that simulates women through pregnancy and childbirth to estimate the effect of different strategies (for example, increased family planning or increased access to obstetric care) on clinical outcomes (pregnancies, live births, or deaths), costs, and cost-effectiveness (the cost of saving one year of life) in India. Increased family planning was the most effective single intervention for the reduction of pregnancy-related mortality. If the current unmet need for family planning in India could be fulfilled over the next 5 years, more than 150,000 maternal deaths would be prevented, more than US$1 billion saved, and at least half of abortion-related deaths averted. However, increased family planning alone would reduce maternal deaths by 35% at most, so the researchers also used their model to test the effect of combinations of strategies on maternal death. They found that an integrated and stepwise approach (increased family planning and safe abortion combined with consecutively increased skilled birth attendants, improved care before and after birth, reduced home births, and improved emergency obstetric care) could eventually prevent nearly 80% of maternal deaths. All the steps in this strategy either saved money or involved an additional cost per year of life saved of less than US$500; given one suggested threshold for cost-effectiveness in India of the per capita GDP (US$1,068) per year of life saved, these strategies would be considered very cost-effective.

**What Do These Findings Mean?:**

The accuracy of these findings depends on the assumptions used to build the model and the quality of the data fed into it. Nevertheless, these findings suggest that early intensive efforts to improve family planning and to provide safe abortion accompanied by a systematic, stepwise effort to improve integrated maternal health services could reduce maternal deaths in India by more than 75% in less than a decade. Furthermore, such a strategy would be cost-effective. Indeed, note the researchers, the cost savings from an initial focus on family planning and safe abortion provision would partly offset the resources needed to assure that every woman had access to high quality routine and emergency obstetric care. Thus, overall, these findings suggest that MDG 5 may be within reach in India, a conclusion that should help to mobilize political support for this worthy goal.

**Additional Information:**

Please access these Web sites via the online version of this summary at http://dx.doi.org/10.1371/journal.pmed.1000264.

- UNICEF (the United Nations Children's Fund) provides information on maternal mortality, including the WHO/UNICEF/UNFPA/The World Bank 2005 country estimates of maternal mortality
- The World Health Organization also provides information on maternal health and about MDG 5 (in several languages)
- The United Nations Millennium Development Goals Web site provides detailed information about the Millennium Declaration, the MDGs, their targets and their indicators, and about MDG 5.
- The Millennium Development Goals Report 2009 and its progress chart provide an up-to-date assessment of progress toward all the MDGs
- Computer simulation modeling as applied to health is further discussed at the Center for Health Decision Science at Harvard University

## Introduction

Approximately one-quarter of all pregnancy- and delivery-related maternal deaths worldwide occur in India, which has the highest burden of maternal mortality for any single country [1],[2]. Although the inclusion of maternal mortality reduction in the United Nations' Millennium Development Goals (MDGs) reflects the importance of improving maternal health as a key mechanism in reducing poverty and promoting social and economic growth, global progress has been suboptimal [2]–[4]. Several factors may be changing the landscape for maternal health in India in particular [5]. These factors include more information on maternal mortality measures [6],[7], an increasing number of studies evaluating interventions [8], renewed determination on the part of the maternal health and public health communities [9], and, most importantly, the emergence of maternal mortality reduction as a clear priority on the Indian national political agenda [5],[10]–[12].

Despite consensus on the need for universal access to high-quality intrapartum and emergency obstetrical care (EmOC), uncertainties remain about how to adapt “ideal recommendations” to specific situations [8],[13]. The need for an adequate supply of skilled providers, functional referral and transport, and well-equipped facilities for EmOC will prove a formidable barrier in the near-term for countries with weak health systems, as well as for states with inadequate health delivery infrastructure and for communities in predominantly rural areas [13]. This challenge will be particularly relevant for India, with its largely rural population, and striking disparities between states. For example, while Kerala reports a maternal mortality ratio (MMR) of fewer than 100 maternal deaths per 100,000 live births, rural Uttar Pradesh and Rajasthan report MMRs of more than 400 [14],[15].

To most effectively leverage India's national commitment to reducing maternal mortality, identifying evidence-based strategies that consider the local context is imperative. Our analysis is motivated by questions that include: What are the fundamental drivers of the effectiveness, cost-effectiveness, and affordability of a package of interventions to reduce maternal mortality? Because adequate facilities, health infrastructure, and skilled human resources will not be readily available in all settings, can we provide interim guidance to policy makers? While no single empirical study can provide clear answers to these questions, a modeling approach within a decision-analytic framework can extend empiric information by extrapolating outcomes beyond the time horizon of a single study, can facilitate synthesis of multiple data sources in an internally consistent and epidemiologically plausible way [16], and may be adapted to specific settings so that existing infrastructure, resources, and political realities can be considered.

Previous model-based studies have provided important insights into the potential high public health value of reducing maternal deaths, however, many of these have not considered the full range of interventions to reduce maternal mortality, such as family planning, safe abortion, and intrapartum care [7],[17],[18]. Some have only focused on single interventions [19]–[21], others have not included costs [22], and recent analyses that did assess multiple strategies did not explicitly model critical barriers to life-saving referral, such as recognition of referral need and accessible transport [23],[24]. Taking into account the costs, feasibility, and operational complexity of alternative interventions, we extend this body of work to estimate the clinical and population-level benefits associated with a comprehensive set of strategies to improve the safety of pregnancy and childbirth in India.

## Methods

### Analytic Overview

The best available data were synthesized using a computer-based model that simulates the natural history of pregnancy and relevant comorbidities, aggregates individual outcomes to the population level, and reflects setting-specific epidemiology. Separate models were adapted to urban and rural India using data on antenatal care, family planning, facility births, and skilled birth attendants (SBAs), and information about access to transport, referral facilities, and quality of care. Model outcomes include clinical events (e.g., pregnancies, live births, maternal complications), measures of maternal mortality (e.g., MMR, proportionate mortality ratio [i.e., proportion of deaths that are pregnancy-related among women aged 15–45 y], and lifetime risk of maternal death), population outcomes (e.g., life expectancy), and economic costs.

We evaluated alternative approaches to reducing maternal mortality in settings in India that differ according to underlying maternal risk, health, and socioeconomic status. Interventions can be provided individually or packaged into integrated services. Following standard recommendations for economic evaluation [25], strategies are first ranked in terms of increasing costs and benefits; those that are less effective and more costly than an alternative strategy are considered inefficient, and those that cost less than the status quo are considered “cost saving.” For all other strategies, we calculate an incremental cost-effectiveness ratio, defined as the additional cost of a specific strategy divided by its additional clinical benefit, compared with the next least expensive strategy. We considered interventions with cost-effectiveness ratios of less than the per capita gross domestic product (GDP) (US$1,068) to be very cost-effective as suggested by the Commission on Macroeconomics and Health. Sensitivity analyses are conducted to assess the impact of parameter uncertainty.

### The Model

**Figure 1:**
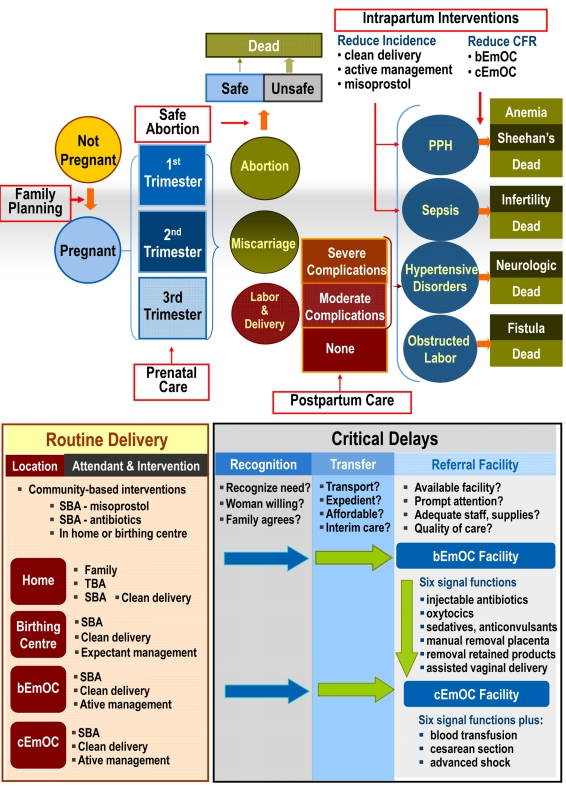
Schematic of the model. Upper panel: Model simulates the natural history of pregnancy (both planned and unintended) and pregnancy- and childbirth-associated complications. Case fatality rates for complications depend on severity and comorbidity. General intervention categories (open red boxes) include family planning for spacing or limiting births, antenatal or prenatal care (and treatment of anemia), safe abortion, intrapartum care (e.g., active management of labor), basic and comprehensive EmOC, and postpartum care. Interventions can reduce the incidence or severity of a complication or can reduce the case fatality rate through appropriate treatment. Lower panel: Model reflects the intervention pathway during labor and delivery, including location (home, birthing or health center, bEmOC, cEmOC), attendant (family member, traditional birth attendant [TBA], or SBA), and three potential barriers to effective treatment in the event of a complication, including recognition of referral need, transfer (e.g., transport), and timely quality care in an appropriate EmOC facility. Management of labor and delivery depends on attendant (e.g., SBA, clean delivery) and site (e.g., expectant management in birthing center, active management in EmOC facility), as does access to specific levels of treatment (e.g., blood transfusion only available in cEmOC).

The computer-based Global Maternal Health Policy Model simulates the natural history of pregnancy (both planned and unintended) and pregnancy- and childbirth-associated complications (Figure 1). This model defines health states to reflect important characteristics that affect prognosis, quality of life, and resource use. The time horizon incorporates a woman's lifetime and is divided into equal time increments during which women transition from one health state to another. Nonpregnant girls enter the model and in each time period may become pregnant depending on age, use of contraception, and clinical history (Figure 1, upper panel). Once pregnant, women have a chance of spontaneous abortion (i.e., miscarriage), induced abortion, or continued pregnancy. A proportion of induced abortions will be unsafe (i.e., surgical or medical abortion conducted by untrained personnel). Labor and delivery may be associated with a direct complication of pregnancy (e.g., hypertensive disorders of pregnancy, obstructed labor, hemorrhage, sepsis). Case fatality rates are conditional on the type and severity of complication (e.g., moderate sepsis requiring antibiotics versus severe hemorrhage requiring blood transfusion) and underlying comorbidity (e.g., anemia). Nonfatal complications include neurological sequelae, rectovaginal fistula, severe anemia, and infertility (Figure 1, upper panel). In addition to death from maternal complications, women face an annual risk of death from age-specific all-cause mortality.

Strategies in the model to reduce maternal mortality consist of improving coverage of effective interventions, which may be provided individually or packaged as integrated services. In addition to family planning, antenatal care (i.e., prenatal care) and treatment of anemia, safe abortion, and postpartum care, the model includes both intrapartum interventions that reduce the incidence of a complication (e.g., misoprostol for postpartum hemorrhage [PPH], clean delivery for sepsis), as well as those that reduce the case fatality rate through appropriate management in a referral facility (Figure 1, upper panel).

The effectiveness of interventions to either reduce the incidence of complications or to reduce case fatality rates associated with complications depends, in part, on access to specific services (e.g., trained SBA) and to specific levels of facilities (e.g., comprehensive EmOC [cEmOC] with capacity for blood transfusion). Accordingly, the ultimate impact of interventions depends on several setting-specific factors. These include delivery site, presence of birth attendant, quality and type of referral facility, as well as successful referral when necessary. The model therefore explicitly considers the location of delivery, type of assistance, access to basic or comprehensive obstetrical care, and the ability to overcome a series of barriers around the timing of delivery (e.g., recognition of referral need, reliable transport, timely treatment at an appropriate referral facility); these factors collectively determine the health services a woman can access and the specific interventions that would be included (Figure 1, lower panel).

Delivery setting is differentiated by provider (e.g., family member, traditional birth attendant [TBA], or SBA) and by site (e.g., home versus facility). Facility levels are categorized as (1) birthing centers or health centers, which cannot provide all services necessary to qualify as a basic emergency obstetrical care (bEmOC) facility, but are staffed with SBA who provide expectant management of labor and more reliable referral when necessary than with delivery at home; (2) facilities with bEmOC capacity (e.g., first referral units); and (3) facilities with cEmOC capacity (e.g., district hospitals) [26],[27]. Facilities capable of bEmOC are assumed to be capable of administering injectable antibiotics, oxytocics, and sedatives or anticonvulsants, and performing manual removal of placenta, removal of retained products, and assisted vaginal delivery. Facilities capable of cEmOC also are able to provide blood transfusion, cesarean section, and management of advanced shock.

**Figure 2:**
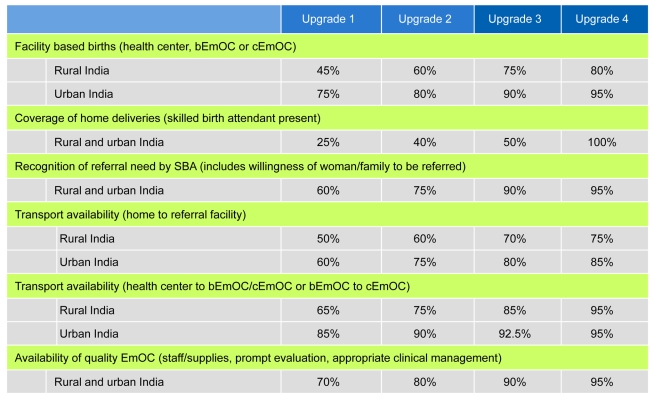
Stepwise improvements in scaling up maternal services. Four strategies that scale up access to critical maternal health services in consecutive phases are designated as upgrade 1, upgrade 2, upgrade 3, and upgrade 4. Shown are the percent increases in facility-based delivery, SBAs, recognition of referral need (by SBA at birthing/health center), transport (to appropriate referral facility), and availability/quality of EmOC (including adequate staff/supplies, appropriate clinical treatment, immediate attention), for rural and urban India. Shifts from home births assume a 70% shift to health centers/birthing centers and a 30% shift to EmOC; for routine births in EmOC, we assume 90% bEmOC and 10% cEmOC. Alternatives evaluated in sensitivity analysis (Results and Text S1).

This model also allows us to evaluate phased approaches that involve scaling up access to services over time; the stepwise investments in infrastructure required to assure high-quality intrapartum care are designated as “upgrades.” In addition to reducing unmet need for family planning and unsafe abortion, four consecutively implemented strategies increased skilled attendants, improved antenatal/postpartum care, incrementally shifted births away from home, and improved the availability and quality of EmOC. For women delivering at home or in birthing centers, these “upgrades” also improved recognition of referral need, access to transport, and expedient referral to an appropriate facility (Figure 2).

All models were built using TreeAge Pro 2008 (TreeAge Software Inc.) and analyzed using IBM/Lenovo Dual-Core VT Pro Desktop computers running Microsoft Windows XP, using Microsoft Excel 2007 and Visual Basic for Applications 6.5 (Microsoft Corp.). We used Monte Carlo simulation to generate the number of per woman events such as pregnancies, live births, facility-based births, and maternal complications.

### Data

Selected parameters and assumptions used in the model are provided in Tables 1 and 2 [7],[14],[15],[17],[22],[23],[28]–[73]. Additional details are provided in Text S1. Initial estimates of incidence and case fatality rates associated with pregnancy-related complications were obtained from published data, and a plausible range for sensitivity analysis was based on review of the literature. Case fatality rates were adjusted based on complication severity (e.g., life-threatening complications requiring cEmOC) and underlying severity of anemia (Table 1; Text S1). The effectiveness of interventions to reduce the incidence of complications (e.g., active management of labor) was estimated from published studies using methods detailed in Text S1. The effectiveness of interventions to reduce case fatality rates was from published studies and assumed treatment in an appropriate facility; a wide plausible range was used for sensitivity analyses (Table 1; Text S1). Data on facility births, SBAs, family planning for spacing or limiting births, and antenatal care were from country-specific surveys [28].

**Table 1:** Selected model parameters: Incidence and mortality of pregnancy and delivery-related complications, and impact of interventions.

Parameter	Hemorrhage	Obstructed Labor	Hypertensive Disorders	Sepsis	Unsafe Abortion
**Incidence and mortality**					
Probability of event [15],[29],[31]–[44]	0.114	0.047	0.035	0.050	0.128^a^
Range	0.051–0.228	0.030–0.074	0.025–0.050	0.043–0.060	0.050–0.250
Probability of morbidity [28],[30],[45]–[48] b	0.008	0.022	0.001	0.400	0.120
Range	0.006–0.010	0.018–0.026	0.001–0.001	0.320–0.480	0.096–0.144
CFR [7],[49]–[51]	0.010	0.007	0.017	0.013	0.003
Adjusted CFR^c^	0.023	0.019	0.021	0.028	0.009
Range	0.007–0.030	0.005–0.025	0.012–0.027	0.009–0.036	0.002–0.012
Attributable mortality [29] d	46.2% (9%–73%)	14.1% (3%–52%)	13.7% (0%–18%)	17.4% (0%–20%)	8.6% (0%–20%)
Model-projected attributable mortality	40.6%	16.8%	12.3%	20.4%	9.8%
**Impact of interventions**					
Decreased incidence [34],[37] e	50%,75%^e^	—	—	25%, 50%^f^	—
Range^f^	25%–91%	—	25%–50%	0%–60%	0%–100%
Decreased CFR [7],[17],[50],[52]–[64] g	75%	95%	59%	90%	98%^a^
Range^f^	60%–90%	76%–100%	45%–95%	63%–93%	50%–100%

See [7],[14],[15],[17],[22],[23],[28]–[64],[69]–[73].

a Incidence of elective abortion is 0.170, and 75% are assumed to be unsafe in the base case [15],[29],[39]–[44] . Case fatality rate (CFR) of safe abortion is 0.000006, representing a 98% reduction in mortality [50],[62]–[64]. For more details on abortion-related assumptions, see Text S1. Incidence of miscarriage (not shown) is 0.150 [69],[70].

b Specific examples of nonfatal complications include Sheehan's Syndrome following maternal hemorrhage, fistula resulting from obstructed labor, neurological sequelae from eclampsia, pelvic inflammatory disease (PID). Not shown but included are the risk of infertility from PID (0.086) and the risk of severe anemia following maternal hemorrhage (0.09) [23],[45],[46].

c CFRs were adjusted based on complication severity (e.g., life threatening complications requiring cEmOC) and underlying severity of anemia [71]. See Text S1.

d Estimates for distribution of causes of maternal mortality for India are from India overall estimates from Khan et al. [29], based on the entire Asia region, as well as other data to establish a range for sensitivity analysis [14],[15]. Cause-specific proportions sum to 66%, reflecting approximately 33% indirect causes, although this varies from 15% to 35% in different studies. Estimates shown reflect adjustment of data from Khan et al. [29] such that a distribution is shown for the 66% of direct causes, to compare to model output. Further, anemia was reported to be responsible for 15% of deaths and was assumed to exert mortality impact on direct causes through severity of PPH, sepsis, and unsafe abortion.

e Incidence of sepsis reduced by 50% with SBA and clean delivery in birthing center, bEmOC, and cEmOC; and reduced by 25% with SBA and clean delivery at home [37]. Incidence of maternal hemorrhage reduced by 50%–75% depending on expectant versus active management of labor; we assume for the status quo, all cEmOC facilities provide active management, 50% of bEmOC facilities provide active management, and birthing centers/health centers provide expectant management only [34]. Exploratory analyses that estimate the impact of community-based provision of oral misoprostol in birthing centers and at home assume a 25% to 50% reduction in PPH [22],[72]. For each baseline estimate, sensitivity analysis was conducted across a plausible range based on literature review; references are documented in the Text S1.

f For each baseline estimate, sensitivity analysis was conducted across a plausible range based on literature review; references and assumptions are documented in the Text S1.

g Estimates shown represent average reduction in case fatality rate provided complications necessitating surgery (e.g., cesarean section), blood transfusion, intensive hemodynamic support are treated in cEmOC. Obstructed labor is managed using assisted vaginal delivery with forceps or vacuum and, if necessary, cesarean section; severe pre-eclampsia and eclampsia treated with intravenous hydralazine and magnesium sulfate, in addition to induction of labor or emergency cesarean section when required; sepsis treated with ampicillin, gentamycin, and metronidazole or equivalent regimen followed by an 8-d course of intramuscular gentamycin and oral metronidazole (see Text S1 for details) [7],[73].

**Table 2:** Selected model parameters and assumptions: Coverage of interventions and maternal health indicators by setting.

Parameter	India	India, Urban	India, Rural	Rajasthan	Uttar Pradesh, Rural
**Coverage of contraception (%) ** **[28]**					
Family planning (any method)	56.3	64.0	53.0	47.2	39.7
Modern methods	48.5	55.8	45.3	44.4	25.2
Pill	6.4	7.0	6.2	4.5	5.2
IUD	3.7	6.1	2.4	3.6	3.2
TOL	76.9	67.7	81.9	77.0	66.7
Condom	10.9	17.9	7.3	12.8	24.6
Unmet need	13.2	10.0	14.6	14.6	23.8
**Coverage of prenatal care (%) ** **[28]**					
Prenatal care	50.7	73.8	42.8	41.2	22.6
Treatment for anemia^a^	22.3	34.5	18.1	13.1	6.7
**Delivery location (%) ** [**28**]					
Total skilled delivery	48.3	75.2	39.1	41.0	23.8
Facility delivery^b^	40.7	69.4	31.1	29.6	17.5
Home delivery with SBA^b^	12.8	19.0	11.6	16.2	7.6
**Assumptions for available transport/interim care to appropriate facility (%) ** **[65],[66]**					
From home to EmOC	30.4	44.4	24.4	24.4	18.1
Range	20–40	35–55	15–35	20–40	15–30
From HC or BC to EmOC	54.8	68.8	48.8	48.8	36.1
Range	40–65	60–80	40–60	30–55	25–45
From bEmOC to cEmOC	67.0	81.0	61.0	61.0	45.1
Range	55–80	70–90	50–70	50–70	35–55
**Assumptions for available facility, staff/supplies, quality of care (%) ** **[67],[68]**					
EmOC	50.0	67.5	42.5	42.5	31.5
Range	40–60	55–80	30–55	30–55	20–40
**Maternal health indicators**					
TFR [28]	2.68	2.07	2.98	3.21	4.13
Model-projected TFR^c^	2.70	2.07	2.97	3.24	4.13
Model-projected MMR^c^	440	407	520	524	633

See [14],[15],[28],[65]–[68],[71].

a Case fatality rates (CFRs) were adjusted based on complication severity (e.g., life threatening complications requiring cEmOC) and underlying severity of anemia [71]. See Text S1.

b Routine deliveries in EmOC facilities assume that 90% would be in bEmOC and 10% in cEmOC. Alternative assumptions explored in sensitivity analysis (Text S1). We calculated the percentage of births with skilled attendance at home by subtracting the percentage delivered in facilities (which we assume are with skilled attendance) from the total of births with skilled attendance: (total skilled delivery−facility based births)/home births; for rural India: (0.391−0.311)/(1−0.311) = 0.116 or 11.6%.

c Using the empirically calibrated India model, we parameterized the state-level models for Rajasthan and Uttar Pradesh and adjusted for the TFR as reported in NFHS 3 [28]. To provide comparison, reported MMRs for Uttar Pradesh include an estimate of 707 from Mills [14], and prior data from SRS including the SRS 2001–2003 estimate of 517 (confidence interval [CI] 461–573), SRS 1999–2001 estimate 539 (481–596), and the SRS 1997–98 (606, CI 544–668) [15]. For Rajasthan, we used the 2001–2003, special survey of deaths using RHIME, which reported 445 (371–519), and SRS 1999–2001, which reported 501 (423–580) [15].

BC, birthing center; HC, health center; IUD, intrauterine device; TOL, female sterilization.

For women delivering at home or in a birthing center, the probability of successful referral depended on overcoming three potential barriers [74]: (1) delay in recognizing referral need; (2) delay in transfer to referral facility (means of transport and interim care en route); (3) delay in receiving appropriate care at the appropriate EmOC facility such as inadequate staffing and supplies, inexpedient attention (e.g., delay to collect fees), and/or non-evidence-based or substandard care. Assumptions about the latter two delays were based on survey data (e.g., National Family Health Survey [NFHS-3] [28],[75], District Level Household Survey [DLHS] [76], and Facility Survey [26]), state-level facility surveys [77],[78], government reports [27], and published studies (Text S1) [14],[15],[79]–[83].

Model performance was assessed by comparing the distribution of direct causes of maternal mortality, life expectancy, proportionate mortality ratio, MMR, and total fertility rate (TFR) to empiric data [2],[14],[15],[28],[29],[32],[38],[84]–[87] in rural and urban India. Model validation was assessed by using state-specific data from Rajasthan and Uttar Pradesh as model inputs, and comparing model-projected indicators of maternal mortality with survey-reported outcomes [14],[15]. In addition, a secondary analysis assessing all strategies evaluated in the base case was conducted in Uttar Pradesh. Details of this process are included in Text S1.

### Costs

Selected costs used in the model are provided in Table 3 [18],[25],[80],[88]–[94]. Details are provided in Text S1. With the exception of facility costs, salaries, and transport costs, resource requirements to deliver interventions and the costs of maternal complications were estimated from the United Nations Population Fund's (UNFPA) Reproductive Health Costing Tools Model (RHCTM) [88]. The RHCTM uses an ingredients approach to estimate direct costs (including drugs, supplies, and personnel requirements) of 45 reproductive health interventions, as well as investments required for scale-up. We obtained personnel costs (salaries) and facility costs from public access country-specific databases [25],[89], and drugs and supply costs from the UNICEF Supply Catalogue and Management Sciences for Health (MSH) International Drug Price Indicator Guide [90],[91]. We leveraged public access sources and published studies to inform assumptions about the financial requirements for improving transport and scaling up facility- and human-resource capacity [7],[30],[80],[92],[93]. We assessed the face validity of model input values and established a plausible range for sensitivity analysis by comparing estimates to those in published studies (Text S1). All costs were converted to 2006 US$.

**Table 3:** Selected model input costs.

Cost Components	Base Case	Range^a^
**Family planning ** **[25],[88]**		
Oral contraceptives	10.64	6.03–15.96
Injectable contraceptives	10.20	4.92–15.30
Condoms	8.40	3.79–12.60
Intrauterine device	9.17	2.58–13.76
Female sterilization	18.98	9.49–28.47
Male sterilization	12.67	6.34–19.01
**Antenatal care** b **[25],[88]**		
Four visits	17.82	8.54–25.61
**Abortion** c **[25],[88],[94]**		
Incomplete abortion	8.90	4.45–17.80
Elective abortion	21.87	10.94–43.74
Postabortion complications	43.40	21.70–86.80
**Delivery** d **[25],[88]**		
Home (TBA, SBA)	4.52, 6.44	0–9.66
Facility (birthing center, bEmOC, cEmOC)	14.46, 24.58, 32.54	7.23–48.81
**Community-based interventions** e		
Misoprostol (home, birthing center)	0.99	0.75–2.00
SBA training	3.40	0.62–5.00
**Transportation costs** f		
Home to facility	3.62–8.13	1.81–12.20
Birthing/health center/bEmOC to referral facility	4.88–7.14	2.44–10.71
**Management of complications** g **[25],[88]**		
Obstructed labor	70.16	12.76–139.38
Maternal hemorrhage	67.99	18.40–212.51
Puerperal sepsis	47.92	23.15–111.02
Severe pre-eclampsia/eclampsia	65.85	33.50–153.62
**Postpartum care** h **[25],[88]**		
One visit	4.99	1.04–7.49

See [18],[25],[80],[88]–[94].

Estimates of costs under current standard of care (2006 US$). Estimates for the base case were country-specific and from UNFPA's Reproductive Health Costing Tools Model (RHCTM) [88] and WHO CHOICE/public databases [25],[89]–[91], unless otherwise specified. Costing details and methods for converting costs to 2006 US$ are provided in the Text S1.

a Ranges for sensitivity analyses established on the basis of assumptions and other published literature documented in the Text S1.

b Antenatal care includes tetanus vaccination, syphilis, gonorrhea, chlamydia screening (and treatment), urinalysis, blood tests, treatment for anemia, counseling (e.g., family planning, spacing, intrapartum care).

c Postabortion complications assumed to require manual vacuum aspiration, treatment of sepsis in 25%, surgical repair in 25% [92].

d Total costs reflect skill level of attendant, level of facility, and drugs and supplies. For example, delivery at birthing center (US$14.46) includes personnel (US$6.44), facility (US$4.52), and drugs and supplies (US$3.50). Other assumptions documented in the Text S1.

e Community-based interventions evaluated in sensitivity analysis included SBA-administered misoprostol to reduce incidence of PPH in deliveries at home and in birthing centers. Costs for misoprostol (US$0.99) and training (upper bound, US$3.40) based on assumptions presented in Sutherland and Bishai [18]; these costs represent the incremental costs above routine SBA delivery.

f Transport costs include those incurred from home to a referral facility (bEmoc or cEmOC), and those incurred between facilities when necessary (e.g., bEmOC to cEmOC). Assumptions based on literature [80],[93],[94] and public access data described in the Text S1.

g Estimates shown represent average total costs using case-specific unit costs weighted by severity. Complications requiring surgery (e.g., cesarean section), blood transfusion, intensive hemodynamic support assumed to require cEmOC. Details of unit cost assumptions for facility-specific treatment documented in Text S1.

h Postpartum care includes examination, iron/folate supplementation, and counseling.

TBA, traditional birth attendant.

## Results

### Model Validation

Model-estimated life expectancy for a 15-y-old female was 55 y compared to the World Health Organization's (WHO) estimate of 55.1 y [85]. The distribution of maternal deaths by cause closely approximated published regional estimates (Table 1) [29]. Model-generated estimates of TFR and MMR for India, stratified for rural and urban status, closely approximated survey-reported values [1],[2],[28],[38], as did the state-specific models for Rajasthan and Uttar Pradesh (Table 2) [14],[15]. Model predicted deaths for 2005, taking into account direct and indirect causes of maternal mortality, were 117,657, compared to 117,000 estimated by UNICEF, WHO, and UNFPA [86],[87].

### Enhanced Family Planning and Safe Abortion

Increased family planning to reduce the unmet need (for spacing and limiting births) by amounts ranging from 25% to 100%, reduced maternal deaths by amounts ranging from 7.0% to 28.1% in rural India and 5.8% to 23.5% in urban India (Table 4). In rural India, eliminating the unmet need for family planning decreased the TFR from 2.97 to 2.14, the proportion of deaths that are pregnancy related from 16.4% to 12.3%, and the lifetime risk of maternal death from 1 in 65 to 1 in 90. In rural India alone, the cost savings for a single birth cohort of 15-y-old girls (2010) that would be expected to accrue over their reproductive lifespans ranged from US$111.4 million to US$448.2 million. Reducing the unmet need, coupled with provision of safe abortion, provided synergistic benefits and saved additional costs (Table 4). Results were similar in urban India, although the amount of deaths averted and costs saved were smaller, reflecting both the lower initial TFR and the smaller population size (Table 4).

**Table 4:** Health and economic outcomes of family planning to reduce the unmet need for limiting and spacing births, and safe abortion, in rural and urban India.

Strategy	Lifetime Deaths per 100,000 Women	Reduction in Maternal Deaths	Proportionate Mortality Ratio	Lifetime Risk of Death Due to Maternal Complications	Model-projected Savings for a Single Birth Cohort of 15 y olds (US$)^a^	Cost Savings for a Single Year (Current Distribution of 15–45 y Olds in India) (US$)^b^
**Rural India, current conditions (TFR, 2.97)** c	1,543	—	16.4%	1 in 65	NA	—
Family planning						
Reduce unmet need 25% (56.7%)	1,435	7.0%	15.4%	1 in 70	111,357,615	60,200,655
Reduce unmet need 50% (60.3%)	1,327	14.0%	14.4%	1 in 75	223,221,615	120,611,563
Reduce unmet need 75% (64.0%)	1,218	21.1%	13.4%	1 in 82	335,439,615	181,233,496
Reduce unmet need 100% (67.6%)	1,109	28.1%	12.3%	1 in 90	448,188,615	242,067,230
Safe abortion						
Increase safe abortion 50%	1,517	1.7%	16.2%	1 in 66	48,080,115	42,078,125
Increase safe abortion 75%	1,473	4.5%	15.8%	1 in 68	130,739,115	114,289,234
Increase safe abortion 95%	1,433	7.1%	15.4%	1 in 70	214,460,115	167,790,590
Family planning and safe abortion						
Reduce unmet need (56.7%), safe abortion 75%	1,369	11.3%	14.8%	1 in 73	233,930,115	166,870,014
Reduce unmet need (60.3%), safe abortion 75%	1,265	18.0%	13.8%	1 in 79	337,386,615	219,568,526
Reduce unmet need (64.0%), safe abortion 75%	1,160	24.8%	12.8%	1 in 86	441,108,615	272,385,038
Reduce unmet need (67.6%), safe abortion 95%	1,026	33.5%	11.5%	1 in 98	580,230,615	362,579,472
**Urban India, current conditions (TFR, 2.07)** d	842	—	9.6%	1 in 119	NA	—
Family planning						
Reduce unmet need 25% (66.5%)	793	5.8%	9.1%	1 in 126	22,089,305	12,838,532
Reduce unmet need 50% (69.0%)	743	11.7%	8.6%	1 in 135	44,214,305	25,696,437
Reduce unmet need 75% (71.5%)	694	17.6%	8.1%	1 in 144	66,398,305	38,578,054
Reduce unmet need 100% (74.0%)	644	23.5%	7.5%	1 in 155	88,611,805	51,483,279
Safe abortion						
Increase safe abortion 50%	822	2.4%	9.4%	1 in 122	11,351,305	9,742,159
Increase safe abortion 75%	788	6.4%	9.1%	1 in 127	30,821,305	26,413,778
Increase safe abortion 95%	758	9.9%	8.7%	1 in 133	50,438,805	36,823,443
Family planning and safe abortion						
Reduce unmet need (66.5%), safe abortion 75%	741	11.9%	8.6%	1 in 135	51,235,305	37,766,193
Reduce unmet need (69%), safe abortion 75%	695	17.5%	8.1%	1 in 144	71,649,305	49,126,308
Reduce unmet need (71.5%), safe abortion 75%	648	23.0%	7.6%	1 in 154	92,122,305	60,499,204
Reduce unmet need (74%), safe abortion 95%	580	31.2%	6.8%	1 in 173	119,557,305	79,270,520

See [87]. Reduction in direct causes of maternal mortality, including abortion-related complications, postpartum hemorrhage, hypertensive disorders, sepsis, and obstructed labor.

a Model-projected cost savings reflect net costs averted over a woman's reproductive lifespan (ages 15–45 y) applied to the current population of 15 y olds in India stratified by rural (75%) and urban (25%) settings [87]. Future costs discounted 3% annually.

b Cost savings for a single representative year of a successfully implemented strategy were calculated using population-level data from India [87] stratified by rural (75%) and urban (25%) settings, for the current distribution of reproductive age women (ages 15–45 y).

c In rural India, model-projected TFR is 2.76, 2.56, 2.36, 2.14 with reductions in unmet need of 25%, 50%, 75%, 100%, respectively.

d In urban India, model-projected TFR is 1.94, 1.82, 1.71, 1.59 with reductions in unmet need of 25%, 50%, 75%, 100%, respectively.

**Figure 3:**
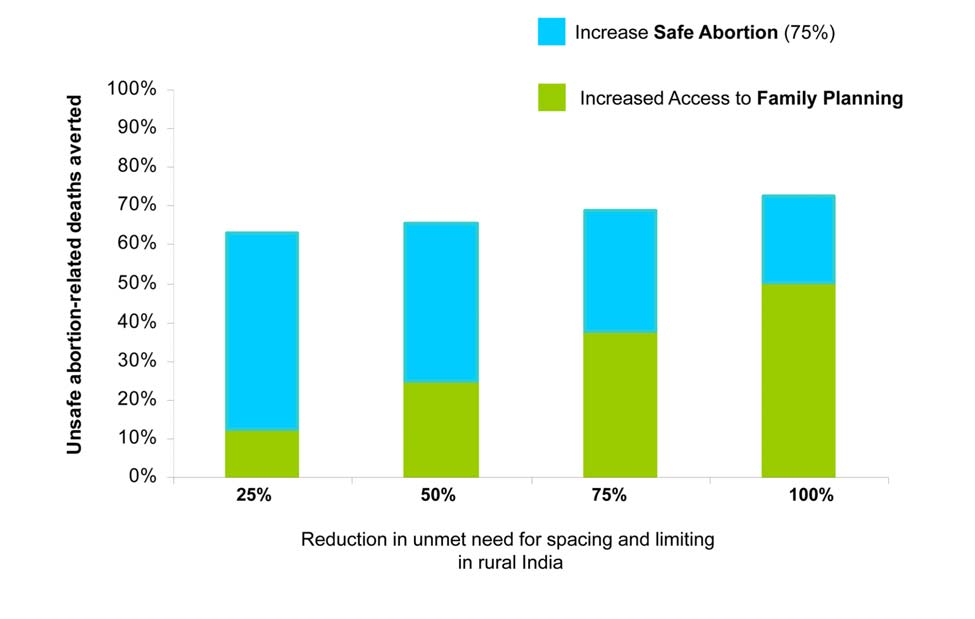
Averted deaths with family planning and safe abortion. Averted deaths attributable to unsafe abortion in rural India by addressing need for family planning (green shading) and providing 75% safe abortion (blue shading). Magnitude of additional averted abortion-related deaths with improved access to safe abortion depends on the amount of unmet need for contraception.

Increased family planning to reduce the unmet need also reduced the number of deaths attributable to unsafe abortion (Figure 3). For example, in rural India increasing contraceptive rates to 67.6% cut abortion-related deaths by more than 50%—even with no change in rates of unsafe abortion. Adding improved access to safe abortion and postabortion care for three out of four women pursuing elective termination of pregnancy prevented an additional 22% to 50% of abortion-related deaths, depending on the underlying level of unmet need; similarly, the additional cost savings ranged from 22% more, to more than double the savings expected from family planning alone.

### Interventions Packaged as Integrated Services

Our results suggest that reaching the MDG 5 goal of a 75% reduction in maternal mortality would require investments targeting the intrapartum period, in addition to family planning and safer abortion. Without these additional strategies, the model predicts a ceiling on the level of maternal mortality reduction achievable, ranging from 32% in urban India to 34% in rural India.

Table 5 shows the health and economic outcomes associated with interventions packaged as integrated services; these included phased approaches that scale up access to intrapartum services over time in rural (upper section) and urban India (lower section), coupled with incremental improvements in family planning and safe abortion. The four stepwise “upgrades” incorporated improvements in available SBAs for home births, recognition of referral need, transport, and availability/quality of EmOC, as well as shifts from home- to facility-based delivery.

**Table 5:** Health and economic outcomes of integrated packages of services: intrapartum care, family planning, and safe abortion.

Region	Facility Birth (%)	Transport-Home^a^ (%)	Transport-Facility^a^ (%)	Quality of Care^b^ (%)	Family Planning^c^ (%)	Safe Abortion (%)	Decrease in Maternal Deaths (%)	MMR (Deaths per 100,000 Live Births)	Maternal Deaths as Percent of Deaths Ages 15–45 y	Lifetime Risk of Maternal Death	Lifetime Costs^d^ (US$)	Cost-Effectiveness^d^	
												ICER (US$/YLS)	ICER (% per capita GDP)
**Rural India** e	**—**	**—**	**—**	**—**	**—**	**—**	**—**	520	16.4	1 in 65	218.38	—	**—**
Upgrade 1	45	50	65	70	56.7	50	17.3	460	14.0	1 in 78	212.41	CS	CS
Upgrade 2	60	60	75	80	60.3	60	33.7	397	11.5	1 in 98	218.51	150	14
Upgrade 3	75	70	85	90	64.0	75	53.4	302	8.3	1 in 139	226.18	160	15
Upgrade 4	80	75	95	95	67.6	95	77.1	162	4.3	1 in 282	243.46	300	28
**Urban India** f	**—**	**—**	**—**	**—**	**—**	**—**	**—**	407	9.6	1 in 119	184.00	—	**—**
Upgrade 1	75	60	85	70	66.5	50	15.8	363	8.2	1 in 141	174.91	CS	CS
Upgrade 2	80	75	90	80	69.0	60	33.3	305	6.6	1 in 178	178.30	150	14
Upgrade 3	90	80	92.5	90	71.5	75	54.1	225	4.7	1 in 259	183.44	220	21
Upgrade 4	95	85	95	95	74.0	95	78.5	113	2.3	1 in 553	194.97	350	33

Reduction in direct causes of maternal mortality, including abortion-related complications, postpartum hemorrhage, hypertensive disorders, sepsis, and obstructed labor.

a Transport encompasses the expedient availability of means of transport (e.g., vehicle, cart), fuel (if needed), driver, and interim attendant care. Facility transport represents a weighted average of transport availability from a health center or birthing center to an EmOC facility and from a bEmOC facility to a cEmOC if indicated. Accuracy of referral need recognition at home and in health center with SBA increase, on average, to 60%, 75%, 90%, and 95% (not shown) with upgrade 1, 2, 3, and 4 in both rural and urban India.

b Quality refers to the availability and quality of services at EmOC facilities, including adequate staffing and supplies, expedient attention (e.g., without delay to collect fees or requirement for family to bring supplies), and evidence-based clinical practices.

c Family planning refers to contraceptive use for limiting and spacing; shown are values representing the reduction in unmet need by 25%, 50%, 75%, and 100% with upgrade 1, 2, 3, and 4, respectively, for both rural and urban India.

d Stepwise improvements in maternal health services are assumed to occur in consecutive phases (e.g., first upgrade 1, then upgrade 2, etc.). Therefore, the incremental cost-effectiveness ratio (US$ per YLS) for each upgrade is calculated as the difference in lifetime costs relative to the difference in lifetime effects, compared with the preceding next best strategy. Cost-effectiveness ratios are also expressed as percent of the per capita GDP (US$1,068), shown in the farthest right column, as interventions with cost-effectiveness ratios of less than the per capita GDP are considered very cost-effective according to criteria proposed by the Commission on Macroeconomics and Health [98].

e Status quo (rural India): 31.1% facility births; 11.6% SBA (home births); transport from home (24.4%), primary-level health center (48.8%), bEmOC (61%); recognition of referral need at home (20%), primary-level health center (40%); availability and quality of EmOC (42.5%); 53% family planning.

f Status quo (urban India): 69.4% facility births; 19% SBA (home births); transport from home (44%), primary-level health center (69%), bEmOC (81%); recognition of referral need at home (20%), primary-level health center (40%); availability and quality of EmOC (67.5%); 64% family planning.

CS, cost saving; ICER, incremental cost-effectiveness ratio.

Compared to the status quo in rural India (upper section, Table 5), our model predicted that integrated strategies coupling family planning and safe abortion with four consecutive “upgrades” would be expected to reduce maternal deaths by 17.3% to 77.1%, the MMR to less than 200, proportionate mortality ratio by 14% to 4%, and lifetime risk of maternal mortality from one in 78 to one in 282. Compared to the status quo in urban India (Table 4, lower section), similar reductions in maternal deaths were predicted; although the number of absolute lives saved would be lower, the MMR, lifetime risk, and proportionate mortality ratio would be expected to decline to 113, one in 553, and 2.3%, respectively, with the most intensive strategy (upgrade 4).

Because the stepwise improvements in each component of the integrated package (intrapartum care, family planning, and safe abortion) were assumed to occur in consecutive phases, the incremental cost-effectiveness ratio for each “upgrade” strategy was calculated as the difference in costs relative to the difference in effects, compared with the preceding next best strategy. While the initial strategy was cost saving in both urban and rural India, incremental cost-effectiveness ratios ranged from US$150 to US$300 per YLS in rural India and from US$150 to US$350 per YLS in urban India. Cost-effectiveness ratios are also expressed as percent of the per capita GDP (US$1,068). Even the most intensive and effective strategic package was well below 50% of the per capita GDP.

In contrast to these integrated strategies, implementing only the stepwise intrapartum care upgrades—without family planning and safe abortion—was less effective and less cost-effective. The incremental cost-effectiveness ratios ranged from US$490–US$1,060 in rural India and US$200–US$990 per YLS in urban India (Text S1).

### Integrated Safe Motherhood Interventions in Rural Uttar Pradesh

**Figure 4:**
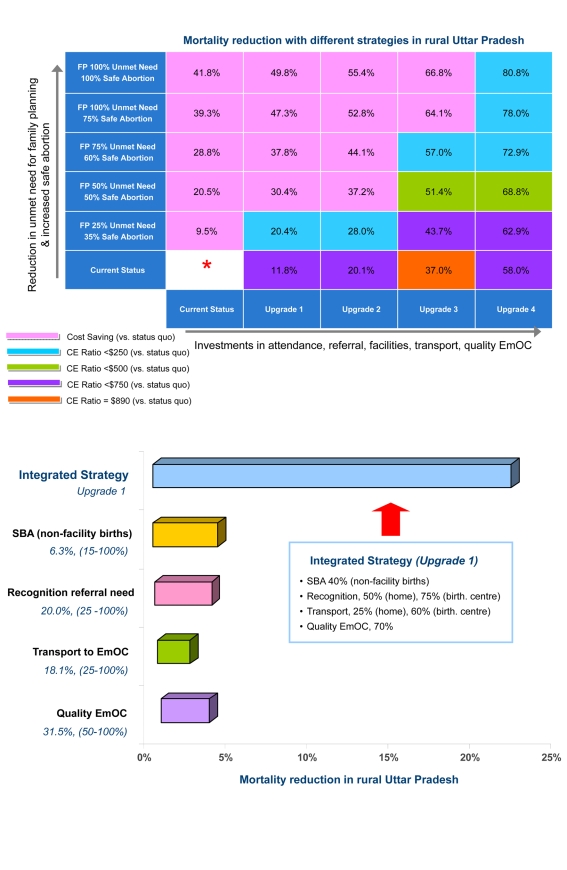
Health and economic outcomes in rural Uttar Pradesh. Upper panel. Reduction in maternal deaths and cost-effectiveness with stepwise approaches to improve maternal health in rural Uttar Pradesh. The vertical axis (from bottom to top) shows outcomes associated with increased access to family planning and safe abortion. The horizontal axis (from left to right) displays outcomes associated with investments in high-quality health-center–based intrapartum care, which involved stepwise improvements in SBAs, recognition of referral need, and antenatal/postpartum care, incrementally shifted births away from home, and improved transport, availability, and quality of EmOC. Each cell represents a unique strategy; the reduction in maternal deaths shown is relative to current conditions (far lower left corner). Shading reflects cost-effectiveness ratios, compared to status quo (pink, cost saving; blue, <US$250/YLS; green, <US$500/YLS; purple, <US$750/YLS; orange, US$890/YLS). See text for details. Lower panel. Sensitivity analysis depicting the impact of improving only one component of intrapartum care services in rural Uttar Pradesh. Base case and improvements for each component are shown in numbers below the component name (e.g., quality of EmOC is 31.5% in the base case, improvements of 50%–100% were assessed). In settings where most deliveries occur at home, linkage of services in multiple domains is a critical determinant of reduction in maternal deaths. Even large improvements in skilled birth attendants for nonfacility births (yellow bar), recognition of referral need (pink bar), transport (green bar), or quality of EmOC (purple bar) fail to reduce mortality more than 2%–5% if other interdependent components are not improved as well. In contrast, even modest improvements in all four components, as shown in upgrade 1 (blue bar), reduce mortality by 22%. Cost-effectiveness of the integrated strategy ranged from cost saving to US$170 per YLS compared with US$700 to US$4,900 per YLS for single unlinked improvements.

Figure 4 (upper panel) displays the health outcomes associated with stepwise approaches to improve maternal health in rural Uttar Pradesh. The vertical axis (from bottom to top) shows outcomes associated with increased access to family planning and safe abortion; the horizontal axis (from left to right) displays outcomes associated with investments in high-quality health-center–based intrapartum care (e.g., facility, attendance, referral, transport, EmOC). In Figure 4 (upper panel) the cell located in the far left lower corner represents current conditions in rural Uttar Pradesh. Each of the other cells represents a unique strategy; the reduction in maternal deaths expected with each strategy, relative to current conditions, is shown. For example, a strategy that reduced the unmet need by 75%, increased safe abortion to 60%, and implemented improvements to intrapartum care consistent with upgrade 3, reduced maternal deaths by 57%.

In Figure 4 (upper panel) each cell is also color-coded to reflect the cost-effectiveness profile associated with the particular strategy. Strategies that only employed family planning and safe abortion (vertical axis, from bottom to top) were generally cost saving, but reduced mortality by a maximum of 40%. Strategies that only invested in intrapartum care improvements (horizontal axis, left to right) were generally associated with the highest cost-effectiveness ratios (i.e., least attractive), reflecting the higher costs required for infrastructure improvements. An overarching strategic approach that moves along the diagonal, from the lower left corner to the upper right corner, was most effective and cost-effective; the cost savings from enhanced family planning and safe abortion offset the resources required to improve intrapartum care.

### Sensitivity Analyses

For deliveries at home and in birthing centers in rural Uttar Pradesh, removing only one “delay” in accessing EmOC had minimal impact (<5%) on lowering maternal mortality and was not cost-effective (e.g., US$700–US$4,900 per YLS) (Figure 4, lower panel). In contrast, an integrated strategy that made modest improvements in all components (e.g., SBA, referral, transport, and quality) reduced mortality by 22%. Cost-effectiveness of an integrated strategy ranged from cost saving to US$170 per YLS (Text S1).

Universal antenatal care by itself averted fewer than 2% of maternal deaths; however, if enhanced antenatal care increased the probability of either facility-based delivery or SBA-attended birth (linked with accurate referral and transport) from 31% to 60%, health benefits increased 5-fold (Text S1).

As a greater proportion of routine deliveries shifted from home to facilities, we assumed 70% would shift to birthing centers or health centers staffed by SBA and 30% to facilities with full EmOC capacity. Although the differential benefits of routine delivery in birthing/health centers versus bEmOC was dependent on expedient transfer from a center to referral EmOC if needed, provided this was assured, both approaches were cost-effective. In contrast, when we varied assumptions about the proportion of routine deliveries in cEmOC versus bEmOC, cost-effectiveness results changed drastically; as routine deliveries shifted to cEmOC, the incremental cost-effectiveness ratios became much less attractive, ranging from US$8,300 to US$27,000 per YLS.

Table 6 shows the potential incremental benefits and cost-effectiveness of adjunctive community-based SBA-administered misoprostol for births at home and birthing centers/health centers in rural India. For all four “upgrade” strategies, additional lives could be saved; depending on the phase of improvements in intrapartum care, an additional 7%–13% of maternal deaths were prevented. Cost-savings for a single birth cohort of 15-y-old girls (2010) expected to accrue over their reproductive lifespan (age 15–45 y) ranged from US$128 million to US$190 million.

**Table 6:** Incremental benefits of community-based misoprostol in rural India.

Rural India	Family Planning^a^ (%)	Safe Abortion (%)	Facility Birth (%)	Transport-Home^b^ (%)	Transport-Facility^b^ (%)	Quality of Care^c^ (%)	Incremental Benefits and Cost-Effectiveness of Community-Based Misoprostol^d^		
							Decrease in Maternal Deaths	Lives Saved with Addition of Community-based Misoprostol^e^	Cost-Effectiveness
**Upgrade 1**	56.7	50	45	50	65	70	**—**	**—**	**—**
**Plus community intervention** d	**—**	**—**	**—**	**—**	**—**	**—**	12.3%	16,992	Cost saving^e^
**Upgrade 2**	60.3	60	60	60	75	80	**—**	**—**	**—**
**Plus community intervention** d	**—**	**—**	**—**	**—**	**—**	**—**	13.0%	17,612	Cost saving^e^
**Upgrade 3**	64.0	75	75	70	85	90	**—**	**—**	**—**
**Plus community intervention** d	**—**	**—**	**—**	**—**	**—**	**—**	10.2%	13,983	Cost saving^e^
**Upgrade 4**	67.6	95	80	75	95	95	**—**	**—**	**—**
**Plus community intervention** d	**—**	**—**	**—**	**—**	**—**	**—**	6.9%	9,470	Cost saving^e^

a Family planning refers to contraceptive use for limiting and spacing; shown are values representing the reduction in unmet need by 25%, 50%, 75%, and 100% with upgrades 1, 2, 3, and 4, respectively.

b Transport encompasses the expedient availability of means of transport (e.g., vehicle, cart), fuel (if needed), driver, and interim attendant care.

c Quality refers to the availability and quality of services at EmOC facilities, including adequate staffing and supplies, and evidence-based clinical practices.

d Community-based interventions assume SBA-administered misoprostol for births at home and birthing centers/health centers with a 50% (25%–60%) reduction in PPH [72].

e Population-level incremental benefits (lives saved) associated with the community-based misoprostol intervention (compared to the same strategy without the community-based misoprostol intervention). These were calculated by applying model-projected outcomes to population-level data from rural India [87]. Cost-savings for a single birth cohort of 15-y-old girls (2010) expected to accrue over their reproductive lifespan (age 15–45) ranged from US$128 million to US$190 million.

## Discussion

We have identified several strategic options that would cost-effectively reduce maternal mortality in both rural and urban India. Our principal findings are that early intensive efforts to improve family planning and provide safe abortion, accompanied by a systematic stepwise effort to scale up intrapartum and EmOC, could reduce maternal mortality by 75%. Despite the inherent uncertainty in data and assumptions used in the analysis, four critical themes emerge as robust.

First, increasing effective family planning is the most effective individual intervention to reduce pregnancy-related mortality. If the unmet need was met in rural and urban India by 2012, our results imply that the lives of 168,000 women would be saved by the end of 2015. The cost savings over that time period would exceed US$1 billion. Because strategies to increase contraceptive options for limiting and spacing do not require the same level of infrastructure as improving intrapartum care, targeting these strategies toward rural areas with high TFRs is a promising way to initiate equitable improvements in maternal health.

Second, two distinct—yet synergistic—approaches, family planning and safe abortion, can reduce deaths from unsafe abortion. Enhanced access to family planning by itself reduces demand for elective abortion and consequently reduces deaths attributable to unsafe abortion. In fact, reducing the unmet need for contraception can prevent one of every two abortion-related deaths. Furthermore, just a fraction of the cost savings from family planning would fully fund an intervention to provide safe abortion and postabortion care.

Third, despite the substantial health and economic benefits associated with family planning and safe abortion, there is a threshold above which further reductions in mortality are impossible. MDG 5 will therefore not be achievable without involving integrated interventions that ensure reliable access to high-quality intrapartum and EmOC. These interventions could be implemented, however, in a staged, scale-up fashion.

While formidable effort and financial investment would be required to scale up maternal health services over time, we identified a number of phased approaches that would ultimately prevent four out of five maternal deaths. Coupled with stepwise improvements in family planning and safe abortion, these approaches incrementally shifted home births to birthing centers or facilities with EmOC, and improved both access to SBAs as well as accurate recognition of referral need, transport, and availability/quality of EmOC. Successful implementation of these strategies would be expected to dramatically reduce the MMR, proportionate mortality ratio, and lifetime risk of maternal death.

Fourth, despite the possible variation in pace associated with scaling up maternal health services in India, systematic and consecutive phases will be cost-effective. Our results showed that—when coupled with family planning and safe abortion—both early initial strategies and late intensive strategies resulted in cost-effectiveness ratios that were just a fraction of India's per capita GDP; these would unarguably be considered very cost-effective [95]–[98].

Although our general findings are consistent with earlier suggestions that interventions to reduce maternal mortality are good public health investments [7],[17], our analytic approach also allowed us to identify more and less efficient ways to achieve this. In this regard, we highlight four robust insights: (1) In settings with limited infrastructure, investing in “intermediate” facilities (e.g., birthing centers) is very cost-effective, provided there is reliable referral capacity and transport to an appropriate EmOC facility if necessary; (2) A strategy of routine hospital-based delivery (i.e., cEmOC) is not cost-effective; (3) A community-based strategy that includes SBA-administered oral misoprostol in homes and birthing centers is likely to be cost-effective. Although alone it cannot substitute for reliable intrapartum care and EmOC, if added to a long-term plan that increases facility-based intrapartum care, it will save additional lives and will reduce costs; (4) Because settings with the highest TFRs and worst maternal health indicators also tend to be those with the greatest need for enhanced health delivery infrastructure, early consistent commitments to provide family planning and safe abortion reduce the total resources required. To place the synergistic benefits of enhanced family planning and safe abortion in context, the magnitude of cost savings from eliminating unmet need and ensuring access to safe abortion is approximately 25% of the required 10-y investment estimated by Johns et al. for scaling up maternal services in India [30].

We may have underestimated both effectiveness and cost-effectiveness by excluding effects of certain indirect indicators and interventions. Although we purposely focused on maternal mortality, if we included neonatal health and survival, for example, most strategies would be even more cost-effective due to associations between place of birth and presence of a skilled attendant, with neonatal and maternal deaths [99],[100]. With the exception of anemia, we focused on direct causes of maternal mortality; a priority for future analyses is to include interventions to reduce the indirect causes of pregnancy-related mortality. The analysis would be strengthened by availability of indicators that reflect safe motherhood externalities including measures of enhanced household well-being, increased school attendance, decreased numbers of orphans, and reduced impoverishment resulting from catastrophic expenses [94],[99].

Other limitations in our analysis stem from its inherent reliance on high-quality data about maternal mortality specifically. While our calibration of setting-specific models allows us to better represent within-country differences in baseline risk, coverage, and capacity than previous studies, high-quality empiric evidence for the effectiveness of comprehensive strategies to reduce maternal mortality and morbidity is often either lacking or inconsistent. More studies quantifying the benefits of community-level interventions on preventing maternal morbidity remain a priority [100]. The additional costs that we assumed would be required to scale up interventions and build infrastructure are, at best, gross estimates. That being said, our assumption of 2- to 3-fold increases in the per woman costs to reflect the additional resources required to improve capacity is consistent with those implied by recent analyses assessing global resource needs for maternal health [30],[93].

Shiffman and Smith [101] have described the importance of framing priority public health issues in a manner that resonates with both the “internal” community and other “external” decision makers. With regard to the internal community, the strategies we identified as most effective support three crucial elements already recognized as essential to achieve MDG 5: family planning and control of fertility choices, provision of safe abortion, and assurance that all women have access to intrapartum care and EmOC. Our results reinforce this message, and extend it by quantifying the cost savings of family planning and safe abortion, and identifying efficient and cost-effective approaches to scaling-up capacity for integrated maternal health services.

With regard to the external community, we have tried to provide a range of outcomes that can be used to create effective “take home” messages for different target audiences. For example, in only 5 y, more than 150,000 lives could be saved just from increasing contraception rates by a few percentage points; nearly US$1.5 billion could be saved by adding safe abortion to family planning efforts; and finally, with stepwise investments to provide facility-based intrapartum care, the majority of maternal deaths could be prevented. In the next decade, this accomplishment would save the lives of 1 million Indian women.

Finally, by placing and prioritizing safe motherhood in the context of other global health priorities [101],[102], our results can also be effectively framed for policymakers who must allocate limited resources, by providing comparative and contextual information about the relative benefits and cost-effectiveness of investments in maternal health measured against other public health priorities. One of the robust findings of our analysis, for example, is that there are integrated strategies that involve improvements in family planning, safe abortion, and intrapartum care that are equally or more cost-effective or attractive than childhood immunization or treatment of malaria, tuberculosis, or HIV [95].

The Indian government has initiated several policies to improve maternal health [5], particularly in rural areas [27],[103], and efforts to both implement and evaluate new strategies are ongoing [83],[94],[104]–[106]. Although our analysis is intended to catalyze actionable steps, we recognize that decisions in India about the choice of strategies and rate of stepwise investments to reduce maternal mortality will be a function not only of cost-effectiveness and affordability, but also of political will and local circumstances. Identifying approaches that can be tailored to local situations, but that rely on firm core principles and are cost-effective, holds considerable promise as a way to mobilize further political support and convince stakeholders that MDG 5 is within reach.

In particular, it is clear from our analysis that an initial focus on family planning, especially in rural poor areas, will significantly prevent pregnancy-related deaths, reduce deaths from unsafe abortion, and save resources. Providing universal access to safe abortion will further augment these benefits. The cost savings from these two strategies will partially offset the resources required to invest in the necessary infrastructure that would assure every woman access to high-quality intrapartum care and EmOC. While MDG 5 is unlikely to be met without assuring access to health-center–based intrapartum care, implementation of a phased stepwise approach, designed to reach this goal while reflecting the current realities and most feasible initial approaches in different settings, is absolutely within reach.

## Supporting Information

Text S1 Supplemental material accompanying the article. Part I, overview of model; part II, overview of model parameterization, calibration, performance; part III, overview of costs and estimates; part IV, supplemental results; part V, references.
(0.53 MB PDF)

## Abbreviations

bEmOC: basic emergency obstetrical care
cEmOC: comprehensive emergency obstetrical care
EmOC: emergency obstetrical care
GDP: gross domestic product
MDG: Millennium Development Goal
MMR: maternal mortality ratio
PPH: postpartum hemorrhage
SBA: skilled birth attendant
TFR: total fertility rate
YLS: year of life saved
