# Supplementary material for: Alternative Strategies to Reduce Maternal Mortality in India: A Cost-Effectiveness Analysis
Source: PLoS Med. 2010 Apr 20;7(4):e1000264. doi: 10.1371/journal.pmed.1000264 (PMC2857650; doi:10.1371/journal.pmed.1000264)
Supplement: Text S1 — Supplemental material accompanying the article. Part I, overview of model; part II, overview of model parameterization, calibration, performance; part III, overview of costs and estimates; part IV, supplemental results; part V, references. (0.53 MB PDF) [file pmed.1000264.s001.pdf]

Supplemental Material

Accompanying the manuscript:

HEALTH AND ECONOMIC OUTCOMES OF STRATEGIES TO REDUCE MATERNAL MORTALITY IN INDIA: IS MDG5 ACHIEVABLE?

Sue J. Goldie, MD, MPH
Steve Sweet, MBA
Natalie Carvalho, MPH
Uma chandra mouli Natchu, MD, MPH
Delphine Hu, MD, MPH

Table of Contents

Part I: Overview of Model

Part II: Overview of Model Parameterization, Calibration, Performance

Part III: Overview of Costs and Estimates

Part IV: Supplemental Results

Part V: References

Part I: Overview of Model

Analytic Overview

The best available data were synthesized using a computer-based model to assess the costs and health outcomes of different strategies to reduce disability and death due to pregnancy-related complications in India. The model captures the natural history of pregnancy and relevant co-morbidities in an individual woman, aggregates clinical outcomes to the population or subgroup level, and reflects setting-specific epidemiology, and access to health care through factors such as infrastructure, human resources, technology, health facilities and transport.   We prioritized data from India or South Asia to estimate initial ranges for age-specific probabilities of pregnancy, miscarriage, abortion, risk of maternal complications, and case-specific fatality and morbidity rates. Separate models were adapted to urban and rural India by superimposing data on coverage rates for prenatal care, antenatal care, family planning, facility births and skilled birth attendants (SBAs). After integrating assumptions on the availability of transport, facilities, and quality of care, model-projected outcomes (e.g., maternal mortality ratio [MMR], total fertility rate [TFR]) are compared with available data.

Strategies relied on improving coverage of effective interventions and providing access to key services. Interventions could be provided individually, paired, or packaged into a bundle of integrated services; phased approaches involved scaling up access to services over time. Model outcomes include clinical events (e.g., postpartum hemorrhage), aggregate population measures (e.g., life expectancy), and economic costs (e.g., average per person lifetime costs). Monte Carlo simulation was used to track the number of per-woman events such as pregnancies, live births, facility-based births, and maternal complications, allowing estimation of measures and indicators such as TFR, MMR, proportionate mortality ratio (proportion of deaths among women aged 15-45 that are pregnancy-related), and lifetime risk of maternal death.

We conducted national and state-level analyses, stratified by rural and urban status, to explore alternative approaches in settings that differ according to underlying maternal risk, health and socioeconomic status, access to health providers, means of referral and transport, and availability of facilities capable of providing different levels of emergency care. Following the reference case recommendations of the Panel on Cost-Effectiveness in Health and Medicine, as well as guidelines from the DCP2 and WHO, strategies are first ranked in terms of increasing costs and benefits. Strategies are considered “inefficient” or dominated if they were more costly and less effective, or more costly and less cost-effective, than an alternative strategy. Strategies that cost less than the status quo are considered ‘cost saving’. Incremental cost-effectiveness ratios are calculated for *all other* strategies (those that are not cost saving and those that are not dominated). The incremental cost-effectiveness ratio is defined as the additional cost of a specific intervention(s) divided by its additional clinical benefit compared with the next least expensive strategy. [DCP2, WHO CHOICE] Sensitivity analyses were conducted to assess the impact of parameter uncertainty on our results.

The Model

The Global Maternal Health Policy Model is a computer-based model that simulates the natural history of pregnancy (both planned and unintended) and pregnancy- and childbirth-associated complications. This model defines health states to reflect important characteristics that affect prognosis, quality of life, and resource use. The time horizon incorporates a woman’s entire lifetime and is divided into equal time increments during which women transition from one health state to another. Non-pregnant girls enter the model and in each time period may become pregnant depending on age, use of contraception, and clinical history. (**Manuscript Figure 1, upper panel**) Once pregnant, women have a chance of spontaneous abortion (i.e., miscarriage),

induced abortion, or continued pregnancy. A proportion of induced abortions will be unsafe (i.e., surgical or medical abortion conducted by untrained personnel). Labor and delivery may be associated with a direct complication of pregnancy (e.g., hypertensive disorders of pregnancy, obstructed labor, hemorrhage, sepsis). Case fatality rates are conditional on the type and severity of complication (e.g., moderate sepsis requiring antibiotics in bEmOC versus severe hemorrhage requiring blood transfusion in cEmOC) and underlying comorbidity. Nonfatal complications include neurological sequelae, rectovaginal fistula, severe anemia, and infertility. (**Manuscript Figure 1, upper panel**) In addition to death from maternal complications, women face an annual risk of death from age-specific all cause mortality.

Strategies in the model to reduce maternal mortality consist of improving coverage of effective interventions, which may be provided individually or packaged as integrated services. In addition to family planning, antenatal care, and safe abortion, the model includes both intrapartum interventions that reduce the incidence of a complication (e.g., misoprostol for PPH, clean delivery for sepsis) as well as those that reduce the case fatality rate through appropriate management in a referral facility **(Manuscript Figure 1, Upper Panel).**

The effectiveness of interventions to either reduce the incidence of complications or to reduce case fatality rates associated with complications depends, in part, on access to specific services (e.g., trained SBA) and to specific levels of facilities (e.g., cEmOC capacity for blood transfusion). Accordingly, the ultimate impact of interventions depends on several setting-specific factors. These include delivery site, presence of birth attendant, quality and type of referral facility, as well as successful referral when necessary. The model therefore explicitly considers the location of delivery, type of assistance, access to basic or comprehensive obstetrical care, and the ability to overcome a series of barriers around the timing of delivery (e.g., recognition of referral need, reliable transport, timely treatment at an appropriate facility); these factors collectively determine the health services a woman can access and the specific interventions that would be included. (**Manuscript Figure 1, Lower Panel**)

Delivery setting is differentiated by provider (e.g., family member, traditional birth attendant [TBA], or skilled birth attendant [SBA]) and by site (e.g., home versus facility). Facility levels are categorized as (1) birthing centres or health centres, which cannot provide all services necessary to qualify as a basic emergency obstetrical care (bEmOC) facility, but are staffed with SBA who provide expectant management of labor and more reliable referral when necessary than with delivery at home; (2) facilities with bEmOC capacity (e.g., first referral units); and (3) facilities with comprehensive emergency obstetrical care (cEmOC) capacity (e.g., district hospitals) [IIPS 2005, Government of India 2008a]. Facilities capable of bEmOC are assumed to be capable of administering injectable antibiotics, oxytocics, and sedatives or anti-convulsants, performing manual removal of placenta, removal of retained products, and assisted vaginal delivery. Facilities capable of cEmOC also are able to provide blood transfusion, cesarean section, and management of advanced shock.

This model also allows us to evaluate phased approaches that involve scaling up access to services over time; we designate such stepwise investments in infrastructure as “upgrades”. In addition to reducing unmet need for family planning and unsafe abortion, these strategies incrementally shift home births to facilities, increase skilled attendants, and improve access to, and quality of, emergency obstetrical care. For women delivering at home or in birthing centres, these strategies also improve recognition of referral need, access to transport, and expedient referral to an appropriate facility. **(Manuscript Figure 2)**.

The facility categories are flexibly modeled such that particularities of the public health infrastructure [IIPS 2005; Government of India 2008a] in different settings (country, state, rural

versus urban areas) can be accurately represented in terms capacity and cost. Facility levels (India Facility Survey Phase-II [2003]) are categorized as (1) *primary-level facilities,* which may not have all bEmOC functions but could function as birthing centres with SBA staffing, 24-hour intrapartum care, and reliable referral connections (e.g., subcentre, primary health centre [PHC]); (2) *secondary facilities* with bEmOC capacity (e.g., first referral unit [FRU], community health centre [CHC]; and (3) *tertiary facilities* with cEmOC capacity (e.g., district hospital, some first referral units) [India Facility Survey Phase II, 2003].

Framework to differentiate facilities

Health facilities in India*	Level	Staff	Model Category	Model Assumptions
District hospital	3rd	Specialists, Ob/Gyn	Tertiary facility with cEmOC	Specialists, obstetricians Active-management of labor, availability blood transfusion, surgery (e.g., c-section), intensive hemodynamic support
First referral unit	2nd	Med Officers, Specialists, Ob/Gyn	bEmOC	SBA active-management of labor
Community health centre	2nd	Med Officers, Specialists, Ob/Gyn		
Primary health centre	1st	Med Officers, Staff Nurse	Health centre (HC) or birthing centre (BC)	SBA expectant-management of labor
Subcentre	1st	Female and Male Health Workers		
*	Public-health facilities in India as categorized in the India Facility Survey Phase-II [2003]			

We recognize that some tertiary sites will not have a blood bank and some secondary sites may eventually be able to perform c-section; further, we recognize that in the strategies that include stepwise investments in infrastructure and facility improvements, not all facilities will be expected to be fully implemented as one of the three distinct types. However, because the costs, functions and staffing are fairly closely aligned with basic or comprehensive EmOC capacity, this simple categorization captured the most important dimensions for purpose of this analysis. Above is a stylized example of how public health facilities in India, as categorized in the India Facility Survey Phase-II [2003], may be superimposed on our general model framework.

All models were built using TreeAge Pro 2008 (TreeAge Software Inc., Williamstown MA) and analyzed using IBM/Lenovo Dual-Core VT Pro Desktop computers running Microsoft Windows XP, using Microsoft Excel 2007 and Visual Basic for Appplications 6.5 (Microsoft Corp., Redmond WA). We used Monte Carlo simulation to generate the number of per woman events such as pregnancies, live births, facility-based births, and maternal complications. This output is useful for both calibration exercises, as well as for assessing internal consistency and projective validity of the model by generating outcomes in similar formats to clinical studies. We use second order Monte Carlo simulation to assess parameter uncertainty.

Part II: Overview of Model Parameterization, Calibration, Performance

Subsection A

Data and Assumptions: Initial Natural History Parameters. The best available data are sought on clinical parameters governing the natural history of pregnancy. Examples of required model inputs include the age-specific probability of pregnancy, miscarriage, unsafe and safe abortion; incidence, morbidity, and case fatality rates for each maternal complication (PPH, sepsis, obstructed labor, hypertensive disorders of pregnancy); prevalence of co-morbidities (e.g., anemia).

Data and Assumptions: Intervention Effectiveness. The effectiveness of interventions to reduce the incidence of complications and/or reduce case fatality rates is from published data, and varies by complication type and severity. Initial estimates assume an intervention can be delivered appropriately, but are then modified according to several setting-specific factors. These include delivery site, presence of birth attendant, quality and type of referral facility, as well as successful referral when necessary

Subsection B

Data: Coverage Inputs and Selected Services. Data on coverage rates for interventions, facility births, skilled birth attendants, antenatal care, and family planning, are stratified by rural and urban status, and for validation analyses are state-specific.

Data and Assumptions: Barriers to Effective Referral. Effective referral relies on the ability to overcome three critical delays (a) recognition of referral need and willingness to be referred (by provider and delivery location); (b) expedient transfer to referral facility (determined by distance, affordability, available transport); and (c) timely treatment in an appropriate facility capable of high-quality emergency obstetrical care (e.g., 6 signal functions in bEmOC, blood transfusion and surgery in cEmOC).

Subsection C

Calibration Exercises. Calibration targets include the distribution of causes of maternal mortality (e.g., PPH, obstructed labor, sepsis), maternal mortality ratio (MMR), total fertility rate (TFR). After integrating assumptions about the availability of health services, model-projected estimates of MMR, TFR, and distribution of direct causes of maternal mortality are compared to empiric data. Selected uncertain parameters (such as the case fatality rates conditional on severity of complication) are varied across a pre-specified plausible range, in a systematic fashion, to ensure output is consistent with key empiric indicators.

Model Performance. Model performance is assessed by comparison of model-based projections with independent measures such as life expectancy, proportionate mortality ratio, and population-based outcomes. Projective validity of the empirically-calibrated model is further assessed by simulating two states, and comparing projected maternal health indicators with reported data

Subsection A

Data and Assumptions

Age-specific probability of pregnancy

To estimate a fertility rate in the absence of any family planning, we use data from Afghanistan [Amowitz 2002, Bartlett 2005], where contraceptive use is low, the maternal mortality ratio (MMR) is among the highest in the world (~1,600 deaths per 100,000 live births), and access to modern health care is limited (< 5%) [AbouZahr 2004]. We synthesized data on family planning, abortion, and demographics (i.e., crude birth rate in Afghanistan of 308 pregnancies per 1,000 population) to approximate an average annual natural fertility rate of 31% [PRB 2004].The model allows for age-specific inputs for fertility and use of contraception. We assumed 15% of all pregnancies end in spontaneous abortion, of which approximately one-third result in incomplete abortion requiring medical intervention [Harlap 1980, Menken 2006]. We assumed women with long-term complications such as infertility or untreated obstetric fistula did not become pregnant again. We assumed women with complications that were treated (e.g., severe anemia, surgically repaired fistula) could become pregnant again.

Anemia

In the National Family Health Survey-3 (2005-2006), among women of reproductive age, the overall prevalence of anemia was 52%, with mild anemia affecting 35%, moderate anemia affecting 15%, and severe anemia affecting 2%. [IIPS 2007] In Uttar Pradesh (UP), based on data from the District Level Household & Facility Survey of the Reproductive & Child Health Project (DLHS-RCH phase-2, round-2 survey), anemia accounted for 55% of all indirect causes of death, and 15% of all maternal deaths. [Mills 2007] The relative risk of death from maternal complications is 3.5 times greater with severe anemia and 1.35 times greater with moderate anemia, compared to a woman without anemia. [Brabin 2001] Antenatal care presents an opportunity to detect and treat anemia. We assumed severe and moderate anemia were associated with a higher relative risk of death from pregnancy- and delivery-related complications, although anemia differentially affected mortality from postpartum hemorrhage and sepsis, and complications following unsafe abortion. We conservatively assumed that severe anemia did not impact the case fatality rate of untreated obstructed labor.

Anemia prevalence by state for selected states (NFHS-3) [IIPS 2007]

	Ever-married women (aged 15-49) who are anemic (%)	Currently pregnant women (aged 15-49) who are anemic (%)
**India**	56.2	57.9
Rajasthan	53.1	61.2
Uttar Pradesh	50.8	51.6
Bihar	68.3	60.2

Prenatal care and treatment of anemia, NHFS-3 [IIPS 2007]

	**India**		**UP (State)**		**Rajasthan (State)**	
Prenatal care (%)	50.7		26.3		41.2	
Tx for anemia (%)	22.3		8.7		13.1	
	**Urban**	**Rural**	**Urban**	**Rural**	**Urban**	**Rural**
Prenatal care (%)	73.8	42.8	40.9	22.6	74.7	31.7
Tx for anemia (%)	34.5	18.1	16.4	6.7	30.2	8.3

Sexually-transmitted diseases

The overall risk of gonorrhea/chlamydia was estimated at 7.2%, with 25% resulting in symptomatic lower genital tract infection and a 30% risk of pelvic inflammatory disease (PID), of which 40% was symptomatic and 25% led to infertility [WHO 2007a].

Pregnancy-related complications

Incidence, case fatality rates, interventions to reduce incidence and mortality

Initial estimates of incidence and case fatality rates associated with pregnancy-related complications were obtained from published data, and a plausible range for sensitivity analysis was based on systematic review of the literature. Case fatality rates were adjusted based on complication severity (e.g., life threatening complications requiring cEmOC) and underlying severity of anemia, using methods described below. The effectiveness of interventions to reduce the *incidence of complications* (e.g., active management of labor) was estimated from published studies using methods described below. The effectiveness of interventions to reduce *case fatality rates* were from published studies and assumed treatment in an appropriate facility; a wide plausible range was used for sensitivity analyses, as described below. The effectiveness of interventions to either reduce the incidence of complications or to reduce case fatality rates associated with complications depend, in part, on access to specific services (e.g., trained SBA) and to specific levels of facilities (e.g., cEmOC capacity for blood transfusion). The ultimate impact of interventions therefore depends on several setting-specific factors. These include delivery site, presence of birth attendant, quality and type of referral facility, as well as successful referral when necessary. Data on facility births, skilled birth attendants, family planning for spacing or limiting births, and antenatal care were from country-specific surveys, and are described in Subsection B. A summary table is provided below, and the sections that follow provide information on the source and rationale for the baseline initial estimates, the adjusted estimates and the range for sensitivity analyses.

Overview of Parameters and Ranges Evaluated in Sensitivity Analysis*

	Hemorrhage	Obstructed Labor	Hypertensive disorders	Sepsis	Unsafe Abortion
**Complications (incidence, case fatality rate)**					
Incidence	0.114	0.047	0.035	0.050	0.128
Range	0.051 – 0.228	0.030 – 0.074	0.025 – 0.050	0.043 – 0.060	0.050 – 0.250
CFR * (initial)	0.010	0.007	0.017	0.013	0.003
Range	0.007 – 0.013	0.005 – 0.009	0.012 – 0.022	0.009 – 0.017	0.002 – 0.004
CFR* (adjusted)	**0.023**	**0.019**	**0.021**	**0.028**	**0.009**
Range	0.016 – 0.030	0.013 – 0.025	0.015 – 0.027	0.020 – 0.036	0.006 – 0.012
Range used*	**0.007 – 0.030**	**0.005 – 0.025**	**0.012 - 0.027**	**0.009 – 0.036**	**0.002 – 0.012**
**Effectiveness (prevention, management and treatment)**					
↓ Incidence	50%, 75%	----	----	25%, 50%	----
Range	25% - 91%	----	25%-50%	0% - 60%	0% - 100%
↓ CFR	75%	95%	59%	90%	98%
Range	60% - 90%	76% - 100%	45% - 95%	63% - 93%	50% - 100%

Adjustment of case fatality rates for heterogeneity in severity and co-morbidity

Baseline estimates for cause-specific case fatality rates were from a review conducted by the Disease Control Priorities Project (DCP2). [Graham 2006] - maternal hemorrhage (1%), sepsis (1.3%), hypertensive disorders of pregnancy (1.7%), obstructed labor (0.7%) [Graham 2006]. These case fatality rates are lower than those reported in West Bengal, India which ranged from 0.9% to 3.5%, implying they could be underestimates [Biswas 2005]. We assumed that some of the variation reported in the literature is attributable to the heterogeneity in severity, and estimates based on small sample sizes. Case fatality rates were thus adjusted based on complication severity (e.g., life threatening complications requiring cEmOC) and underlying severity of anemia. We assumed that life-threatening complications requiring cEmOC-level services were associated with a higher case fatality rate (in the absence of treatment) than that of non-life-threatening complications. This relative risk was determined through a number of calibration exercises in which the model was first parameterized using the best natural history data available, then adjusted to reflect the current standard of care in India, and finally by allowing this relative risk to vary such that the model fit multiple epidemiologic targets simultaneously, including the MMR, life expectancy, total fertility rate, and distribution of maternal mortality causes. We additionally assumed an increased relative risk of mortality secondary to severe maternal hemorrhage, sepsis and abortion, ranging from 1.5 to 3.0 with severe anemia [Brabin 2001] Assumptions for the proportion requiring bEmOC and cEmOC are described below.

Among the estimated 15% of pregnant women in developing countries who experience pregnancy-related complications, 7% require care at centers with surgical capacity (cEmOC) and 2-3% will require surgery [Johns 2007]. Initial estimates of the proportion of complications requiring basic versus comprehensive EmOC care were derived from a study providing WHO expert opinion-based estimates, for each maternal complication, of the proportion of complications that will require surgical intervention, blood transfusion, or management of shock [Johns 2007].

Johns (2007)	Require bEmOC	Require cEmOC
Hypertensive disorders	85.8%	14.2%
Obstructed labor	8% (assisted delivery)	92% (cesarean section)
Postpartum hemorrhage	72.5%	27.5% (25% transfuse, 2% surgery)
Puerperal sepsis	63%	10% transfusion; 27% shock

In setting-specific models (e.g., rural versus urban India), these proportions were slightly altered either using data about the availability of certain levels of care in basic and comprehensive facilities and extent of training of personnel, or from insights gained during the calibration exercises. (Subsection B and C). In certain cases, we had specific data to assist with modifying estimates. For example, Johns et al assumed 85.8% of eclampsia cases (HTN) require bEmOC, while 14.2% require cEmOC [Johns 2007]. This estimate is similar to an earlier study which showed that severe pre-eclampsia and eclampsia required treatment with intravenous hydralazine and magnesium sulfate and in addition, approximately 10% of all cases were assumed to require emergency cesarean section [Cahuana-Hurtado 2004]. Initial assumptions (and ranges) used in India with respect to severity and need for basic versus comprehensive EmOC are provided below; sensitivity analyses were conducted to assess the implications of using the upper and lower bounds.

Assumptions about complications requiring bEmOC and cEmOC in India (used in model)

Complication	Require bEmOC	Require cEmOC
Hypertensive	85.8% (75%-90%)	14.2% (10%-25%)
Obstructed labor	31% (5%-35%)	69% (65%-95%)
Postpartum hemorrhage	65.6% (60%-72.5%)	34.4% (27.5%-38%)
Puerperal sepsis	63% (60%-75%)	37% (25%-40%)

Using the assumptions above we began with the literature-based case fatality rates and then adjusted these based on severity (e.g., necessity for cEmOC, need for transfusion, delay in reaching care, underlying moderate anemia, underlying severe anemia). For example, to calculate implied average CFR for PPH, we took the baseline CFR of 1%, applied relative risks based on severity (e.g., necessity for cEmOC, need for transfusion, delay in reaching care, underlying moderate anemia, underlying severe anemia) and then weighted these based on the percentage of women who would face that risk. Therefore, the implied average CFR in India for maternal hemorrhage of 2.11% is calculated from the following - 48.6% (CFR 1%), 15% (CFR 1.5%), 2% (CFR 3%), 5.2% (CFR 5.25%), 28.5% (CFR 3.5%), less than 1% (CFR 10.5%). We then expanded the plausible range for sensitivity analysis based on this adjusted CFR. For example, a literature based range based on 0.01 (1%) average CFR was 0.007 – 0.013. The expanded plausible range based on our adjustment was 0.007 – 0.030.

Adjusted CFR and Expanded Range for Sensitivity Analysis*

	Hemorrhage	Obstructed Labor	Hypertensive disorders	Sepsis	Unsafe Abortion
**Case fatality rates in the absence of interventions**					
CFR * (initial)	0.010	0.007	0.017	0.013	0.003
Range	0.007 – 0.013	0.005 – 0.009	0.012 – 0.022	0.009 – 0.017	0.002 – 0.004
CFR* (adjusted)	**0.023**	**0.019**	**0.021**	**0.028**	**0.009**
Range	0.016 – 0.030	0.013 – 0.025	0.015 – 0.027	0.020 – 0.036	0.006 – 0.012
Range used*	**0.007 – 0.030**	**0.005 – 0.025**	**0.012 - 0.027**	**0.009 – 0.036**	**0.002 – 0.012**

Hemorrhage

Incidence and case fatality rate

In a systematic review of 34 datasets, representing over 35,000 maternal deaths, Khan et al. [2006] found hemorrhage to be the leading cause of death throughout the world, accounting for a range of 1.4% to 49.6% of all maternal deaths, and the cause of the highest proportion of deaths in Asia and Africa. In India, hemorrhage is the leading cause of maternal mortality and contributes up to 23% of all maternal deaths (range, 7-30%). [Khan 2006] Maternal hemorrhage is categorized according to its timing in relation to delivery: antepartum, intrapartum, or postpartum. The etiologies and management of maternal hemorrhage differ among these three categories.

Initial estimates for the overall incidence of PPH were based on data from the WHO's Global Burden of Disease study (2002) specifically for the SEAR region. We calculated estimates for the incidence of PPH (0.097625) using data on the number of cases (n=3,692,000) [WHO 2007a] and the total number of births (n =37,820,000) [UNICEF 2004]. The risk of PPH was modified to reflect assumptions in the WHO's "Global burden of maternal hemorrhage in the year 2000" [Dolea 2003a], specifically that the incidence of PPH, defined as > 1000ml of blood loss in the oxytocin arm, was 2.85% within 1 hour postpartum in women who were actively managed, as estimated

from the MISO trial. [Dolea 2003a] We assumed that the incidence of PPH in women who are managed expectantly by a skilled birth attendant would be twice as high as found in the MISO trial, or 5.7% of births. [Chandhiok 2006, Dolea 2003a, Gulmezoglu 2001], and that births without skilled attendance would be twice as high as those with skilled birth attendance. [Alfirevic 2007, Derman 2006, Gulmezoglu 2007, Hoj 2005, Walraven 2005] Extrapolating from these data, we assumed all births in a facility with emergency obstetrical care would be actively managed with a 2.85% risk of PPH, all other births with a skilled attendant would be expectantly managed with a 5.7% risk of PPH, and all births attended by a family member or traditional birth attendant, or when delivery was alone, would be associated with an 11.4% risk of PPH. This approximates the range of 8% to 15% reported in the literature.

The initial estimate for the case fatality rate (CFR) was from a review conducted by the Disease Control Priorities Project (DCP2) [Graham 2006] who reported an average CFR of 1%; this estimate was adjusted according to case severity and underlying morbidity (e.g., severe anemia) by calibrating the model to fit multiple epidemiologic targets simultaneously. See section above on Adjustment of case fatality rates for heterogeneity in severity and co-morbidity. The implications of our adjusted CFR widened the implied plausible range. While the literature based range based on 0.01 (1%) average CFR from DCP2 was 0.007 – 0.013, the expanded plausible range based on our adjustment of a CFR of 0.023 (2.2%) was 0.007 – 0.030. Model projected mortality due to maternal hemorrhage, as well as MMR, TFR, and calendar deaths for 2005, closely approximated the empiric data.

To account for the uncertainty in our initial estimates, we established a plausible range for all the above parameters based on our literature review. There have been multiple studies, including 8 systematic reviews, of the incidence and case fatality rate of maternal hemorrhage. A Cochrane review demonstrated that active management with oxytocin results in a relative risk of 0.33 for blood loss >1000ml within the first 24 hours, compared to expectant management. [Prendiville 2000] This review has since been withdrawn due to concerns regarding the validity of these findings. [Prendiville 2009] An updated analysis is currently underway to ensure the use of more recent data. A Cochrane review comparing oxytocin to no uterotonics found an overall reduction in blood loss > 1000ml of 39% (RR 0.61). [Cotter 2001] Comparing oxytocin to no uterotonics when active management was used in both trial arms, the same review found a relative risk of 0.33, and when expectant management was common to both trial arms, a relative risk of 0.73 was found. [Cotter 2001] A study published in 2002 comparing active management with expectant management found a relative risk of 0.8 for blood loss > 500ml. [Geelhoed 2002] While severe PPH was not an endpoint captured in this trial, we would expect a slightly lower RR for blood loss > 1000ml. A more recent randomized control trial of a small number of women who gave birth at a maternity unit in Iran comparing active management with oxytocin to expectant management found conflicting evidence regarding the optimal method by which to manage the third stage of labor. [Kashanian *In press*] This trial found that active management did not decrease blood loss during the third stage of labor but did decrease the duration of this stage. Active management was associated with increased blood loss during the fourth stage of labor. Severe PPH was not an endpoint captured within this analysis.

In a systemic review of randomized trials, the prophylactic administration of oxytocin reduced severe PPH from 7% to 4.3% (RR 0.61), and that the relative risk of severe PPH when using misoprostol compared to placebo (when an outlier study was excluded) was 0.77 for 600 mg and 0.54 for 400 mg. Hofmeyr (2008) There was wide variation in the effect of misoprostol but all studies showed some effectiveness compared to placebo. A systemic review covering the period 1997-2006 (Carroli 2008) included 24 studies from the WHO database covering a period of 1997-2002 and an additional 166 reports assessed (from 2003-2006) with 14 included (total = 38) for a total of 224 datasets. These were stratified to those that reported PPH (n=120) and those that

reported severe PPH (n=70), with severe PPH being defined as blood loss >1,000 ml. Overall quality was deemed adequate for ~47% of PPH datasets and ~59% of SPPH datasets. Overall prevalence of PPH was 6.09% (CI 6.06 to 6.11) with 10.55% when the blood loss was measured objectively. Overall prevalence of severe PPH was 1.86% (CI 1.82 to 1.90) with 3.04% when the blood loss was measured objectively. A high degree of heterogeneity was reported even in subgroups with similar characteristics. Severe PPH was reported at 3.84% for expectant management alone (CI 3.31-4.37) in 6 datasets of 4,999 women, 2.99% (2.80-3.18) for active management alone in 21 datasets with 30,608 women, 2.47% (2.06-2.88) for uterotonic before delivery in 11 datasets with 5,585 women and 2.08% (1.39-2.77) for uterotonic after delivery in 2 datasets with 1,635 women. (**see table on next page**)

Reduction in mortality

The incidence of maternal hemorrhage is dependent on the delivery setting, the use of expectant or active management, and the use of misoprostol. The model represents a range of approaches to *reduce mortality* from PPH: first, on the basis of delivery setting and use of expectant or active management of labor; second, by successful referral and access to quality care in an appropriate facility with basic or comprehensive emergency obstetrical care; third, with temporizing measures such as the antishock garment to reduce blood loss and shock en route to an adequate facility; fourth, by the use of misoprostol after PPH at home or in a birthing centre, to reduce total blood loss; and fifth, by the use of misoprostol in the community setting, at home or in a birthing centre or subcentre, to prevent PPH. Data for the first, second and third options are far more readily available than the fourth and fifth options, which reflects our choice to focus on these for the present analysis. We include one exploratory analysis of community-based SBA-administered misoprostol. We assumed optimal treatment of maternal hemorrhage in an appropriate facility with EmOC capacity consisted of intramuscular or intravenous oxytocin immediately after delivery, uterine massage, repair of any perineal or vaginal tears, and fluid replacement or blood transfusion [Cahuana-Hurtado 2004]; consistent with assumptions made by Adam (2005) and Graham (2006) we assumed an average reduction of 75% in the CFR [Adam 2005, Gulmezoglu 2001; Graham 2006]. We varied this estimate from 60% to 90% for optimal management (i.e., bEmOC or cEmOC as necessary for severity).

Studies on maternal hemorrhage that contribute to plausible range for calibration exercises and sensitivity analyses						
	Relative Risk (RR)			% Reduction in Risk		
	RR	95% CI		%	lower	upper
**Strategies to reduce incidence of maternal hemorrhage**						
**Active management of labor [**Prendiville 2003, Chandhiok, Derman, Hoj, Walraven, Gulmezoglu, Dolea]						
Active management with oxytocin vs. expectant management for incidence of severe PPH in 1st 24 hrs	0.50			50%		
Prendiville Cochrane 2000 - active mngmt with oxytocin vs. expectant mngmt for incidence of severe PPH in 1st 24 hrs	0.33	0.51	0.21	67%	49%	79%
Prendiville Cochrane 2000 - active mngmt with oxytocin vs. expectant mngmt for incidence of PPH (>=500ml) in 1st 24 hrs	0.38	0.46	0.32	62%	54%	68%
Prendiville Cochrane 2000 - active mngmt with oxytocin vs. expectant mngmt for incidence of secondary PPH (24hrs - 6 weeks)	0.88	1.60	0.49	12%	-60%	51%
Chandhiok 2007 - active mngmt & miso by SBA in HC	Lower blood loss vs. control, but few PPH (0.7% vs. 0.8%)					
Dolea 2003 - GBD est. active vs. expectant mngmt, severe PPH				50%		
Cotter Cochrane 2001 - oxytocin vs. no uterotonics, severe PPH, with active mngmt for both	0.33	0.77	0.14	67%	23%	86%
Geelhoed 2002 - active vs. expectant mngmt, PPH (>=500ml) only	0.8	0.9	0.7			
Gulmezoglu 2001 - miso vs. oxytocin w/ active mngmt, severe PPH	1.39	1.69	1.19	-39%	-69%	-19%
Kashanian (in press) - active vs. expectant mngmt, no endpoints on severe PPH	Active management - no decrease in blood loss, decreased duration of 3rd stage, increased blood loss in 4th stage					
Leduc 2009 - active mngmt --> risk of PPH	No effectiveness data					
Dolea 2003 - GBD est. expectant w/ SBA vs. nothing, severe PPH				50%		
Cotter Cochrane 2001 - oxytocin vs. no uterotonics, severe PPH, with expectant mngmt for both	0.73	1.07	0.49	27%	-7%	51%
**Oxytocin**						
Cotter Cochrane 2001 - oxytocin vs. no uterotonics, severe PPH	0.61	0.87	0.44	39%	13%	56%
Cotter Cochrane 2001 - oxytocin vs. no uterotonics, severe PPH, RCT only	0.72	1.05	0.49	28%	-5%	51%
Elbourne 2001 - oxytocin vs no uterotonics, outcome of severe PPH (>=1000ml blood loss)	0.57	0.79	0.41	43%	21%	59%
Pagel 2009 (based on Hofmeyr 2008, Carroli) - oxytocin vs. no uterotonic severe PPH				61%		

Misoprostol

For the exploratory analysis evaluating the use of SBA-administered misoprostol at home and in birthing centres we varied effectiveness to prevent PPH from 10% to 75%, and conducted a baseline analysis using 25% and 50%. Among most of the studies that have assessed misoprostol, there is variation in methods and clinical practice (e.g., measurement of blood loss, management of the third stage of labor, and use of uterotonics to prevent versus manage PPH), which makes direct comparison difficult. In Gulmezoglu (2001), oxytocin was reported to be more effective than misoprostol in reducing acute and severe PPH. However, the study included several developed countries and when distinguishes the data from just the developing countries, the risks are similar for oxytocin and misoprostol. More recent studies showed more positive results in terms of measured blood loss, versus placebo, for prevention (Derman 2006, Hoj 2005) of PPH.

Derman et al (2006) reported the results of a placebo-controlled trial in rural India with auxiliary nurse midwives at home or in village subcentres who administered misoprostol 600 mcg orally or a placebo, in the context of expectant management. They found a statistically significant reduction in risk for acute PPH, severe PPH, additional uterotonics, transfer to higher level facility, and transfusion in the misoprostol group versus the placebo group. Hoj et al. (Hoj 2005) in Guinea-Bisseau, another large study (n > 600), showed a reduction in the risk for severe PPH although not acute PPH. The incidence of acute PPH (blood loss > 500) was high for both misoprostol and placebo groups (45% and 51%) as was the incidence of severe PPH (11% and 17%). Walraven et al. (Walraven 2005) reported results of a study in the Gambia comparing TBA-administered 600 mcg misoprostol to 2 mg ergometrine (n> 1000), in which both drugs had similar risks of acute PPH (11-12%) and severe PPH (0.3-0.7%), and lower blood loss compared to placebo. Chandhiok et al. (Chandhiok 2006) in rural India with n > 1000 which compared 600 mcg misoprostol orally to methergine (both IM and PO) and showed similar risks of acute PPH for both groups which were very low (0.70-0.8%).

Our review also found that providing misoprostol as a prophylaxis (as a dose of 600ug) was more effective than placebos at preventing PPH in community births, having a relative risk of 0.59, but was not as effective in a hospital setting where it had a relative risk of 1.23. [Alfirevic 2007] This was reinforced by a Cochrane Review [Gulmezoglu 2007] which concluded that while misoprostol was less effective than oxytocin and associated with higher rates of shivering and fever, it showed promising results when compared to the placebo. A review of the evidence supporting guidelines found that for prevention of PPH, active management reduced risks during the third stage of labor. It also found that misoprostol should only be used if oxytocin is unavailable [Leduc 2009]. This was supported by a study [Gulmezoglu 2009] which, cited a previous study by Prendiville et al. [2000], found that active management could reduce the risk of severe PPH by between 60% to 70%.

Hofmeyr (2008) found in a systematic review of randomized trials that there was no difference in severe morbidity between misoprostol and other conventional uterotonics, although those who were given misoprostol did experience more side effects. When an individual outlier study was excluded, results showed less blood loss with misoprostol than with the placebo provided during the trial; specifically, the prophylactic administration of oxytocin reduced severe PPH from 7% to 4.3% (RR 0.61), and that the relative risk of severe PPH when using misoprostol compared to placebo was 0.77 for 600 mg and 0.54 for 400 mg. This review did not answer the question of whether the relative mortality reduction owing to misoprostol preventing PPH-related deaths was offset by an increase in mortality caused by the drug. In addition, the review did not compare active versus expectant management [Hofmeyr 2009]

Effectiveness of Misoprostol

	Relative Risk (RR)			% Reduction in Risk		
	RR	95% CI		%	lower	upper
Pagel 2009 (from Hofmeyr 2008, Carroli) - miso vs. no uterotonic, severe PPH				77%		
Hofmeyr 2009 - miso vs. placebo, severe PPH	0.63	0.91	0.44	37%	9%	56%
Alfirevic 2007 - miso vs. inj. uter --> sev. PPH (from [Gulmezoglu Cochrane 2004])	1.34	1.55	1.16	-34%	-55%	-16%
Alfirevic 2007 - miso vs. placebo community births --> severe PPH	0.59	0.84	0.41	41%	16%	59%
Alfirevic 2007 - miso vs. placebo hospital births --> severe PPH	1.23	1.74	0.86	-23%	-74%	14%
Gulmezoglu 2007 - miso vs. placebo/no tx --> severe PPH	heterogeneity - lack conclusive evidence					
Gulmezoglu 2007 - miso vs. placebo/no tx (early trials) --> severe PPH	early trials show no reduction					
Gulmezoglu 2007 - miso vs. placebo/no treatment (later trials) --> severe PPH	0.31	0.94	0.1			
Gulmezoglu 2007 - miso vs. injectable uerotonics --> severe PPH	1.32	1.51	1.16			
Derman 2006 - RCT miso w/ SBA vs. placebo, severe PPH 2 hrs	0.20	0.91	0.04	80%	9%	96%
Derman 2006 - RCT miso w/ SBA vs. placebo, PPH (>= 500ml) 2 hrs	0.53	0.74	0.39	47%	26%	61%
Hoj 2005 - miso vs. placebo, severe PPH	0.66	0.98	0.45	34%	2%	55%
Walraven 2005 - miso vs. placebo, severe PPH	0.48	2.59	0.09	52%	-159%	91%

Inclusion of antepartum hemorrhage

Johns et al (2007) estimates antepartum hemorrhage requiring management will complicate 2.2% of pregnancies and more specifically 0.11% pregnancies will be complicated by antepartum hemorrhage requiring caesarean section and 0.726% pregnancies will be complicated by antepartum hemorrhage requiring transfusion. As with postpartum hemorrhage, the course of antepartum hemorrhage can be unpredictable and a recurrent bleed can occur at any time and any severity level. Antepartum hemorrhage was not considered as a *separate category* in our model because we felt there were insufficient data on its epidemiology, natural history, and the impact of interventions *in developing countries*. [For these same reasons, estimates of death and disability attributable to antepartum hemorrhage were not included in the WHO's global burden of maternal hemorrhage]. That being said, we *did* calibrate to observed data on the distribution of deaths by cause in India, including PPH of all causes. [see **Subsection C**.]

In contrast to postpartum hemorrhage, for which WHO region-specific incidence and mortality rates are available, the frequency of antepartum hemorrhage has been difficult to establish at the population level in developing countries due to a lack of: (1) widely accepted diagnostic criteria for this condition and (2) reliable ascertainment, which is grossly affected by the quality and availability of maternal care. Empirical data regarding the natural history of antepartum hemorrhage are also lacking. For example, the proportion of antepartum hemorrhages that present as severe or life-threatening is unknown, as is the proportion of cases that ultimately require transfusion and/or cesarean section. The percentage of cases of antepartum hemorrhage that resolve only to recur later on is also unknown. In addition, the mortality or morbidity risk of antepartum hemorrhage in the absence of medical care has not been determined. Finally, data are scarce with regard to the impact of interventions targeting antepartum hemorrhage. In developed countries, management of antepartum hemorrhage is frequently determined on a case-by-case basis since its etiology varies and management is dependent on multiple factors including etiology, the status of the mother and fetus, the amount of bleeding, gestational age, and in the case of placenta previa and abruption, the degree of separation between the uterus and the placenta. In developed countries, where comprehensive maternal care is not only high quality but also widely and promptly available, the mortality risk of antepartum hemorrhage has been reduced to <1%. This low mortality risk is attributable to a highly vigilant approach to this condition, generally consisting of: (1) emergency caesarean section for patients with refractory hemorrhage, poor fetal status, or significant bleeding after 34 weeks gestation; (2) hospitalization with close monitoring and supportive care for actively bleeding patients; (3) expectant management as an inpatient (or outpatient if the patient lives within 5-10 minutes of a comprehensive medical center) with close follow-up and planned caesarean section (or vaginal delivery, if possible) at 36 weeks (after documentation of fetal lung maturity) or sooner, if necessary, for patients with a resolved episode of antepartum hemorrhage due to placenta previa or abruption. The level and intensity of care required is not feasible for most developing countries. Additionally, there are currently no established guidelines or effectiveness data concerning the management of antepartum hemorrhage using a less vigilant approach in resource-poor settings.

Sepsis

Incidence and case fatality rate

Globally, puerperal sepsis and infection are estimated to contribute to nearly 10% of all maternal deaths in Africa (9.7%), Asia (11.6%), and Latin America and the Caribbean (7.7%) [Khan 2006]. In India, puerperal infection and sepsis are responsible for 11% (range, 9-14%) of all maternal deaths [Registrar General 2006]. Initial estimates for the overall incidence of puerperal sepsis were created using data from the 2002 edition of the WHO's Global Burden of Disease study specifically for the SEAR region. We calculated estimates for the observed incidence of puerperal

sepsis (0.041588) by using data on the number of cases (n=1,573,000) [WHO 2007a] and data on the number of births (n =37,820,000) [UNICEF 2004]. We base our estimates for the risk of puerperal sepsis on the 2000 GBD estimates [Dolea 2003b], that births occurring inside facilities with SBA were assumed to have a risk of puerperal sepsis of 2.5%. We assumed skilled birth attendants adhere to clean delivery practices, and therefore home deliveries attended by SBA had the same risk of puerperal sepsis. Those delivering at home with an untrained attendant had double the risk, at 5.0% [Dolea 2003b]. To account for the uncertainty in our initial estimates, we established a range of 4.2% - 6% for sensitivity analysis.

A 2004 Cochrane Review that assessed the effectiveness and safety of antibiotic prophylaxis in reducing infectious puerperal morbidities in women undergoing operative vaginal deliveries included only one trial in which women underwent vacuum or forceps deliveries. While there was no statistically significant difference in the group of women that were given antibiotics versus those not given antibiotics, there was a relative risk reduction of 93% in the prophylactic antibiotic group [Liabsuetrakul 2004]. Two studies by Mosha et al. (2000) and Winani et al. (2007) concluded that women who bathed before delivery and women who used a clean delivery kit were 2.6 and 3.2 times less likely to develop puerperal sepsis than women who did not, respectively. Other studies reported a non-significant difference or inconclusive difference in effectiveness of interventions to prevent puerperal sepsis [Goodburn 2000; Hussein 2004; Bakr 2005; Tsu 2009].

The initial estimate for the case fatality rate (CFR) was from a review conducted by the Disease Control Priorities Project (DCP2) [Graham 2006] who reported an average CFR of 1.3% and CFR 3.9% for severe sepsis; these estimates were then adjusted according to heterogeneity in severity and underlying morbidity (e.g., severe anemia) by calibrating the model to fit multiple epidemiologic targets simultaneously. See section above on <u>Adjustment of case fatality rates for heterogeneity in severity and co-morbidity</u>. The implications of our adjusted CFR widened the implied plausible range. While the literature based range based on 0.013 average CFR from DCP2 was 0.009 – 0.017, the expanded plausible range based on our adjustment of a CFR of 0.028 was 0.009 – 0.036. Model projected mortality due to sepsis, as well as MMR, TFR, and calendar deaths for 2005, closely approximated the empiric data.

Reduction in mortality

We assumed the treatment regimen for puerperal sepsis (e.g., 2-day intravenous course of ampicillin, gentamycin, and metronidazole followed by an 8-day course of intramuscular gentamycin and oral metronidazole) had an overall treatment efficacy of 90% [Adam 2005, French 2003,2004; Graham 2006]. A similar estimate was used in a recently published modeling analysis conducted by Pagel et al. (2009). Assuming an 11% case fatality rate for sepsis following delivery in sub-Saharan Africa, an 8-fold higher case fatality rate for sepsis without antibiotics compared to with antibiotics, and a 40% rate of antibiotic use, they estimated an 87.6% reduction in mortality from sepsis [Pagel 2009]. We varied this estimate from 63% to 93% for effectiveness expected in an appropriate EmOC facility.

Obstructed labor

Incidence and case fatality rate

The two major causes of obstructed labor are cephalopelvic disproportion and abnormal fetal presentation (i.e., breech or brow presentation). Major complications of obstructed labor include endometritis, rectovaginal or vesicovaginal fistula, and ruptured uterus with consequent hemorrhage, shock or death. If the obstruction cannot be resolved by manipulation (to reposition the fetus) or instrumentation (with forceps or vacuum to deliver the fetus), cesarean section is required. Globally, obstructed labor is estimated at 4.6% of live births, although varies

considerably among different regions of the world [Khan 2006, Dolea 2003c]. Approximately 5% (range 3-6%) of all maternal deaths in India are due to obstructed labor [Registrar General 2006], with the major cause being cephalopelvic disproportion. Women who are malnourished, marry young, or engage in childbearing at an early age before the pelvis has reached adult proportions, are at high risk for obstructed labor. However, there is no evidence to show that interventions aimed at providing adequate childhood nutrition or delayed childbearing prevents obstructed labor [Tsu 2009]. Using 2002 GBD data from the SEAR region, we estimated the incidence of obstructed labor (0.046822) by using data on the number of cases (n=1771) [WHO 2007a] and data on the total number of births (n =37820) [UNICEF 2004]. To account for the uncertainty in our initial estimates, we established a range of 3% - 7% for sensitivity analysis.

The initial estimate for the case fatality rate (CFR) was from a review conducted by the Disease Control Priorities Project (DCP2) [Graham 2006] who reported an average CFR of 0.7%; this estimate was adjusted according to heterogeneity in severity and underlying morbidity (e.g., severe anemia) by calibrating the model to fit multiple epidemiologic targets simultaneously. See section above on Adjustment of case fatality rates for heterogeneity in severity and co-morbidity. The implications of our adjusted CFR widened the implied plausible range. While the literature based range based on 0.007 average CFR from DCP2 was 0.005 – 0.009, the expanded plausible range based on our adjustment of a CFR of 0.019 was 0.005 – 0.025. Model projected mortality due to obstructed labor, as well as MMR, TFR, and calendar deaths for 2005, closely approximated the empiric data.

Reduction in mortality

We identified multiple studies, including two Cochrane reviews that examined that efficacy of treating obstructed labor in reducing of maternal mortality rates. A study by Yarrow et al. (2004) showed a 94.1% success rate when using vacuum-assisted deliveries. Of the nine failed vacuum deliveries, four were subsequently delivered by forceps and five by cesarean section, with no maternal mortality reported. We assumed a 95% reduction in maternal mortality when obstructed labor was managed in an appropriate facility (assisted vaginal delivery with forceps or vacuum and, if necessary, cesarean section) [Adam 2005, Johanson 2000, Hofmeyr 2000,2003; Schuitemaker 1997, Graham 2006]. To account for the uncertainty in our initial estimates, we established a range of 76% - 100% for sensitivity analysis.

Severe pre-eclampsia and eclampsia

Incidence and case fatality rate

Hypertensive disorders of pregnancy refer to a range of conditions associated with high blood pressure, proteinuria and, rarely, seizures. Severe pre-eclampsia and eclampsia have the highest case fatality rates of the hypertensive disorders of pregnancy, and can lead to placental abruption, disseminated intravascular coagulopathy (DIC), adult respiratory distress syndrome (ARDS), cerebral hemorrhage, seizures, and death. Globally, the incidence of pre-eclampsia is estimated at 3.2% of live births and eclampsia at 0.5% [AbouZahr 2004]. While in some parts of the world, such as Latin America and the Caribbean, hypertensive disorders of pregnancy are the leading causes of maternal deaths (25.7% of all maternal deaths) [Khan 2006], in India, hypertensive disorders of pregnancy rank as the fourth most common cause of maternal mortality [Dolea 2003d]. Eclampsia has a high case fatality rate, which varies among regions of the world, presumably as a function of the access to and quality of health care. [Dolea 2003d]

A retrospective study of pre-eclampsia- and eclampsia-related deaths in Chandigahr, India found that access to and delay in seeking care was a major determinant of mortality, with 37.7% in grade IV coma and 54% with recurrent convulsions prior to admission [Sawhney 2000]. The GBD 2000

reported an incidence of 0.028 and 0.008 for preeclampsia and eclampsia respectively, for the SEAR D region. [Dolea 2003d] Initial estimates for the overall incidence of hypertensive diseases of pregnancy were based on data from the 2002 edition of the WHO's Global Burden of Disease study specifically for the SEAR region. The estimate derived from this data for hypertensive diseases was higher (0.066) from the SEAR region, [WHO 2007a; UNICEF 2004] but is consistent when corrected for the proportion of hypertensive disorders that are preeclampsia and eclampsia. Given a pooled average for preeclampsia of 0.034, if 2.3% of preeclampsia in SEAR D is an approximation of eclampsia (~0.0078), then the implied incidence of hypertensive disorders is 0.069, approximating the 2002 GBD estimate.

To account for the uncertainty in our initial estimates, we established a plausible range based on our literature review, including studies in the Cochrane database. The only interventions shown to prevent pre-eclampsia are anti-platelet agents, primarily low dose aspirin, and calcium supplementation. While data from trials are insufficiently conclusive as to the optimal timing of delivery with pre-eclampsia, there is robust evidence that magnesium sulfate can prevent and control eclamptic seizures, and for pre-eclampsia, reduces the risk of eclampsia by more than 50%. [Duley 2009, Langer 2008, Tukur 2009].

The initial estimate for the case fatality rate (CFR) was from a review conducted by the Disease Control Priorities Project (DCP2) [Graham 2006] who reported an average CFR of 1.7%; this estimate was adjusted according to heterogeneity in severity and underlying morbidity (e.g., severe anemia) by calibrating the model to fit multiple epidemiologic targets simultaneously. See section above on <u>Adjustment of case fatality rates for heterogeneity in severity and co-morbidity</u>. The implications of our adjusted CFR widened the implied plausible range. While the literature based range based on 0.017 average CFR from DCP2 was 0.012 – 0.022, the expanded plausible range based on our adjustment of a CFR of 0.021 was 0.012 – 0.027. Model projected mortality due to hypertensive disorders, as well as MMR, TFR, and calendar deaths for 2005, closely approximated the empiric data.

Reduction in mortality

We assumed that severe pre-eclampsia and eclampsia required treatment with intravenous hydralazine and magnesium sulfate; in addition, approximately 10% of all cases were assumed to require emergent cesarean section [Cahuana-Hurtado 2004]. A Cochrane Review of magnesium sulphate and other anticonvulsants for women with pre-eclampsia compiled evidence from 6 trials of which the largest source of data was the Magpie Trial Collaborative Group. [Duley 2003] This review found a 59% reduction in risk of eclampsia in women with pre-eclampsia (RR 0.41) and a 46% reduction (RR 0.54) in the risk of dying in women with pre-eclampsia randomized to magnesium sulfate.[Duley 2003]. A review showed that magnesium sulphate was the better anticonvulsant choice when treating women with eclampsia, and substantial reduced the risk of further seizures when compared to diazepam [Duley 2009]. One goal of this study is whether induction of labor in women with pregnancy induced hypertension or pre-eclampsia at term reduce costs and improve quality of life as compared to expectant monitoring. Two studies looked only at mild pre-eclampsia and gestational diabetes, but not at cases of maternal or neonatal death or eclampsia [Koopmans 2007 & 2009] A study by one collaborative group found that the use of magnesium sulphate for women with pre-eclampsia was associated with a 16% reduction in the risk of death or serious morbidity related to pre-eclampsia 2 to 3 years later [Magpie Trial 2007]

We assume that aside from the use of magnesium sulphate, induction of labor could occur in facilities capable of basic and comprehensive emergency obstetric care for women who do not require emergency cesarean section. Thus we rely on the higher effect size from the Cochrane Review, although still perhaps a conservative estimate, for the reduction in the case fatality rate of

severe pre-eclampsia. We assumed severely pre-eclamptic/eclamptic women who received treatment had a 59% reduction in disease-specific mortality compared to those without treatment [Adam 2005; Graham 2006; Magpie Trial Collaborative Group 2002; Duley 2003; Crowther 2002]. A Cochrane Protocol for additional evaluation of interventions for treating pre-eclampsia and its complications has been submitted and will be useful for further updating of this estimate once the review has been published. [Duley 2009]. To account for the uncertainty in our initial estimates, and based on the literature review, we established a range of 45% - 95% for sensitivity analysis.

Pre-eclampsia and eclampsia

	Relative Risk (RR)			% Reduction in Risk		
	RR	95% CI		%	lower	upper
Hypertensive disorders of pregnancy (including eclampsia) [Graham 2006]				76%	71%	95%
Risk of eclampsia among women with pre-eclampsia [Mapgie trial 2002]				58%	40%	71%
Risk of eclampsia in women with pre-eclampsia [Duley 2003]	.41	.58	.29	59%	42%	71%
Maternal mortality among women with pre-eclampsia [Mapgie trial 2002]	.55	1.14	.26	45%	14%	74%
Maternal mortality in women with pre-eclampsia [Duley 2003]	.54	1.10	.26	46%	10%	74%
Death (potentially related to) pre-eclampsia 2-3 years after delivery [Magpie follow-up 2007]	.84	1.18	.60	16%	18%	40%
Mild pre-eclampsia [Koopmans HYPITAT trial 2007 & 2009]				64%		

Long-term morbidity

Initial estimates for complication (GBD data)	
Neurological sequelae	0.0008
Severe anemia[a]	0.090
Sheehan's syndrome	0.008
Infertility from sepsis[b]	0.086
Fistula[c]	0.022

a This estimate takes into account the rate of severe and moderate anemia, the overall incidence of PPH, and the overall incidence of severe anemia in pregnant women, during the postpartum period, and in the general reproductive age group. We assume that women with pre-existing moderate anemia contribute disproportionately to the subsequent severe anemia observed following PPH.

b Represented by the risk of PID (0.40) multiplied by the risk of infertility (0.22) given PID, to yield the estimate of 0.086

c We assume 25% are treated in Uttar Pradesh, Rajasthan, and rural India but vary this from 25% to 75% in sensitivity analysis.

Maternal deaths due to unsafe abortion

The World Health Organization (WHO) defines "unsafe abortion" as "the termination of an unintended pregnancy either by persons lacking the necessary skills or in an environment lacking the minimal medical standards, or both" [World Health Organization 1998, Ahman 2004]. In India,

unsafe abortion is responsible for up to 10% of all maternal deaths [Registrar General 2006]. Most abortions occur in married women with more than 2 children and who lack access to short- or long-term contraception [Ganatra 2002,2006; Mills 2007]. In one review of admissions for unsafe abortion to a tertiary care center in North India [Jain 2004] over a 15-year period (1988-2002) unsafe abortion caused ~17% of maternal mortality in the hospital. Initial estimates for the probability of an elective abortion were drawn from a published study that reported an abortion ratio of 17% (defined as the number of abortions per known pregnancies, including miscarriages) and studies that allowed for an approximate estimation of the proportion of elective abortion that is illegal or unsafe in South-central Asia [Henshaw 1999; Shah 2004; Sedgh 2007]. We used other published data on hospitalizations for abortion-related morbidity and mortality to check the face validity of estimates, and establish a plausible range for sensitivity analysis. [Jain 2004; Singh 2006; Mills 2007; Coyaji 2002] These estimates were varied from 10% to 22% (abortion ratio), and from 25% to 50% (proportion of safe abortions), respectively. [Ahman 2004, Berer 2004, AGI 2006,2007; Lule 2007] Calibration exercises leveraged the information on distribution of direct causes of maternal mortality, including unsafe abortion, to check the face validity of estimates. [Registrar General 2006; Khan 2006]

We used Asia-specific estimates from the WHO, and assumed that illegal/unsafe abortion is associated with a mortality of 300 per 100,000 procedures. [Ahman 2004,2007] We developed an initial estimate of infertility from illegal/unsafe abortion of 12%, derived from the GBD study, in which the number of cases of infertility arising from unsafe abortion projected for India was divided by the number of unsafe abortion procedures projected for India [Murray 1998]. This was refined using more recent data, and a plausible range was established for the risk of infertility using higher and lower risk estimates reported for other world regions by the WHO [Ahman 2006]. In addition, we assumed a proportion of safe and unsafe abortion, 2.8% and 14.7% respectively, was associated with post-abortion complications requiring hospitalization and incurring quality of life decrements and costs [Johns 2007, Singh 2006].

The Indian Parliament passed the Medical Termination of Pregnancy (MTP) Act in 1971, which stated that abortion can be performed under the following conditions: “to save the woman's life, to preserve physical health, to preserve mental health, rape or incest, fetal impairment, economic or social reasons, and contraceptive failure.” [Mills 2007] However, in most states services have been available in less than one-fifth of primary health care centres [Ramachandar 2002, 2004, 2005]. There are regional as well as rural-urban disparities in access to abortion services. [Ganatra 2006; Ipas, 2008] Barriers include untrained providers (and lack of availability and acceptability of trained non-physician providers), lack of equipment, cultural stigma, and lack of knowledge in women. [Hirve 2004**,** Shah 2005, Ganatra 2002] To provide broader access, a recent amendment to the original law in India decentralizes the approval of locations designated as MTP Centers from the state to the district level. As part of the Reproductive and Child Health program (RCH II), included within the National Rural Health Mission (NRHM 2005-2012) [Government of India 2005a,b,c], there is a commitment to expand MTP facilities to make safe abortion services accessible to all women, particularly to those in the rural areas.

To estimate the risk of mortality from safe abortion the majority of our estimates were drawn from U.S. data in the early 1970s, a period in which elective first-trimester abortion was legalized in most U.S. states. Data from the Joint Program for the Study of Abortion (JPSA) demonstrate manual vacuum aspiration (MVA) is associated with fewer total and major complications compared with dilation and curettage (D&C). [Tietze 1971,1972], consistent with results from other US large, prospective studies [Edelman 1974] and smaller scale studies in developing countries [Laufe 1977]. By synthesizing British data for 439,400 legal, first-trimester abortions performed between 1968-1973 and U.S. abortion surveillance data from 1972-1975, we estimated the mortality risk for D&C was 1.8 per 100,000 procedures and MVA was 1.3 per 100,000 procedures [IOM 1975,

Koonin 1993]. Our estimated mortality risk associated with MVA falls within the range of values (1.0 to 1.6 per 100,000 procedure) reported in published literature from the 1970s [Tietze 1971,1972; IOM 1975]; however, we acknowledge the actual risk may be higher in developing countries [Ahman 2007].

For sensitivity analyses on the complications and costs of medical safe abortion, we used data on vaginal misoprostol [Faundes 2007, Shannon 2004]. We conservatively assumed a success rate of 80% for the vaginal misoprostol regimen, which falls roughly midway between reported estimates [Faundes et al. [2007] reported a success rate of 65%-93% for regimens using 800 mcg vaginal misoprostol (1 to 5 doses); Carbonell et al. [1998] found a success rate of >90% with 2400 mcg of misoprostol or in very early pregnancies terminated up to 9 weeks gestational age, compared to <90% in those terminated in the late first-trimester.] Major complications resulting from medical abortion include pelvic infection and hemorrhage necessitating transfusion and were estimated to arise in 0.75% of all procedures [Faundes 2007, Shannon 2004; Creinin 2006; Grimes 2005; Reeves 2006].

Subsection B

Data: Coverage Inputs and Selected Services

Coverage rates of skilled birth attendants and traditional birth assistants as well as of facility-based births were derived from national databases and published literature. [IIPS 2007 chapter 8] Using data from NFHS-3, more than half of births occur at home, as displayed below. [IIPS 2007] By birthing assistant, just under half of births are delivered by a skilled provider, with a major difference between states, and between rural and urban areas [IIPS 2007]. In the model delivery setting is differentiated by site including (1) home; (2) birthing center or health centre (used interchangeable here), (3) facility with bEmOC, (4) facility with cEmOC, and differentiated by health provider including (1) family member, (2) traditional birth attendant [TBA], (3) skilled birth attendant. Facilities classified as birthing centres or health centres are assumed to be staffed by SBA with expectant management of labor but do not have all signal functions to qualify as bEmOC.

Selected Model Parameters (NHFS-3)[a] [IIPS 2007]

Variable	Baseline Value (%)					
	India		Uttar Pradesh		Rajasthan	
**Delivery location**						
Total skilled delivery	48.3		29.2		41.0	
% begun in facilities	40.7		22.0		29.6	
% home delivery (SBA)[b]	12.8		9.2		16.2	
	Urban	Rural	Urban	Rural	Urban	Rural
**Delivery location**						
Total skilled delivery	75.2	39.1	50.5	23.8	74.2	32.5
% begun in facilities	69.4	31.1	39.9	17.5	63.7	20.8
% home delivery (SBA)[b]	19.0	11.6	17.6	7.6	28.9	14.8

a SBA: skilled birth attendant

b We calculated the percentage of births with skilled attendance at home by subtracting the percentage delivered in facilities (which we assume are with skilled attendance) from the total of births with skilled attendance (total skilled delivery – facility based births)/home births (0.391-0.311) / (1-0.311) = 0.116 or 11.6%.

India: state-specific data (National Family Health Survey-3)[a] [IIPS 2007]

	**% received all recommended types of antenatal care**	**% of births delivered in health facility**	**% of deliveries assisted by health personnel**
**North**			
Rajasthan	8.6	29.6	41.0
**Central**			
Uttar Pradesh	4.1	20.6	27.2
**East**			
Bihar	5.8	19.9	29.3
**West**			
Maharashtra	21.6	64.6	68.7
**South**			
Andhra Pradesh	28.2	64.4	74.9

a Based on births within the last five years

Extrapolating from data on the availability of emergency obstetric services and the relative availability of bEmOC to cEmOC, we estimated for the status quo, approximately 30% of facility-based births occur in an EmOC-capable site, of which 17% offer cEmOC [AMDD 2002, IIPS 2007]. We therefore assume in the base case analysis that for births that occur in a facility with EmOC capacity, approximately 90% are assumed to occur in bEmOC facilities and 10% in cEmOC facilities. In strategies that shift home births to facilities, additional analyses are conducted using several alternative assumptions. For example, as shown below, we explore in a scenario analysis the impact of (a) changing the distribution of routine deliveries that occur in primary facilities lacking EmOC (birthing centres/health centres) and facilities with EmOC, and (b) changing the distribution of deliveries in EmOC that occur in bEmOC versus cEmOC.

Sensitivity Analyses

Distribution among facilities (rural India)

	Base Case		Analysis 1		Analysis 2		Analysis 3	
	A*	B*	A*	B*	A*	B*	A*	B*
Total facility births	Vary 30-80%		Vary 30-80%		Vary 30-80%		Vary 30-80%%	
Begin HC/BC**	70%	70%	50%	50%	25%	25%	0%	0%
Begin bEmOC**	27%	3%	45%	5%	67.5%	7.5%	90%	10%
Begin cEmOC**	3%	27%	5%	45%	7.5%	67.5%	10%	90%

* A = 90% bEmOC, 10% cEmOC; B = 10% bEmOC, 90% cEmOC

** HC = health centre, BC = birthing centre. bEmOC = basic emergency obstetric care, cEmOC = comprehensive emergency obstetric care. HC and BC used interchangeably and assume SBA, clean delivery, expectant management, but lack all 6 signal functions. In this particular analysis we also assume SBA-administered misoprostol in birthing centres/health centres.

Antenatal care

Data on antenatal care (ANC) from the NFHS- 3 were used for coverage rates for the national model, and were stratified by urban and rural status when available. State-specific data were used for Uttar Pradesh, and were also stratified by rural and urban status when possible. [IIPS 2007 Chapter 8] We assumed in our analyses that antenatal care includes 4 visits, tetanus vaccination, syphilis, gonorrhea, chlamydia screening (and treatment), urinalysis, blood tests, treatment for anemia, counseling (e.g., family planning, spacing, intrapartum care).

National and state-specific ANC and anemia treatment rates (NFHS-3) [IIPS 2007]

	% had 3 or more ANC visits	% with an ANC visit in the 1st trimester of pregnancy	% received info about specific pregnancy complications	% given or bought IFA	% took IFA for at least 90 days
India	52	43.9	36	65.1	23.1
Rajasthan	41.2	34	29.8	57.7	13.1
Uttar Pradesh	26.6	25.7	14.2	53.2	8.8
Bihar	17	18.7	15.8	29.7	9.7
Kerala	93.6	91.9	72.9	96.4	75.1

**Variable (NHFS-3)** [IIPS 2007]	**Baseline Value (%)**					
	**India**		**Uttar Pradesh**		**Rajasthan**	
Prenatal care	50.7		26.3		41.2	
Treatment for anemia	22.3		8.7		13.1	
	**Urban**	**Rural**	**Urban**	**Rural**	**Urban**	**Rural**
Prenatal care	73.8	42.8	40.9	22.6	74.7	31.7
Treatment for anemia	34.5	18.1	16.4	6.7	30.2	8.3

In addition, Ram and Singh [2006], based on data from the District Level Household Surveys, found that utilizing antenatal care services may lead to the utilization of other maternal health services such as institutional delivery, delivery with skilled attendance, and advice-seeking behavior for pregnancy-related complications and postpartum complications. Sensitivity analyses were conducted to explore the range of potential benefits associated with the scenarios that antenatal care increases facility over home births and the use of emergency care for those remaining at home.

Family planning

Our choice of including a comprehensive strategy of enhanced family planning to reduce the unmet need for contraception, for purposes of *both* limiting and spacing, reflects the overarching goal of the Government of India to address the unmet need for contraception and bring the total fertility rate down to replacement level by 2010 (IIPS 2007). The effectiveness of family planning is incorporated into the model as a set of variables that reflect (i) Coverage level of contraceptive method; (ii) Distribution of contraceptive type; (iii) Type-specific failure rate. We use state-level and setting-specific data to represent the current met need for contraception and the distribution of methods used by age. Failure rates are conditional on the method used.

Coverage level of contraceptive method

Estimates relating to unmet need for family planning are from the National Family Health Survey 2005-2006 (NFHS-3).Approximately 13 percent of currently married women in India have an unmet need for family planning, with limiting (7%) slightly higher than spacing (6%) using average national estimates. Unmet need declines with age, from 27% (ages 15-19) to 2% (ages 45-49). Women younger than ages 15-24 have a greater unmet need for spacing than for limiting. In contrast, the unmet need for spacing decreases from age 15-19 to age 35-39. Women in rural India have a higher unmet need for spacing as well as limiting relative to their urban counterparts. (IIPS 2007, ch 5, p.160). In our base case analysis both age-patterns and rural/urban status are incorporated, but we do not stratify according to education and wealth. We do explore age-specific focused interventions in sensitivity analyses, specifically, focusing efforts to increase modern contraception in younger women. The motivation for these exploratory analyses was based on data showing that although over 40% of women use modern contraception, family planning is used mainly for the purpose of providing long-term contraception in India (IIPS 2007). This is reflected in the low contraceptive prevalence in younger females and the overwhelming domination of the contraceptive method mix by sterilization. For example, only 5% of married women ages 15-19 years use modern contraception versus 67% of women ages 35-39 years. Female sterilization is the most common method of contraception, accounting for more than 75% of total contraceptive use. Women below age 25 are more likely to have used modern and traditional spacing methods, whereas women age 25 and over are more likely to have undergone sterilization. In fact the median age for women undergoing sterilization is 26 years, illustrating the typical childbearing pattern of women in India (Lule 2007).

Distribution of contraceptive type

Selected model parameters, NHFS-3[a] [IIPS 2007]

Variable (NHFS-3)	Baseline Value (%)					
	India		Uttar Pradesh		Rajasthan	
**Family planning**						
Any method	56.3		43.6		47.2	
Modern methods	48.5		29.3		44.4	
Pill	6.4		5.8		4.5	
IUD	3.7		4.8		3.6	
TOL	76.9		59.0		77.0	
Condom	10.9		29.7		12.8	
	**Urban**	**Rural**	**Urban**	**Rural**	**Urban**	**Rural**
**Family planning**						
Any method	64.0	53.0	56.3	39.7	65.7	40.5
Modern methods	55.8	45.3	42.3	25.2	62.0	38.0
Pill	7.0	6.2	7.6	5.2	7.9	2.6
IUD	6.1	2.4	7.6	3.2	3.9	3.4
TOL	67.7	81.9	44.2	66.7	63.9	84.7
Condom	17.9	7.3	39.2	24.6	21.0	8.2

a IUD: intrauterine device; TOL: female sterilization

Contraception prevalence rate is 56 percent (IIPS 2007, ch5, p.120) and higher in urban than rural areas. Overall, 86% of those using contraception use modern methods and 14% use traditional methods. Female sterilization accounts for 77% of modern methods used, and the prevalence is similar in urban and rural women. Condoms and rhythm method are used commonly for spacing, and modern spacing methods (pill, IUD, condom) are higher in urban areas compared with rural

areas. Condom use is 3-fold higher in urban areas. Among sexually active unmarried women age 15-49, 36 percent report using a modern method. Women younger than age 25 are more likely to have used modern and traditional spacing methods, whereas women age 25 and over were more likely to have undergone sterilization.

Type-specific failure rate. We used contraceptive failure rate estimates from the UNFPA's Reproductive Health Costing Tool (UNFPA 2007, Trussell 1990). Failure rates by method: IUD (4%), oral contraceptives (8%), and condoms (19%), injectables (2.9%), female sterilization 0.5%), and male sterilization (0.2%).

Overview of our strategies to increase contraceptive use

We elected to use unmet need as our main intervention target to describe the influence of increasing access and uptake of modern contraception, as it reflects two groups of women: (a) women who are not using any method of contraception but who do not want any more children - unmet need for *limiting* and (b) those who are not using contraception but want to wait two or more years before having another child - unmet need for *spacing*. [The sum of the unmet need for limiting and the unmet need for spacing is the modeled unmet need for family planning]. For India as a whole, there has been a decrease in the unmet need for family planning from 16 percent in NFHS-2 to 13 percent in NFHS-3. The decrease in the unmet need for spacing was higher than the decrease in the unmet need for limiting. NFHS-3 showed that in most states the unmet need for limiting is higher than that for spacing. The table below illustrates differences in baseline model assumptions about access to family planning (e.g., the unmet need for spacing and limiting births) and the magnitude of stepwise increases characterizing different strategies.

Modeling increases in use of family planning

	Status quo	25% unmet need	50% unmet need	75% unmet need	100% unmet need	total % unmet need
Urban India	64.0%	66.5%	69.0%	71.5%	74.0%	10.0%
Rural India	53.0%	56.7%	60.3%	64.0%	67.6%	14.6%
Urban Uttar Pradesh	56.3%	60.2%	64.1%	67.9%	71.8%	15.5%
Rural Uttar Pradesh	39.7%	45.7%	51.6%	57.6%	63.5%	23.8%
Rajasthan	47.2%	50.9%	54.5%	58.2%	61.8%	14.6%
Urban Rajasthan	65.7%	68.2%	70.6%	73.1%	75.5%	9.8%
Rural Rajasthan	40.5%	44.6%	48.7%	52.7%	56.8%	16.3%

Interventions in the model referred to as "enhanced family planning" in the manuscript refer to total unmet need, inclusive of both limiting and spacing.

	Included in modeling the status quo [a,b] (base case)	Included in the enhanced family planning strategy (base case)
Unmet need for spacing	Yes	yes
Unmet need for limiting	Yes	yes
Contraceptive failure [c]	Yes	indirectly

[a] stratified by rural and urban status, age-specific

Other questions in the National Family Health Survey 2005-2006 (NFHS-3) for which data are available that were not directly used in the model include (1) knowledge about contraception; (2) ever use of contraception (measure of the cumulative experience of a population with family planning); (3) stage of family-building at time of contraceptive uptake and method choice; (4) intention to use a method of contraception in the future; (5) source of contraception by type (e.g., public sector, private sector) and location of sterilization (e.g., government/municipal hospitals, community health centers). If data on interventions that leverage this information becomes available, it could be integrated in future analyses.

Relationship between abortion and contraception

The risk of unsafe abortion is reduced through the use of contraception, legalisation of elective abortion, and the use of safe abortion methods by a high-quality and trained provider. [AGI 2007] Access to safe, effective contraception can substantially reduce the need for abortion to regulate fertility. [Mills 2007, Lule 2007] Mills et al. [2007] report 64.9% of women in Uttar Pradesh sought elective abortion because of an unwanted pregnancy whereas less than 15% sought abortion because of complications during pregnancy or illness. We therefore explored correlations of 25% to 75% in the model. The model is used to generate the reduction in unsafe-abortion related deaths due to increased access to and use of family planning (modern methods), in addition to the averted deaths due to safer abortion and postabortion services.

Postpartum care

In the recent NFHS-3, women reported complications two months after their most recent deliveries, including massive vaginal bleeding for 12% of births and a very high fever for 14% of births; both complications were more common among rural than urban mothers. [IIPS 2007 chapter 8] Bang et al. [2004] document the high incidence of maternal morbidity during labour and puerperium in rural homes in Gadchiroli, India, with more than 40% of women experiencing postpartum morbidity. While it was difficult to find quantitative data on reduction in mortality with postpartum care, we did conduct sensitivity analysis to estimate the potential averted morbidity and costs. In addition, we explored the potential benefits associated with increased use of contraception and other reproductive health services associated with postpartum care.

Maternal care indicators by state, India (NFHS-3) [IIPS 2007]

	% deliveries w/ postnatal check-up[a]	% deliveries w/ postnatal check-up w/in 2 days
India	41.2	37.3
Rajasthan	31.8	28.9
Uttar Pradesh	14.9	13.3
Bihar	17.8	15.9
Andhra Pradesh	73.3	64.1

a Based on the last birth within the five years preceding the survey. Postnatal check-ups are defined as checks on the woman's health within 42 days of the birth

Impact of community-based interventions

The Lancet series on maternal survival suggested that the evidence in support of community-based interventions, especially those geared towards reducing maternal mortality (such as the use of misoprostol by TBAs to reduce PPH in the home setting or clean birth kits in reducing death from sepsis in the home), is weak. [Campbell 2006]. A Cochrane Review of the evidence on the effects of TBA training for improving health behaviors and pregnancy outcomes found insufficient data to document any association between training and maternal mortality. [Sibley 2007] Through a review

of the recent literature, we identified several trials that investigated the use of community-based approaches to improve maternal health, most involving the use of misoprostol to reduce mortality from PPH. Sanghvi et al. (2004) reported that community volunteers were able to successfully distribute oral misoprostol tablets to women and encourage the acceptance and use of the tablets; women in the intervention area were 45% less likely to need an emergency referral for PPH. Prata et al. (2005) also found positive results from training TBAs to diagnose and treat PPH with misoprostol, demonstrated through lower referral rates for women with PPH in the intervention area than for women in the control area (2% versus 19%). In an extensive review of the literature on interventions suitable for resource-poor settings, Prata et al. (2009) conclude that even TBAs are able to provide some basic maternal care including misoprostol for PPH. Walraven et al. (2005) investigated the use of misoprostol versus placebo for management of the third stage of labor for home births under guidance of trained TBAs but found a non-significant reduction in severe PPH. In rural India, a randomized controlled trial of the use of oral misoprostol to prevent PPH in a home birth setting found an 80% reduction in the rate of acute severe PPH in women given misoprostol as opposed to expectant management of the third stage of labor [Derman 2006]. Recognizing that the evidence is still quite limited, a Cochrane Protocol has recently been submitted to assess the effectiveness of community-based intervention packages for preventing maternal mortality and morbidity. [Haider 2009]

An analysis in which we simulate the community-based interventions included in Prata et al. (2009) has been included in the Results Section of this document.

Coverage of community-based interventions in India

Aside from the need for convincing evidence on the effectiveness of community-based interventions to improve maternal health, additional evidence on the potential reach of such interventions across a large portion of the population is required to justify policy relevance of these strategies. Prior studies in India have found conflicting results on the ability to achieve high coverage levels of community-based interventions. [Bang 2005a, 2005b, Baqui 2008, Patel 2010] Bang et al. [2005a, 2005b] achieved high coverage in using trained village health workers (VHWs) to provide home-based neonatal care in a rural part of India; home-based care was provided to 93% of neonates. [Bang 2005a] Encouraging VHWs to be present during home delivery with a small ($1.00) financial incentive resulted in VHWs attending 84% of home deliveries. [Bang 2005b] However, most community-based trials are conducted under controlled conditions, thereby ensuring high program coverage. [Baqui 2008]; Methods to achieve scale-up are needed. [Bang 2005a] Baqui et al. (2008) evaluated the effect of a community-based package of maternal and newborn interventions implemented at scale using existing government infrastructure. While community-based workers were present throughout the study area, 38% of women who had recently delivered had not received any home visits (antenatal or postnatal). Patel et al. (2010) concluded that while there is potential for community health workers to deliver interventions on a large scale, their effectiveness will be limited by a weak rural health system, lack of incentives, absenteeism, and lack of support and supervision. Singh et al. (2009) investigated the potential of a public-private partnership scheme in which the government of Gujurat paid private obstetricians practicing in rural areas to provide free delivery care to poor women. Out of the payment per delivery, obstetricians were required to pay the woman giving birth for transport to reduce the delay in seeking delivery care, and the person who accompanied the woman. This scheme was found to be very successful during the trial period, in that numerous obstetricians have joined the scheme, and the estimated coverage of deliveries among poor women increased from 27% to 53%. [Singh 2009]

Data and Assumptions: Barriers to Effective Referral

Barriers to Effective Referral to EmOC

Effective referral relies on the ability to overcome three critical delays (a) recognition of referral need and willingness to be referred (by provider and delivery location); (b) expedient transfer to referral facility (determined by distance, affordability, available transport); and (c) timely treatment in an appropriate facility capable of high-quality emergency obstetrical care (e.g., 6 signal functions in bEmOC, blood transfusion and surgery in cEmOC). We expanded Thaddeus and Maine's [1994] "three delays" framework to reflect the multidimensional nature of each of these delays and the heterogeneity between and within countries as to which delays and components are most critical. A successful referral in our model incorporates a series of elements, each of which could act as a barrier to the care a woman with pregnancy-related complications requires.

Delay Category 1. Recognition of need for referral and/or willingness to be referred

We include in this category both failure or delay in recognition of the need for referral by the SBA as well as delay in recognition for need of referral or unwillingness to be referred on the part of the woman or her family. We assumed the recognition rate for complications developing during home deliveries would vary based on the level and skill of the birth attendant. Based on data from Honduras regarding traditional (untrained) birth attendants, [Danel 2003] we assumed an 11.5% recognition rate for unskilled delivery at home, and a 20% recognition rate for skilled birth attendants at home. [Graham 2006] Based on literature and India-specific data, we established a plausible range for sensitivity analysis. [National Family Health Surveys (NFHS-3 [IIPS 2007, IIPS 2007 Chapter 8]), District Level Household Survey (DLHS [IIPS 2006]), Facility Survey [IIPS 2005], state-level facility surveys (IIPS 2007-08 Uttar Pradesh [IIPS Fact Sheet Uttar Pradesh]; IIPS 2007-08 Rajasthan [IIPS Fact Sheet Rajasthan]); government reports (Government of India 2008a); published studies (Koblinsky 2006; Vora 2009; Iyengar 2009a,b,c; Mavalankar 2009; Mills 2007)]

We assumed life-threatening complications (those needing cEmOC capability) occurring at bEmOC were recognized as needing transfer to a facility with cEmOC. We also included an analysis assessing the impact of delays in facility transfers (i.e., incorporating the delay due to transport problems, logistics, or fees). In addition, we assumed an “erroneous” referral rate (in the absence of complications), owing to misdiagnosis and lack of patient monitoring support, that varied from 2.5% to 10% based on location of delivery and skill level of birth attendant.

A literature review on the persistence of poor maternal health in Rajasthan [Iyengar 2009a] revealed that although 73% of women had contacts with health professionals during pregnancy, less than one-sixth of women received advice about danger signs or place of delivery. Gupta et al. [2006] reported on maternal mortality in Uttar Pradesh, finding that almost 2/3 of the 286 maternal deaths occurred before reaching the health facility; about half occurred at home and another 12.6% of the cases died during transit. More than one-third of women were delayed for referral because they were not considered serious enough; an additional 32% were delayed at home because the woman refused referral (10%), money could not be arranged (16%) or there was no one to take care of family at home (6%). Mills et al. [2007] reported in an analysis of 45 maternal deaths in Uttar Pradesh with delays in obtaining appropriate care, that the decision to seek care took more than a day in nearly half of the cases. In some instances the initial decision was to first seek care from a non-health professional; by the time a woman finally reached the appropriate health facility, it was too late or the woman ended up dying en route. Finally, in Andhra Pradesh, Prakasamma [2009] found that women did not go to primary health centers (PHCs) for childbirth since there were no service providers or facilities; results of community studies indicated the unwillingness of people to use these facilities for maternal emergencies. Of the 148 deaths in the study, 20.3% died on way to the hospital, and 13.5% died at home, indicating a need for earlier recognition and willingness to be referred. The Tables below show the initial *range* of baseline estimates used in sensitivity analyses, and the initial *range* across which stepwise improvements were made in the temporal strategies evaluated. These ranges were expanded in a series of exploratory analyses.

Range of baseline estimates for status quo used in sensitivity analysis					
	India	India, Urban	India, Rural	UP, Urban	UP, Rural
Recognition of referral need					
Unskilled at home	8%-20%	8%-20%	8%-20%	8%-20%	8%-20%
Skilled at home	15%-30%	15%-30%	15%-30%	15%-30%	15%-30%
Skilled at birthing centre[a]	30%-60%	30%-60%	30%-60%	20%-40%	20%-40%

a birthing centre not considered EmOC; we consider all attendants at EmOC to be skilled

Upper bound of stepwise increases in recognition of need/willingness for referral					
	India	India, Urban	India, Rural	UP, Urban	UP, Rural
Recognition of referral need					
Unskilled at home	8%-30%	8%-30%	8%-30%	8%-30%	8%-30%
Skilled at home	20%-90%	20%-90%	20%-90%	15%-90%	15%-90%
Skilled at birthing centre[a]	40%-95%	40%-95%	40%-95%	40%-95%	40%-95%

a birthing centre not considered EmOC; we consider all attendants at EmOC to be skilled

Delay Category 2. Expedient transfer from birth location to facility

We include in this category availability of timely and affordable transportation from birthing location to facility, functioning vehicle with fuel, and if necessary, provision of interim lifesaving care en route. The availability of transport is assumed to be a function of infrastructure (ambulances, neighborhood emergency transport networks, road densities, distance to hospitals, etc.). The World Bank [2002] reports that 70% of India's population is rural, and only 61% of the rural population has access to an all-season road. [PMGSY 2006] Although the rural road network is extensive, many roads are impassable in bad weather. About 40% of villages do not have access to all-weather roads to market centers and main road networks; the problem is worse in India's northern and northeastern states. [PMGSY 2006, Government of India 2008a,2009].

We made assumptions about effective transfer that varied by both delivery location and urban/rural setting, and were intended to reflect access to transport, reliable fuel and accompanying person en route, and interim care if necessary. [Government of India 2008a,2009; IIPS 2005,2006,Fact Sheet: Uttar Pradesh Fact Sheet: Rajasthan; Vora 2009; Padmanaban 2009; Iyengar 2009a] This ranged from a high of 81% in urban India to a low of 24.4% when deliveries began at home in rural India. We assigned 81% (and not 100%) as the baseline rate of "expedient accurate referral" in an urban setting lower-level facility, to reflect delays attributable to multiple transfers between facilities, and delays related to being turned away from one hospital and having to travel to another,.

Based on other literature, and India-specific studies, we established a plausible range for sensitivity analysis. A study in Uttar Pradesh, Karnataka, Uttranchal, Maharashtra and Delhi reported 55.6% of women did not seek treatment because of a transportation barrier. In the maternal deaths evaluated, approximately 25% occurred at home in the absence of referral and 15% en route. [Pandey 2003] Studies in the Indian states of Andhra Pradesh, Maharashtra, [Ganatra 1998] and Rajasthan found that 42% to 52% of maternal deaths occurred at home or in transit to a hospital. [Mavalankar 2005] In a qualitative assessment in Uttar Pradesh, [Mills 2007] nearly 19% of deaths occurred en route to a health facility. A survey of maternal deaths from seven districts in Uttar Pradesh found that nearly one quarter of families were unable to arrange transport, and 16% did not have enough money to pay for the transport, summing to 41% [Gupta

2006]. According to this study, while 50% of maternal deaths occurred at home, 12.6% of women died while in transit to a facility.

A study in 3 states in India [Murthy 2004] concluded that ~62% of deaths and 41% of cases with complications experienced multiple referrals (usually first to a primary health center, which was inappropriate for 60% of cases resulting in deaths; primary health centers then referred women further leading to a loss of 3-4 hours of critical time). Distances to referral locations were much greater in cases of deaths, and took significantly longer to reach these locations (18% reached within 1 hour). While the majority (90%) of district hospitals in India had an emergency vehicle available for transfers, a facility survey conducted in 2003 found that over 40% of first referral units and over 75% of primary health centres and subcentres had no functional vehicle on site on the day of the survey. [India Facility Survey, Phase II, 2003] In high-MMR states, women face even greater difficulty in reaching referral locations because of distance, poor roads, and lack of transport; for example, in Uttar Pradesh and Rajasthan, 82% and 90% of primary health centres had no functional vehicle respectively, as compared with 77% nationally. [India Facility Survey, Phase II, 2003] Primary and secondary facilities in these provinces also fared substantially worse than the national average in terms of availability of a telephone for emergency communication purposes. [India Facility Survey, Phase II, 2003] A study in Uttar Pradesh found that more than half of women who were referred from both government and private facilities took more than 1 hour to reach the place of treatment [Mills 2007].

Other country-specific reports document the heterogeneity in transport services for referral [Government of India 2008a,2009; IIPS 2005,2006,Fact Sheet: Uttar Pradesh,Fact Sheet: Rajasthan, Vora 2009; Padmanaban 2009, Iyengar 2009] In a case study in Rajasthan, Iyengar et al. [2009a] report on referral and transport from home and from facilities, finding that despite financial incentives and assistance for transport, often women did not use these funds. Among the reasons provided were that a functional vehicle and telephone were not available in half of the facilities, severely limiting the ability to provide prompt referral.

The Tables below show the initial *range* of baseline estimates used in sensitivity analyses, and the initial *range* across which stepwise improvements were made in the temporal strategies evaluated. These ranges were expanded in a series of exploratory analyses.

Range of baseline assumptions for status quo used in sensitivity analysis (ability to transfer to appropriate facility expediently, reflecting transport, supportive care en route, no delays)

	India		Uttar Pradesh		Rajasthan	
From home	20%-40%		20%-40%		20%-40%	
From birthing centre	40%-65%		30%-50%		30%-55%	
From bEmOC[a]	55%-80%		40%-60%		50%-70%	
	Urban	Rural	Urban	Rural	Urban	Rural
From home	35%-55%	15%-35%	30%-50%	15%-30%	30%-50%	15%-30%
From birthing centre	60%-80%	40%-60%	45%-65%	25%-45%	50%-70%	35%-55%
From bEmOC[a]	70%-90%	50%-70%	55%-75%	35%-55%	60%-80%	45%-65%

a in the situation where cEmOC would be necessary for lifesaving transfusion, surgery, or management of shock

Upper bound of stepwise increases in ability to transfer to appropriate facility expediently (reflecting transport, supportive care en route, no delays)

	India		Uttar Pradesh		Rajasthan	
From home	30.4%-85%		22.3%-75%		24.4%-85%	
From birthing centre	54.8%-90%		40.3%-90%		48.8%-90%	
From bEmOC[a]	67%-95%		49.3%-95%		61%-95%	
	Urban	Rural	Urban	Rural	Urban	Rural
From home	44%-85%	24%-75%	38%-85%	18%-75%	40%-85%	20%-75%
From birthing centre	69%-95%	49%-90%	56%-90%	36%-90%	58%-90%	46%-90%
From bEmOC[a]	81%-98%	61%-95%	65%-95%	45%-95%	71%-95%	57%-95%

a in the situation where cEmOC would be necessary for transfusion, surgery, or management of shock

Delay Category 3. Availability and quality of services at EmOC facilities

We include in this category availability and quality of services at EmOC facilities, including the presence of a facility open 24 hours per day with adequate staffing and supplies, expedient attention (e.g., without delay to collect fees or requirement for family to bring supplies), and care that is evidence-based and of high-quality. Assumptions for this category are challenging as even in locations where there might be adequate numbers of doctors, or an adequate number of facilities, attributes such as round-the-clock availability, expedient care without delay, adequate supplies, and high-quality practice are critically influential on the effectiveness of health service delivery. The facility categories are flexibly modeled such that particularities of the public health infrastructure [IIPS 2005; Government of India 2008a] in different settings (country, state, rural versus urban areas) can be accurately represented in terms capacity and cost. Facility levels (India Facility Survey Phase-II [2003]) are categorized as (1) *primary-level facilities,* which may not have all bEmOC functions but could function as birthing centres with SBA staffing, 24-hour intrapartum care, and reliable referral connections (e.g., subcentre, primary health centre [PHC]); (2) *secondary facilities* with bEmOC capacity (e.g., first referral unit [FRU], community health centre [CHC]; and (3) *tertiary facilities* with cEmOC capacity (e.g., district hospital, some first referral units) [India Facility Survey Phase II, 2003].

Framework to differentiate facilities

Health facilities in India*	Level	Staff	Model Category	Model Assumptions
District hospital	3rd	Specialists, Ob/Gyn	**cEmOC**	**Specialists, obstetricians Active-management of labor, availability blood transfusion, surgery (e.g., c-section), intensive hemodynamic support**
First referral unit	2nd	Med Officers, Specialists, Ob/Gyn	**bEmOC**	**SBA active-management of labor**
Community health centre	2nd	Med Officers, Specialists, Ob/Gyn		
Primary health centre	1st	Med Officers, Staff Nurse	**Health centre (HC) or birthing centre (BC)**	**SBA expectant-management of labor**
Subcentre	1st	Female and Male Health Workers		

* Public-health facilities in India as categorized in the India Facility Survey Phase-II [2003]

Delivery setting is differentiated by site including (1) home; (2) birthing center or health centre (used interchangeable here), (3) facility with bEmOC, (4) facility with cEmOC, and differentiated by health provider including (1) family member, (2) traditional birth attendant [TBA], (3) skilled birth attendant. Facilities classified as birthing centres or health centres are assumed to be staffed by SBA with expectant management of labor but do not have all signal functions to qualify as bEmOC. Facilities with basic EmOC (bEmOC) are assumed to be capable of administering injectable antibiotics, oxytocics, and sedatives or anti-convulsants, performing manual removal of placenta, removal of retained products, and assisted vaginal delivery. Facilities with comprehensive EmOC (cEmOC) also are able to provide blood transfusion, cesarean section, and management of advanced shock.

We recognize that some tertiary sites will not have a blood bank and some secondary sites may eventually be able to perform c-section; further, we recognize that in the strategies that include stepwise investments in infrastructure and facility improvements, not all facilities will be expected to be fully implemented as one of the three distinct types. However, because the costs, functions and staffing are fairly closely aligned with basic or comprehensive EmOC capacity, this simple categorization captured the most important dimensions for purpose of this analysis. Above is a stylized example of how public health facilities in India, as categorized in the India Facility Survey Phase-II [2003], may be superimposed on our general model framework.

Our base case estimates on the availability and quality of EmOC services range from a high of 67.5% in urban India to a low of 26.4% in rural Uttar Pradesh. These assumptions were based on survey data [e.g., National Family Health Surveys (NFHS-3 [IIPS 2007]), District Level Household Survey (DLHS) [IIPS 2006] and Facility Survey [IIPS 2005], state-level facility surveys (IIPS 2007-08 Uttar Pradesh [IIPS Fact Sheet: Uttar Pradesh], IIPS 2007-08 Rajasthan [IIPS Fact Sheet: Rajasthan]), government reports [Government of India 2008a], and published studies [Vora 2009; Iyengar 2009a,b,c; Mavalankar 2009; Mills 2007].

As shown below, more than 55% of subcentres and 30% of primary health centres do not have electricity; over 70% of level 2 and 50% of level 3 facilities do not have a separate operation theatre for gynaecological purposes; more than 50% of all level facilities do not have a separate, aseptic labor room; over 70% of the first referral units and 80% of community health centres do not have stable linkages with a district blood-bank; more than half of the CHCs, FRUs, and district hospitals do not have residential quarters for medical officers, with obvious implications for lack of 24-hour care. There is a shortage of skilled staff for providing EmOC with 30% to 60% of level 2 facilities having no anesthesiologists or obstetricians; and 10% to 90% of facilities lack the kits required for normal delivery, essential/emergency obstetric care, and side lab test & blood transfusions. [India Facility Survey, Phase II, 2003]

		Percent of facilities					
**Facility** [a]	**Level**	No electricity	No operating room	No operating room for Ob/Gyn	No separate aseptic labor room	No linkage with blood bank	No quarters for RMO
District hospital	3rd	3.3%	0.5%	52.4%	56%	40%	53%
FRU	2nd	6%	6.3%	70.2%	67%	72%	58%
CHC	2nd	8.2%	12.4%	80.0%	69%	84%	56%
PHC	1st	34%	NA	NA	52%	NA	>40%
Subcentre	1st	57%	NA	NA	NA	NA	NA

a CHC=Community Health Centre; FRU=First Referral Unit; NA=Not available; PHC=Primary Health Centre (India Facility Survey, Phase II, 2003); RMO = Resident Medical Officer

Facility [a]	Level	Percent of facilities					
		No normal delivery Kit	No EmOC or EsOC Kit[b]	No Kit O[c]	No obstetrician	No anesthesiol-ogist	No female medical officer
District hospital	3rd	36%	10%	40%	10%	17%	5%
FRU	2nd	26%	27%	72%	29.5%	31%	12%
CHC	2nd	26%	64%	90%	49%	63%	20%
PHC	1st	50%	NA	NA	NA	NA	85%
Subcentre	1st	NA	NA	NA	NA	NA	NA

a	CHC=Community Health Centre; FRU=First Referral Unit; NA=Not available; PHC=Primary Health Centre (India Facility Survey, Phase II, 2003); EmOC = emergency obstetric care; EsOC = essential obstetric care
b	EmOC Kit=emergency obstetric care drugs kit, for District Hospital, FRU, CHC; EsOC Kit=essential obstetric care drug kit for PHC
c	Kit O=side lab test and blood transfusion kit

The following data from 2003 demonstrate the considerable lack of staff and supplies required for Reproductive and Child Health (RCH) within health facilities in India.

Facility [a]	Level	Inadequate Infrastructure [b]	Inadequate Human Resources [b]	Inadequate Supplies [b]	Inadequate Equipment [b]
District hospital	3rd	7%	21%	55%	16%
FRU	2nd	24%	63%	68%	39%
CHC	2nd	37%	86%	76%	54%
PHC	1st	68%	52%	60%	59%

a	CHC=Community Health Centre; FRU=First Referral Unit; NA=Not available; PHC=Primary Health Centre (Sharma 2002, data from IIHMR 1999).
b	Inadequate means having less than 60% of necessary Reproductive and Child Health (RCH) Inputs, as defined by India Facility Survey, Phase II, 2003.

There is considerable heterogeneity in the availability and quality of EmOC facilities across states. While Uttar Pradesh performs similarly to the national average on many indicators, it performs consistently worse on several key indicators, including lacking a female medical officer, lacking an obstetrician in level 2 and 3 facilities, and lacking EmOC kits. [India Facility Survey, Phase II, 2003] District level household and facility surveys demonstrated considerable limitations in services offered and accessibility of facilities in Uttar Pradesh and Rajasthan. [District Level Household and Facility Survey – DLHS-3, Uttar Pradesh, Rajasthan 2007-2008] Over 40% of PHCs were not functioning 24 hours; over 80% of FRUs did not offer cesarean section; more than 90% of PHCs had no female medical officers; 70% of CHCs had no obstetrician. [District Level Household and Facility Survey – DLHS-3, Uttar Pradesh, Rajasthan 2007-2008] Premature discharge of women occurred widely in Rajashtan; in one survey, 70% of women who had a vaginal delivery in a facility were discharged within 24 hours, and 14% 6 hours after delivery. (Ivengar 2009)

AMDD [2002] found that given the size of the population, Rajasthan has approximately one-third of the cEmOC (31%) and bEmOC (37%) facilities recommended. Additionally, policy barriers prevented the wider availability of blood (necessary for cEmOC) [AMDD 2002]. A comparison of the health systems of China and India [Ma 2008] found substantial shortfalls in India at each level of health facility, e.g., 50% fewer community health centers (CHCs) than needed [Datar 2007]. Moreover, care provided by the public sector is repeatedly described as *poor*, and care provided by the private sector is often deemed uneven [Bhatia 2004, Mills 2002].

In Uttar Pradesh [District Level Household Survey (DLHS) [IIPS 2006] and Facility Survey [IIPS 2005, IIPS 2007-08 Uttar Pradesh [IIPS Fact Sheet: Uttar Pradesh]), Government of India 2008a, Mills 2007], 13% of district hospitals and 60% of FRUs did not have an obstetrician while 50% of district hospitals and 75% of FRUs did not have anesthetists.

In Uttar Pradesh, of the 15 district hospitals surveyed, 10 provided cEmOC (67%), 1 provided only bEmOC, and 4 did not qualify as either (27%). Of the 54 first referral units (FRUs), 1 qualified as cEmOC (1.9%), while 43 did not qualify as bEmOC (10 were bEmOC, 18.5%). Of all facilities (n=69), 15.9% were cEmOC, 14.5% were bEmOC and 70% did not meet the requirements to be either bEmOC or cEmOC [Mills 2007]. Only two of the 54 FRUs had blood banks in 2006.

**Uttar Pradesh** [a] [Mills 2007]	***District Hospitals***	***FRUs***
**Emergency obstetrical care capacity**		
Less than basic emergency obstetric care	26.7%	79.6%
Basic emergency obstetric care	0.0%	13.0%
Basic emergency obstetric care and transfusions	6.7%	5.6%
Comprehensive emergency obstetric care	66.7%	1.9%
Manual removal of retained placenta	93.3%	72.2%
Removal of retained products of conception (MVA, D&C)	93.3%	20.4%
Assisted vaginal delivery	86.7%	46.3%
**Comprehensive EmOC capacity**		
Blood transfusion	73.3%	16.7%
Cesarean section	73.3%	1.9%

a FRU = First Referral Unit; MVA = manual vacuum aspiration; D&C = dilation and curettage; EmOC = emergency obstetric care

Katrak [2008] reprted that rural and urban areas differed in the total number of medical practitioners, as well as in the types or 'quality' of the practitioners. Deshpande et al. [2004] found that rural areas in one district in India had a smaller number of qualified doctors and a larger number of unqualified practitioners. Katrak [2008] combined those categories to create an overall measure of the number of practitioners, finding that women in rural areas faced greater challenges in accessing medical practitioners than in urban areas [also noted in Perry 2000, Wagstaff 2002, Buor 2003, Leonard 2003, Dzator 2004]. Because rural populations tend to be spread over a larger area, longer distances for travel are required to reach medical care and patients may have longer 'waiting times'. Katrak [2008] calculated a needs-to-access ratio, with the rural ratio in India at least 4.95 times greater than in urban areas and at most 14 times greater. An average of these two estimates implies that the rural shortage is about 9.5 times greater.

We conducted both sensitivity analyses on alternative baseline assumptions, as well as an analysis at every stepwise point. The Tables below show the initial *range* of baseline estimates used in sensitivity analyses, and the initial *range* across which stepwise improvements were made in the temporal strategies evaluated. These ranges were expanded in a series of exploratory analyses.

Range of baseline assumptions for status quo used in sensitivity analysis (available facility, staff and supplies, quality of care)

	India		Uttar Pradesh		Rajasthan	
Appropriate staff and supplies						
bEmOC facility	40%-60%		20%-40%		30%-55%	
cEmOC facility	40%-60%		25%-45%		30%-55%	
	Urban	Rural	Urban	Rural	Urban	Rural
Appropriate staff and supplies						
bEmOC facility	55%-80%	30%-55%	30%-50%	20%-40%	45%-60%	25%-50%
cEmOC facility	55%-80%	30%-55%	40%-60%	20%-40%	45%-60%	25%-50%

Upper bound of stepwise increases in available facility, staff and supplies, quality of care

	India		Uttar Pradesh		Rajasthan	
Appropriate staff and supplies						
bEmOC facility	50%-95%		30%-95%		43%-95%	
cEmOC facility	50%-95%		35%-95%		43%-95%	
	Urban	Rural	Urban	Rural	Urban	Rural
Appropriate staff and supplies						
bEmOC facility	68%-95%	42%-95%	42%-95%	26%-95%	55%-95%	39%-95%
cEmOC facility	68%-95%	42%-95%	50%-95%	31%-95%	55%-95%	39%-95%

Subsection C

Calibration Exercises and Model Performance

Calibration targets for each national and province-specific model are established based on survey data and published studies, and include the distribution of causes of maternal mortality (e.g., PPH, obstructed labor, sepsis), maternal mortality ratio (MMR), and the total fertility rate (TFR). The MMR is adjusted directly in the model for indirect causes of maternal-related mortality, as explained below. The importance of using multiple indicators is that different aspects of maternal mortality are reflected by each of them. For example, the MMR is not age-standardized, nor does it take into account the fact that women face the same risk numerous times over their reproductive lifespan, nor does it account for the reduction in risk attributable to declining fertility from family planning. The model can be used to project a range of maternal health indicators and these can be used as calibration targets, or can be compared to survey data to assess an approximation of face validity or projective validity. These include:

Maternal mortality rate	Defined as the number of maternal deaths per 1,000 women or 100,000 women of reproductive age (ages 15-45) or woman-years of risk exposure, and designed to be an indicator of risk of maternal death (i.e., cause-specific death rate)
Proportionate mortality ratio	Defined as the proportion of all female deaths among women of reproductive age due to maternal causes
Lifetime risk of maternal death	Reflects the probability of a maternal death during a woman's reproductive lifespan (the probability that a 15-year-old will eventually die from a maternal reason up to age 45, for example) and is described in terms of odds (it accounts for the probability of dying from maternal causes each time a woman experiences a pregnancy, and so takes into account fertility as well as obstetric risk)
Lifetime risk of dying from maternal causes	The calculation of lifetime risk assumes no changes in fertility or mortality; estimates are generated from the maternal mortality rate, and do take into account the competing causes of death. In contrast, in our model, the simulation over time does take into account the changes in fertility and background mortality, including changes in maternal mortality.

MMR

We used selected data for the India model MMR calibration target. We prioritized the recent estimate of 450 based on the reassessment of data by an international experts group that estimated the MMR to be 1.5 times the 2003 SRS estimate [Hill 2007; WHO 2007b]. We also took into consideration as an upper bound the estimate of 540 as reported in the 2005 World Health Report 2005, and as summarized by Mills et al. [2007]. Below, the range of MMRs using different sources and methods is provided. It is widely accepted that the error and uncertainty in these measures is formidable, and trends should be interpreted with grave caution. As sample sizes decrease, such at the state or district level, interpretation of trends should be avoided. That being said, the general pattern and rank ordering of state-level MMR estimates do provide an approximate categorization of states relative to one another. For example, there is a consistent rank order of urban India (best), India overall (next best) and rural India (worst), when considering the three main models and national and stratified data.

Revision of 2005 estimates of MMR for India

UNICEF, WHO, and the UN Population Fund (UNFPA) previously developed global, regional, and country estimates of maternal mortality for the years 1990, 1995, and 2000. [WHO, UNICEF. 1996; WHO, UNICEF, UNFPA, 2001; WHO, UNICEF, UNFPA, 2004] In 2006 a collaborative effort involving the WHO, UNICEF, UNFPA, the World Bank, and outside technical experts, reviewed the available data and developed revised estimates of maternal mortality for 2005. Countries were categorized on the basis of data availability, quality and type, and consensus was reached on methods of evaluation, data synthesis, and statistical modeling for estimation in countries with limited data. India was classified with China into a group characterized as estimating MMR from data from disease surveillance or sample registration systems. The recommendation for these countries was to consider the observed value as a lower uncertainty bound, double it for the upper bound, and multiply the observed value by 1.5 to yield the point estimate. [Hill 2007]. For India, this estimate was 450 (300-600). Data from the Sample Registration System (SRS) [Registrar General 2006], a series of government-run national surveys, report lower MMRs than those published by the WHO [2007b]. For example, the SRS 2001-2003 reports a national maternal mortality ratio at 301 maternal deaths per 100,000 live births. [Registrar General 2006]

We parameterized the state-level models for Rajasthan and Uttar Pradesh using the best information available and adjusted for the TFR as reported in NFHS 3. We then compared the model-projected MMR with reported data from those states. Note that the Rajasthan and Uttar Pradesh models were not calibrated to the MMR; rather we sought to assess model performance by comparing the model-projected MMR to reported data.

For Uttar Pradesh, reported MMRs to provide comparison for this exercise included the SRS 2001-2003 estimate of 517 (CI 461-573), SRS 1999-2001 estimate 539 (481-596), and the SRS 1997-98 estimate of 606 (CI 544-668) [Registrar General 2006]. Maternal deaths accounted for 26% of all deaths of women of reproductive age in 2001-2004. [Registrar General 2006]

For Rajasthan, we used the 2001-2003 special survey of deaths using RHIME which reported 445 (371-519), and SRS 1999-2001, which reported 501 (423-580). [Registrar General 2006] The lifetime risk of maternal deaths was estimated to be 1 in 53 (1.9%). When the MMR was 627, based on older data [Bhat 1995] the proportion of maternal deaths of all deaths among all deaths of women of reproductive age was 29%.

Documentation of maternal mortality ratios (MMR) reported in various sources [a]

Source	MMR	Range
**India**		
WHO, UNICEF, UNFPA, UNPD, World Bank, 2005[b] [Hill 2007; WHO 2007b]	450	300-600
WHO, UNICEF, UNFPA, 2000 [AbouZahr 2004; WHO 2005 (Annex Table 8)]	540	
SRS 1997-1998 [Registrar General 2006], From Retrospective MMR Survey; MM Rate 34.8, Lifetime risk 1.2%	398	378-417
SRS 1999-2001 [Registrar General 2006], From Prospective Household Report, MM Rate 31.2, Lifetime risk 1.1%	327	311-343
SRS 2001-2003, [Registrar General 2006], From Special Survey of Deaths using RHIME, MM Rate 27.4, Lifetime risk 1.0%	301	285-317
NFHS 1992-3, NFHS-1; Revised downwards at the time of NFSH2 [NFHS2 p. 196]	424	324-524
NFHS 1998-1999, [NFHS 2 Main Report, p.196]	540	428-653

**Uttar Pradesh**		
2001-2004 District Level Household Surveys, reported in Mills [c] [Mills 2007] MM Rate 67.3, Proportional risk 26.5	409	
SRS 1997-1998 [Registrar General 2006], From Retrospective MMR Survey MM Rate 86.7, Lifetime risk 3.0%	606	544-668
SRS 1999-2001 [Registrar General 2006], From Prospective Household Report; MM Rate 77.2, Lifetime risk 2.7%	539	481-596
SRS 2001-2003 [Registrar General 2006], From Special Survey of Deaths using RHIME, MM Rate 70.0, Lifetime risk 2.4%	517	461-573
[Mills 2007]	*707*	
**Rajasthan**		
1982-1986 based on Indian Ministry of Health and Family Welfare, [Bhat 1995] MM Rate 110, Proportional risk 29	627	
SRS 1998, [Registrar General of India,1998 (Analytical studies report no. 1)]	670	
SRS 1997-1998, [Registrar General 2006]; From Retrospective MMR Survey, 1997-1998	508	425-590
SRS 1999-2001, [Registrar General 2006] From Prospective Household Report, 1999-2001	501	423-580
SRS 2001-2003, [Registrar General 2006] From Special Survey of Deaths using RHIME, 2001-2003	445	371-519

a RHIME=Representative resampled, routine household interview of mortality with medical evaluation; SRS=Sample Registration System; NFHS=National Family Health Survey;

b The SRS and the vital registration system are considered to give underestimates, and an international experts group estimated the MMR to be 1.5 times the 2003 SRS estimate at ~450 (Hill 2007)

c District Level Household & Facility Survey of the Reproductive & Child Health Project (DLHS-RCH phase-2, round-2 survey)

Distribution of causes of maternal deaths – calibration data

A systematic review and analysis of the magnitude and causes of maternal deaths documents variation both across and within geographical regions. [Khan 2006] Estimates of specific causes of death in India are hindered by the same methodological challenges as in global estimates, further complicated by the considerable heterogeneity that exists. We used Khan 2006 regional estimates based on the large sample sizes, and took into consideration data from India as summarized below. The concordance was considerable in these estimates. Of note, anemia exerts a huge toll, contributing to 24% of all maternal deaths in one hospital study [Pendse 1999]. More recent data corroborate the role of anemia in maternal mortality (generally classified as an indirect cause) with Khan reporting 12.5% (Khan 2006) and Mills reporting 15% [Mills 2007].

The MMR is adjusted directly in the model for indirect causes of maternal-related mortality. We assume that the proportion of mortality that is categorized as indirect and attributable to anemia (~15%) will be reduced with strategies that include enhanced family planning, increases in appropriate antenatal care with completed courses of treatment for anemia, facility-based births with quality intrapartum care, and reliable access to basic and comprehensive EmOC. We conservatively assume that the proportion of mortality that is categorized as indirect and attributable to other causes will not be impacted.

**Maternal mortality causes (%)** [Khan 2006]	**S. Asia**	**(CI)**
Maternal hemorrhage	30.8%	5.9%-48.5%
Hypertensive disorders	9.1%	2.0%-34.3%
Obstructed labor	9.4%	0.0%-12.0%
Sepsis	11.6%	0.0%-13.0%
Abortion	5.7%	0.0%-13.0%
Subtotal	66.6%	
Other direct [a]	1.6%	0.0%-25.9%
Anemia [a]	12.8%	0.0%-17.3%
Other indirect [a]	12.5%	0.0%-29.2%
Unclassified [a]	6.1%	0.0%-16.2%
Total Indirect/Other [a]	*33.0%*	

CI= **Confidence Interval;** We assume that other direct 1.6 and unclassified 6.1, for a total of 7.8, is
a divided between direct and indirect causes for purposes of comparing the distribution of causes predicted by the model to the data. Thus, the indirect causes not attributable to anemia were assumed to approximate 18%.

**Maternal mortality (%)** [Mills 2007, Registrar General 2006]	**India**		**Nine states**	
		**CI**		**CI**
Maternal hemorrhage	38%	.34-.41	37%	.33-.42
Hypertensive disorders	5%	.03-.06	4%	.02-.06
Obstructed labor	5%	.03-.06	5%	.03-.07
Sepsis	11%	.09-.14	11%	.08-.14
Abortion	8%%	.06-.10	10%	.07-.12
Subtotal	67%		67%	
Indirect	33%		33%	

**Maternal mortality causes (%)**	**Uttar Pradesh** [Mills 2007]	**Rajasthan** [Pendse 1999]
Postpartum hemorrhage	29%	31%
Antepartum	4%	-
Postpartum	25%	-
Hypertensive disorders	4%	13%
Obstructed labor	11%	7%
Sepsis	8%	-
Abortion	11%	15%
Retained placenta	3%	-
Subtotal	66%	-
Other indirect	11%	-
Anemia	15%	24%
Total indirect	33%	-
Other	7%	-

Calibration required very minor adjustments across the plausible range established for the initial baseline parameters. These adjustments are shown below.

Adjustments to initial estimates of variables in calibration exercises

Variable [a]	India Urban	India Rural
Annual probability of pregnancy	0.91	1.1
Incidence of PPH	1	1
CFR of PPH	1	1
CFR for life-threatening complications [b] requiring transfusion, surgery, management of shock in the absence of emergency care	2-3	2-3
CFR for unsafe abortion	1	1

a PPH: postpartum hemorrhage; CFR: case fatality rate.
b distribution of severe life threatening complications was adjusted according to basic and comprehensive EmOC need, and is described in section II of this document in the section "severity of complications"; an explanation of the distribution of causes is described in section 5 of this document.

Model performance was assessed by comparison of model-based projections with reported measures such as life expectancy, proportionate mortality ratio, and population-based outcomes [WHO 2006, Registrar General 2006**,** AMDD 2002**,** UNICEF WHO UNFPA 2007, UN 2007]. Projective validity of the empirically-calibrated model was further assessed by simulating Rajasthan and Uttar Pradesh, and comparing projected maternal health indicators with reported data. [Registrar General 2006, Mills 2007] For the latter exercise, we parameterized the state-level models for Rajasthan and Uttar Pradesh using the best information available and adjusted for the TFR as reported in NFHS 3. We then compared the model-projected MMR with reported data from those states, e.g., the model projects a met need for EmOC, defined as the percentage of hospital-requiring complications actually treated in a referral-level facility, of 9.4%, which was within the range of the met need of EmOC of 5.3%-12.2% observed in Rajasthan, India [AMDD 2002].

Part III: Overview of Costs and Estimates

Overview: cost identification and measurement

Direct health care costs include the cost of a normal pregnancy (e.g., prenatal visits, normal labor and delivery), the cost of induced abortion, the cost of treating abortion-related complications, the cost of treating pregnancy-related complications (e.g., eclampsia, hemorrhage, sepsis), salaries of health care providers (e.g., counseling, skilled birth attendants, clinician time); costs related to prenatal care (e.g., additional prenatal visits, nutritional supplementation, treatment of anemia or other existing disease, screening for sexually-transmitted diseases [STDs]), providing safe abortion (e.g., manual vacuum aspiration) or family planning options (e.g., sterilization, intrauterine device [IUD], oral contraceptives), and emergency obstetric care (e.g., facilities with the capacity for transfusion, parental antibiotics, surgery, anesthesia). Direct non-health care costs include, but are not limited to, the costs of transportation to and from the clinic or provider, and costs of patient time seeking care or receiving care. Cost estimates are broken down by input (e.g., drugs, vaccines, salaries, infrastructure), by intervention (e.g., management of a normal birth, hemorrhage, eclampsia, sepsis), and by service location or level (e.g., hospital, health center, health post). Personnel cost (salaries) and facility costs are country-specific from International Labour Organization databases and data publicly available from the World Health Organization (WHO), and modified using state-specific data when available. Salaries originally reported in year 2000 International dollars were converted to local currency units using Purchasing Power Parity conversion rates, inflated to year 2006 local currency units using GDP deflators, and then converted to 2006 US dollars using exchange rates. When possible, we conducted literature reviews for costs associated with different services and complications; these costs were extrapolated and adjusted to the same year and currency to facilitate comparison and generate plausible ranges for each cost estimate. Differential costs of scale-up were assessed, as were training costs (e.g., for SBA).

Costs are presented in currency units that remove price inflation, and for analyses intended to inform resource allocation and compare studies from multiple countries, costs are expressed as US dollars or international dollars. While exchange rates may reflect under- or overvaluation of the local currency, they represent what is actually paid for locally produced inputs. Purchasing-power parity rates, in contrast, attempt to express what the local currency is worth in purchasing power, and therefore account for differences in price levels across countries. The exchange rate for domestic currency into international dollars is the amount of domestic currency required to purchase the same quantity of goods and services as $1 could purchase in the US.

Documentation of costs used in the India model

The India model requires country-specific estimates of all maternal interventions including safe abortion and long-term complications. Estimates in the current model were drawn from the UNFPA's Reproductive Health Costing Tools Model (RHCTM) [UNFPA 2007]. This model is designed to help countries estimate the cost of scale up for a basic package of reproductive health services – ranging from family planning, antenatal and delivery care to emergency obstetric care and HIV/STI prevention and treatment.

The RHCTM consists of two main parts. The first part estimates the direct costs associated with providing an essential package of 45 reproductive health interventions. Interventions evaluated in the current RHCTM include: (1) family planning; (2) antenatal care, including treatment for chlamydia, gonorrhea and anemia; (3) abortion (incomplete and elective) and post-abortion complications; (4) delivery care; (5) emergency/pre-referral care; (6) assisted vaginal delivery (EmOC treatment of obstructed labor); (7) cesarean section; (8) postpartum hemorrhage; (9) puerperal sepsis; (10) severe pre-eclampsia/eclampsia; (11) treatment of long-term complications

such as PID and obstetric fistula; and (12) postpartum care. The RHCTM also includes costs for additional maternal complications including: (1) premature rupture of membranes; (2) prolonged labor; (3) trichomoniasis and (4) antepartum hemorrhage.

RHCTM: Essential package of 45 reproductive health interventions

FAMILY PLANNING	ANC and Delivery Care	Obstetric Complications
Short-Term Methods	11 Antenatal Care (ANC)	18 Emergency Pre-Referral Care
1 Oral Contraceptives (Pill)	12 Treatment of Severe Anaemia	19 Prelabour Rupture of Membranes
2 Injectables	13 Hypertensive Disorders of Pregnancy	20 Prolonged Labour (>18 hours)
3 Condom - Male	14 Malaria Prevention within ANC	21 Forceps or Vacuum-Assisted Delivery (AVD)
4 Condom - Female	15 Malaria Treatment within ANC	22 Cesarean Section (C-Section)
Long-Term Methods	16 Delivery Care	23 Antepartum Haemorrhage
5 Intrauterine Device (IUD)	17 Postpartum Care	24 Postpartum Haemorrhage
6 Implant		25 Puerperal Sepsis
7 Sterilization - Female		26 Eclampsia/Severe Pre-eclampsia
8 Sterilization - Male		27 Postabortion Complications (PAC)
Other Methods		
9 Other		
10 Emergency Contraceptives (EC)		
**Other Maternal Conditions**	**HIV-related Interventions**	**Sexually Transmitted Infections**
28 Obstetric Fistula (OF)	Condom Programs targeting	40 Chlamydia
29 Urinary Tract Infection (UTI)	35a Commercial Sex Workers	41 Gonorrhea
30 Mastitis	35b Men who have Sex with Men (MSM)	42 Syphilis
	35c Adolescents (age 15-24)	43 Trichomonas
**Newborn Interventions**	35d Other vulnerable population	44 Pelvic Inflammatory Disease (PID)
31 Routine Newborn Care	36a Antiretroviral Therapy (ARV) First Line	45 Cervical Cancer Screening
32 Newborn Sepsis / Infections	36b Antiretroviral Therapy (ARV) Second Line	
33 Birth Asphyxia / Breathing Difficulties	37 Prevention of Mother-to-Child Transm. of HIV (PMTCT)	
34 Low Birth Weight	38 Voluntary Counseling and Testing for HIV (VCT)	
	39 Post-Exposure Prophylaxis (PEP)	

The second part costs out activities and investment required to improve the health system of a country in order to scale up and provide the above package of reproductive health interventions. This includes investments in the physical and human infrastructure (building, rehabilitating, and equipping medical facilities; training and retaining staff; improving the referral and medical supply system) as well as demand creation, outreach, supervision, monitoring and evaluation activities.

The RHCTM uses an ingredients approach to estimate the costs associated with an intervention. Each complication is associated with a drug, supplies, and personnel requirements for treatment. However, the estimate does not include costs associated with occupying a health facility bed or an outpatient visit; these costs were obtained from the WHO CHOICE database and India-specific estimates were used.

Most of the data in WHO CHOICE are from UN sources such as the UN Population Division, WHO's Global Burden of Disease and other databases, UNICEF's maternal health database, and Demographic and Health Surveys. The lists of drugs and supplies required to provide the interventions are based on WHO treatment guidelines [Adam 2005]. Costs are presented in 2006 US$, and drug prices are based on quotes from the UNICEF Supply Catalogue and the MSH International Drug Price Indicator [UNICEF Supply Division, MSH IDPIG]. Personnel cost/salaries are based on information provided by WHO CHOICE. Salaries originally reported in 2000 I$ were converted to local currency units using methods described above. These estimates were compared to estimates obtained from the International Labour Organization [ILO Laborsta], and the newest available estimates for personnel for each category were used.

Annual personnel costs by category	2000 LCU	2006 LCU	2006 US$
Auxiliary/Attendant	57,762	73,716	1,627
Nurse/Midwife	82,338	105,081	2,319
General Physician	218,586	278,962	6,157
Obstetrician	380,470	485,560	10,716
Paediatrician	380,470	485,560	10,716
Anaesthesist	380,470	485,560	10,716
Lab Technician	82,338	105,081	2,319

LCU: local currency unit

The RHCTM does not include estimates of facility costs per case. For this, we drew on the India-specific estimates of unit costs for patient services provided in WHO-CHOICE. Since many interventions can occur outside a 20-minute visit time frame (e.g., 5 minutes or 30 minutes), we broke down the cost for outpatient visits according to an estimated cost per minute.

Facility costs originally presented in 2000 I$ and local currency units (LCU) were converted to 2006 US$ in a similar fashion as described above.

WHO CHOICE facility costs	2000 LCU	2006 LCU	2006 US$
Primary	214.14	273.29	6.03
Secondary	279.37	356.53	7.87
Tertiary	381.59	486.99	10.75
Outpatient visit by hospital level			
Primary	57.64	73.56	1.62
Secondary	81.76	104.34	2.30
Tertiary	120.95	154.36	3.41
Health center costs (by coverage level)			
50%	93.61	119.47	2.64
80%	93.61	119.47	2.64
95%	101.77	129.88	2.87

LCU: local currency unit

In the following intervention-specific sections, we present cost estimates used in the model, followed by tables outlining how these costs were derived from the RHCTM for the following intervention components: (1) drugs and supplies per case; (2) personnel costs per case; and (3) facility costs per case.

Family Planning

Oral Contraceptives
We assumed that oral contraceptives are distributed at a health post and the cost under the current standard of care in India is $10.64 per year.

Category	Component cost (2006 US$)
Drugs and supplies	$5.09
Personnel	$0.94
Facility charges	$4.61
**Total Cost**	**$10.64**

Injectables
The cost of providing injectable contraceptives is $10.20 per year.

Category	Component cost (2006 US$)
Drugs and supplies	$3.85
Personnel	$1.07
Facility charges	$5.27
**Total Cost**	**$10.20**

Condoms
The cost of providing condoms is $8.40 per year. We assumed that condoms are obtained at an outpatient health post visit. In a scenario where the use of family planning is increased, costs could be reduced through alternative delivery methods that eliminate this visit and its associated costs.

Category	Component cost (2006 US$)
Drugs and supplies	$2.85
Personnel	$0.94
Facility charges	$4.61
**Total Cost**	**$8.40**

Intrauterine device (IUD)
The annual cost of IUD use is $9.17.

Category	Component cost (2006 US$)
Drugs and supplies	$1.24
Personnel	$1.34
Facility charges	$6.59
**Total Cost**	**$9.17**

Female sterilization
The cost for female sterilization is $18.98.

Category	Component cost (2006 US$)
Drugs and supplies	$4.73
Personnel	$4.40
Facility charges	$9.85
**Total Cost**	**$18.98**

Male sterilization
The total cost for male sterilization is $12.67

Category	Component cost (2006 US$)
Drugs and supplies	$0.88
Personnel	$1.94
Facility charges	$9.85
**Total Cost**	**$12.67**

Antenatal care, including treatment for chlamydia, gonorrhea and anemia

The cost of antenatal care under the current standard of care is $17.07. This total reflects the cost of visits, including drugs, personnel and treatment for STI's.. It includes tetanus vaccination, syphilis testing and treatment if necessary, GC/chlamydia testing and treatment if necessary, urinalysis for glucose, ketones, pH, plus four visits, counseling and education (family planning, birth spacing, parenting, etc). We added the cost of anemia treatment (iron folate supplementation), shown below.

Antenatal care (4 visits)

Category	Component cost (2006 US$)
Drugs and supplies	2.86
Personnel	3.66
Facility charges	10.55
**Total Cost**	**$17.07**

The cost of treatment for anemia is added to the cost of prenatal care shown above. The management of severe anemia is not specified in the RHCTM, but was adapted based on the PPH protocol of the RHCTM, $1.02.

Anemia treatment

Category	Component cost (2006 US$)
Drugs and supplies	
Supplement (postpartum)	$0.68
Moderate	$0.68
Severe	$1.02

A sensitivity analysis was conducted to assess a community based intervention for SBA-administered misoprostol to reduce incidence of PPH in home deliveries and in birthing centres. We assumed $4.40 ($3.40 based on the upper bound used by Sutherland and Bishai (2009) of training costs for SBA, and .99 for misoprostol). These costs represent the incremental costs above routine SBA delivery.

Incomplete abortion, elective abortion, management of post-abortion complications

Incomplete abortion

We assumed all women with incomplete abortion from miscarriage were managed with manual vacuum aspiration at a cost of $8.90.

Category	Component cost (2006 US$)
Drugs and supplies	$1.26
Personnel	$1.61
Facility charges	$6.03
**Total Cost**	**$8.90**

Elective abortion

Available data on the cost of abortion in India are sparse and do not distinguish between the cost of safe and unsafe abortion. We assumed the cost of elective abortion was 750 Rupees (Rs) based on a published study with a range of 500-1000 Rs [Duggal 2004]. This estimate was inflated from year 2000 Rs to 2006 Rs and then to 2006 US$, for a total cost of $21.87.

Post-abortion complications
Using the assumptions of the RHCTM, the cost of post-abortion complications was estimated at $43.40; assumptions included: (1) 100% requiring manual vacuum aspiration; (2) 25% requiring treatment of sepsis; and (3) 25% requiring repair of tears.

Supplemental literature-based estimates for the cost of elective abortion
The cost of safe and unsafe abortion was also estimated in a series of unpublished studies available from CEHAT. The year associated with these costs is not reported but since the CEHAT studies commenced in the year 2000, we assume the costs reported are in year 2000 Rs. Of note, CEHAT also provides an estimate of the out-of-pocket cost of spontaneous abortion (components not specified) of 1113.7 Rs. [CEHAT 2001]

Cost of induced abortion (Rs) (CEHAT 2001)

Location	Provider/Timing				Lost wages per family
	Public-1st trimester	*Public-2nd trimester*	*Private-1st trimester*	*Private-2nd trimester*	
**Tamil Nadu**	759		1335 (3571 with sterilization)		287
**Average**	1334 (950 median)				1622 (1000 med.)
**Rajasthan**	195-457	317-575	540-724	1144-1681	
**Orissa**	57 (Low)		1260 (High)		
**Madhya Pradesh**	286	775 (12-20 wks)	559	1321 (12-20 wks)<br>1583 (> 20 wks)	
**Maharastra**	1746.5 (average out of pocket; variation private vs public 11x, hospital cost 20x)				

For additional data on the general cost of abortion please refer to (1) Hu [2009]; (2) Henshaw [2008]; (3) Lule [2007]; (4) Neogi [2007]; (5) Vlassof [2008]. These reports were used to establish plausible ranges for an upper and lower bound for sensitivity analyses.

Delivery Care

We assume that for births that take place at home there are three possible levels of care: assistance by a family member, assistance by a traditional birth attendant (TBA), and assistance by a skilled birth attendant (SBA). For home deliveries, we include the cost of the attendant's time but there are no facility charges, and in the base case, no charges for drugs (except for in the case of special analyses, in which we assessed the use of misoprostol). Deliveries at a primary-level health post (i.e., subcentre or primary health centre) utilize the following assumptions: all deliveries are attended by skilled staff, requires half a bed day, has mechanisms for referral to facility with EmOC; functions of bEmOC are not assumed to be present. For deliveries at a secondary-level health centre (i.e., community health center or first referral unit), basic EmOC is expected to be available. For deliveries at a tertiary-level facility (i.e., district hospital or medical centre), comprehensive EmOC is expected to be available. To model the relationship between the costs of delivery estimated using the RHCTM tool ingredients-based approach for normal delivery and the costs of delivery at different levels of facilities, we applied a scaled factor to reflect the higher costs

in a tertiary hospital versus secondary-level facility versus a birthing centre or primary care health post. The scaling factor relied on the relative costs reported in the regional and country-specific WHO CHOICE databases for primary-, secondary- and tertiary-level facility bed-days and visits, as well as the relative costs of health provider salaries based on a distribution of increasingly skilled health providers comparing a primary-level birthing centre or subcentre to the tertiary-level hospital (e.g., specialists versus medical officers versus nurses). Results are shown below with bEmOC facilities being 1.7 times (1.30 -1.97) more costly than primary-level health centres, posts or birthing centres; tertiary facilities such as district hospitals and general cEmOC capability are 2.25 (1.78-2.56) times more costly than primary-level facilities. Mills et al. [2007] reported costs in Uttar Pradesh approximately 3.2 times higher in the district hospital compared with a community health centre; this was used as an upper bound in sensitivity analysis. Data from rural Rajasthan reported similar results [Iyengar 2009a] with delivery costs at government district hospitals approximately 2.47 times higher than a government primary health centre. All costs were varied in sensitivity analysis.

Facilities in the model [a]

**Health Facility in India** [b]	**Level**	**Model Category**	**Factor to Scale Costs** [c]
Medical centre	3rd	Tertiary facility w/cEmOC	2.25 (1.78-2.56)
District hospital	3rd		
First Referral Unit	2nd	Health centre/bEmOC	1.7 (1.30-1.97)
Community Health Centre	2nd		
Primary Health Centre	1st	Birthing centre	
Subcentre	1st		

a Facility levels in the model are categorized generally as (1) *primary-level facilities,* which may not have all bEmOC functions but could function as birthing centres with SBA staffing, 24-hour intrapartum care, and reliable referral connections; (2) *secondary facilities* with bEmOC capacity; and (3) *tertiary facilities* with cEmOC capacity. We recognize that some tertiary sites will not have a blood bank and some secondary sites may eventually be able to perform c-section; further, we recognize that in the strategies that include stepwise investments in infrastructure and facility improvements, not all facilities will be expected to be fully implemented as one of the three distinct types. However, because the costs, functions and staffing are fairly closely aligned with basic or comprehensive EmOC capacity, this simple categorization captured the most important dimensions for purpose of this analysis.

b Public-health facilities in India as described in Vora [2009]

c Factors based on data from WHO-CHOICE

Costs associated with routine delivery	Component cost (2006 US$)
**Home**	$0
Traditional birth attendant	$4.52
Skilled birth attendant	$6.44
**Birthing centre - primary-level health post (i.e., subcentre or primary health centre)**	
Drugs and supplies	$3.50
Personnel	$6.44
Facility charges	$4.52
Total Cost	**$14.46**
**Secondary-level health centre (i.e., community health center, 1st referral unit) - bEmOC**	
Drugs and supplies	$5.95
Personnel	$10.95
Facility charges	$7.68
Total Cost	**$24.58**

Tertiary-level (i.e., district hospital or medical centre) - comprehensive EmOC	
Drugs and supplies	$7.88
Personnel	$14.49
Facility charges	$10.17
Total Cost	**$32.54**

To place our base case estimates in context, other delivery cost sources are shown below. In Uttar Pradesh, delivery fees were found to be much higher in private nursing homes than in private hospitals. [Mills 2007] The average fee for a normal delivery in a private nursing home was almost 2,000 Rupees (US$44, at 45 Rupees to the dollar), 2.5x the cost in a private hospital, 7x the cost in a district hospital. A sample of recent delivery costs reported for rural Rajasthan [Iyengar 2009a] and poor women in Gujarat [Bhat 2009] are shown below. A sample of older delivery costs, reported by Borghi et al. [2006], is also shown below.

Other delivery costs in India

Iyengar 2009a (rural Rajasthan)	US ($)
Government subcentre	$15.56
Government PHC (primary health centre)	$22.89
Government CHC (community health centre)	$23.00
Government District Hospital	$56.42
Bhat 2009 (Gujarat)	
Facility with bEmOC	$31.53
Package (Private facility) [a]	$54.40

a Subsidized as part of the Chiranjeevi Scheme, a public-private partnership in which the private sector provides services and the public sector negotiates fixed prices to reduce financial risk to women [Bhat 2009]

Taken and adapted from Borghi [2006]

Study	Setting	Year	Delivery (hospital)	2004 I$	C-section/compl	2004 I$
Borghi 2003	Benin	2002	15-36	32.25-77.41	60-269	129.02-578.44
Borghi 2003	Ghana	2002	19-23	92.53-112.01	59-132	287.33-642.83
Kowalewski 2002	Tanzania	1997-98	9*	20.28	10	22.53
Afsana 2004	Bangladesh rural	2000-01	31*	148.52	250-385	1197.72-1844.48
Nahar 1998	Bangladesh urban	1995	32*	153.31	118	565.32
Borghi 2006	Nepal	2004	67*	366.52	132	722.1

*includes transport costs

Emergency/pre-referral care (transportation costs)

Costs for transportation include the cost of transportation for a woman with a recognized complication that cannot be treated at the original birthing location (either true complication or false referral), and the cost for an attendant to accompany the woman during transport in some circumstances. Of note, the model permits additional costs to be assigned to the category of transfer from home to a facility that reflect interim lifesaving measures such as management of PPH and use of the non-pnuematic anti-shock garment (NASG), although this is not included in the base case for this analysis.

There is a relative lack of data available on the cost of transportation in India from one birthing location (home, health post, bEmOC), to a higher-level facility (health post, bEmOC, cEmOC). We were able to leverage some data on transportation from recently published papers [Bhat

2009; Iyengar 2009a], and used data from other sectors to approximate increased costs associated with improving transport programs in rural regions.

Costs associated with transfer/transport

Model assumptions [a]	***Baseline***	***Upgrade 1***	***Upgrade 2***	***Upgrade 3+***
Transport (home to birthing centre)	$3.62	$7.23	$9.04	$10.85
Transport (home to primary facility-bEmOC)	$6.15	$12.29	$15.36	$18.44
Transport (home to tertiary facility-cEmOC)	$8.13	$16.27	$20.33	$24.40
*Transport (primary to tertiary facility-cEmOC)**	$7.14	$14.28	$17.85	$21.42
Transport (birthing centre to EmOC)	$4.88	$9.76	$11.57	$14.64

a base case estimates were varied from 0.50x the base case (lower bound) to 5x the base case (upper bound sensitivity analysis)

Transport [Bhat 2009]	***Estimate 1*** [a]	***Estimate 2*** [a]
normal delivery	$5.70-$6.80	$6.13
complicated delivery	$7.30-$8.20	$8.20
transport plus accompanying person	*$1.25*	
Transport, rural Rajasthan [Iyengar 2009a]		
Elective vaginal delivery in health facility	$9.60	
vaginal delivery, facility (referred after problem)	$12.80	
Caesarean delivery	$19.58	

a Estimate 1 and 2 from the two groups in the program

Using public access data, we also developed a transport cost calculator that incorporates information on distance, road density, cost of vehicle and fuel, and approximates a cost. Data used to develop this cost estimation tool were derived from various sources including the World Bank and national databases on road transport. [World Bank Data Pages, IEA 2006, Automobile India, Government of India 2008b, Maps of India, India.co.in] This allowed for rough approximations for face validity using studies that reported mean distance. For example, Bhat et al. [2009] reported for Gujarat state that the mean distance traveled for women participating in a public-private partnership (Chiranjeevi Scheme, which focused on increasing institutional delivery and emergency obstetric care for the poor) was 13.8 km but ranged from 1 to 72 km. Using the tool, we estimated the mean cost to be $2.90 with an upper bound of $8.00.

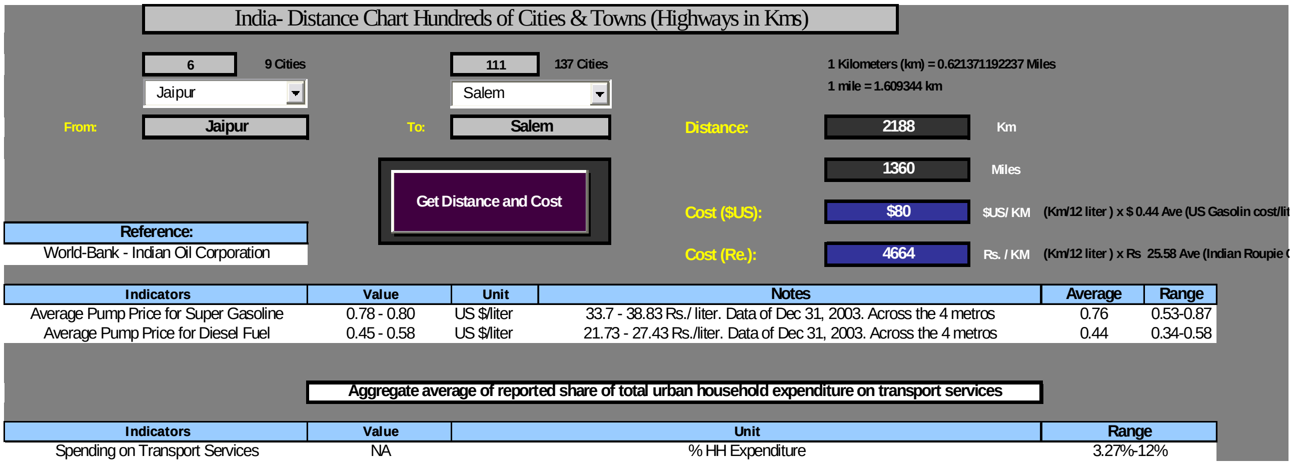

Management/treatment of complications

Management/treatment obstructed labor

Management of obstructed labor at bEmOC consists of assisted vaginal delivery with vacuum or forceps. In addition to obstructed labor costs, we also included the cost of prolonged labor, which precedes the diagnosis of obstructed labor. At the bEmOC level (where cesarean section is not available), the total cost is $19.51. In comparison, Bhat et al. [2009] reported a cost per procedure involving forceps, vacuum, breech (possible in bEmOC) of $22 in Gujarat. At the cEmOC level, the cost of c-section is $92.92. In comparison, Bhat et al. [2009] reported the cost per procedure involving c-section (as would be conducted in cEmOC) was $111 in Gujarat.

Category (bEmOC)	Component cost (2006 US$)
Drugs and Supplies	$6.00
Personnel	$7.48
Facility Charges	$6.03
**Total Cost**	**$19.51**

Category (cEmOC)	Component cost (2006 US$)
Drugs and Supplies	$12.24
Personnel	$25.60
Facility Charges	$55.08
**Total Cost**	**$92.92**

Management/treatment for postpartum hemorrhage

Managing PPH with basic services at a bEmOC-level facility costs $29.35; for those cases needing transfusion, advanced shock management, and/or surgery at cEmOC, the cost is $141.67. The difference in the cost of management/treatment for PPH reflects primarily the lack of capacity to perform emergency transfusions at a bEmOC facility and increased personnel and facility charges at cEmOC facilities.

Category (bEmOC)	Component cost (2006 US$)
Drugs and Supplies	$7.44
Personnel	$9.85
Facility Charges	$12.06
**Total Cost**	**$29.35**

Category (cEmOC)	Component cost (2006 US$)
Drugs and Supplies	$87.32
Personnel	$22.88
Facility Charges	$31.48
**Total Cost**	**$141.67**

In comparison, Borghi et al. [2003] report the cost for treatment of PPH in Benin and Ghana and Weissman et al. [1999] for Uganda. When these costs are converted to 2006 US$ using the methods described earlier, our estimates from the RHCTM fall within the plausible bounds reported in the literature, which ranged from $46 to $198.

Hemorrhage	Cost (2006US$)
Uganda (public hospital)	45.89
Uganda (mission hospital)	72.66
Ghana Teaching Hospital	197.45
Benin Teaching Hospital	117.87
Benin Non-Teaching Hospital	76.62

Management/treatment of puerperal sepsis.

At a bEmOC-level facility, the cost is $32.59; for cases needing transfusion, advanced shock management, and/or surgery at cEmOC, the cost is $74.01. In comparison, Bhat et al. [2009] reported a cost of sepsis requiring cEmOC of $66.70 in Gujarat, closely approximating a weighted average of the proportion requiring basic and comprehensive EmOC.

Category (bEmOC)	Component cost (2006 US$)
Drugs and Supplies	$13.71
Personnel	$6.81
Facility Charges	$12.06
**Total Cost**	**$32.59**

Category (cEmOC)	Component cost (2006 US$)
Drugs and Supplies	$34.79
Personnel	$7.74
Facility Charges	$31.48
**Total Cost**	**$74.01**

In addition**,** Borghi et al. [2003] report the cost for treatment of sepsis in Benin and Ghana and Weissman et al. [1999] for Uganda. When these costs are converted to 2006 US$ using the methods described earlier, our estimates fall within this range.

Sepsis	Cost (2006US$)
Uganda	11.23
Uganda	36.11
Ghana Teaching Hospital	177.71
Benin Teaching Hospital	184.17
Benin Non-Teaching Hospital	75.88

Management and treatment of severe pre-eclampsia/eclampsia

The cost of managing hypertensive disorders and treating eclampsia at bEmOC facilities is $59.80 and $102.41 in cEmOC.

Category (bEmOC)	Component cost (2006 US$)
Drugs and Supplies	$7.20
Personnel	$10.38
Facility Charges	$42.22
**Total Cost**	**$59.80**

Category (cEmOC)	Component cost (2006 US$)
Drugs and Supplies	$22.30
Personnel	$25.03
Facility Charges	$55.08
**Total Cost**	**$102.41**

In comparison, older data, Weissman et al. [1999], report the cost for treatment of hypertensive disorders (HTN) in Uganda. When these costs are converted to 2006 US$ using the methods described earlier, our estimates from the RHCTM fall within plausible bounds reported in the literature, from $73 to $145.

HTN	Cost (2006US$)
Uganda	72.96
Uganda	145.09

Treatment of long-term complications such as PID and obstetric fistula

India-specific costs for most long-term sequelae stemming from maternal complications are not available. In their absence we relied on published cost estimates from a variety of developing and developed countries, also used in a previous analysis. [Hu 2007]

Treatment of pelvic inflammatory disease

The cost associated with treating PID is $4.83, assuming all PID is treated on an outpatient basis.

Category	Component cost (2006 US$)
Drugs and supplies	$0.64
Personnel	$0.89
Facility charges	$3.30
**Total Cost**	**$4.83**

Treatment of obstetric fistula

The RHCTM provides an estimate of the cost of repairing an obstetric fistula of $87.91.

Category	Component cost (2006 US$)
Drugs and supplies	$12.58
Personnel	$20.24
Facility charges	$55.08
**Total Cost**	**$87.91**

A supplemental review of the literature surrounding treatment/repair of obstetric fistula showed that the estimates produced by the RHCTM were much lower than found in the literature and costing reports. For example, in 2003 Engender Health reported the cost of obstetric fistula repair in 9 African countries to be $300 (converted from 2002 to 2006 US$) with a range of $100-$400. We revised our estimate used in the model to be $226, but varied it from the lower bound obtained with the RHCTM to an upper bound of $400.

Postpartum care

Postpartum care includes a 30-minute visit by a skilled health provider and distribution of iron/folate supplementation. Under the current standard of care this cost is $4.99.

Category	Component cost (2006 US$)
Drugs and Supplies	$0.23
Personnel	$0.81
Facility Charges	$3.95
**Total Cost**	**$4.99**

Costs of scaling up and costs in sensitivity analysis

As shown in the figure below, investments for strategies that included stepwise improvements in intrapartum care fall into the following general categories:

(1) **average normal delivery (differentiated by site)** to reflect (a) recruiting and training cadre of SBA, (b) improving recognition of referral need via training of SBA, as well as education for woman and family, and (c) interim care by SBA prior to transport;

(2) **transfer from delivery site to referral facility (differentiated by origin and destination)** to reflect (a) transport cost; (b) vehicle use and fuel; (c) interim care en route separate from routine SBA training;

(3) **expedient attention at appropriate referral facility (differentiated according to bEmOC or cEmOC services)** to reflect (a) new and/or improvements in existing primary facilities (bEmOC) including ensuring 24-hour access; (b) new/and or improved secondary and tertiary facilities (cEmOC); (c) blood bank and transfusion capability, enhanced surgical capacity, intensive care support functions for shock in cEmOC; (d) improved quality of care in bEmOC and cEmOC with adequate supplies and personnel.

Figure. Cost increases to reflect investments in infrastructure

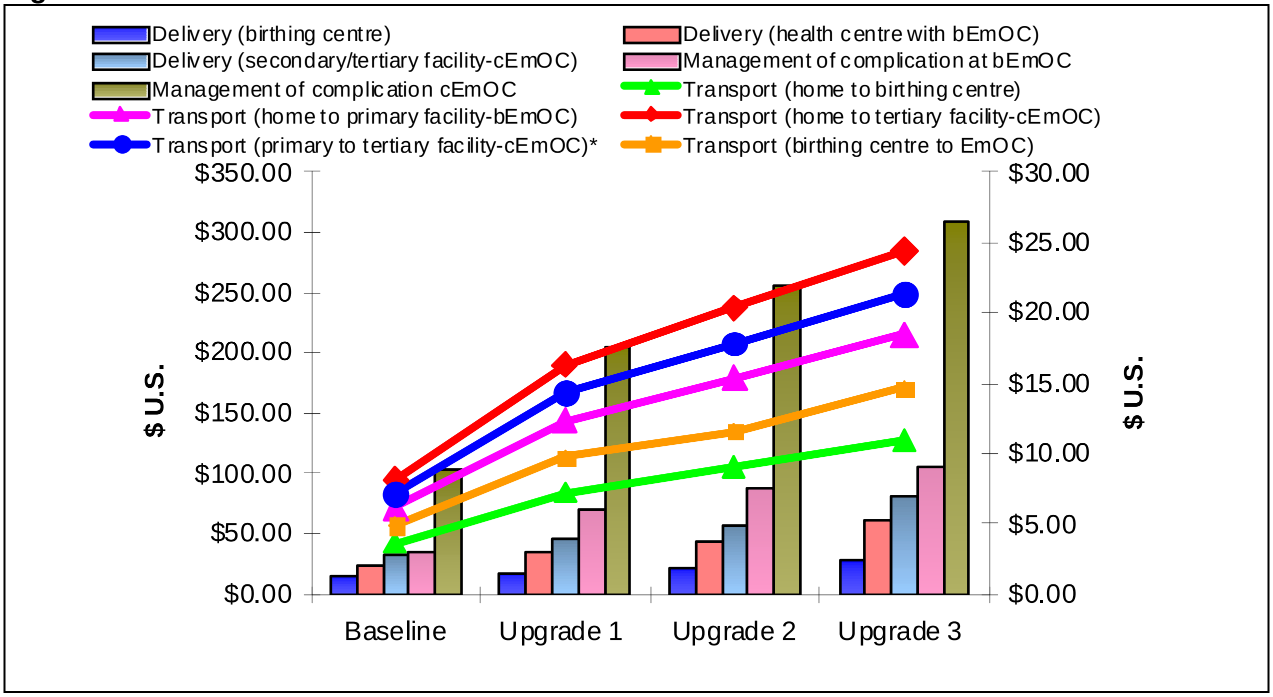

Comparison of scale-up cost implications

There are virtually no empiric data to inform the incremental costs of a massive effort to invest in infrastructure, human resources, and facilities to improve maternal health. Since our incremental "upgrade" costs were at best, rough approximations, we felt it was prudent describe these costs in the context of projected estimates made by others. (Bhat 2009; Graham 2006; Borghi 2006; Johns 2007). We also leveraged published studies that estimated the global resource requirements for scaling up interventions to reduce maternal mortality to assess the face validity of our assumptions. (Borghi 2006; Graham 2006; Johns 2007) **We outline the findings from selected comparisons below.**

The Gujarat state developed a public-private partnership called the Chiranjeevi Scheme, which focuses on institutional delivery and emergency obstetric care for the poor. Although one of the most important outcomes was financial protection from catastrophic costs associated with a complication, a range of cost proxies (for resources required) were provided. [Bhat 2009] The estimate of costs associated with complications in delivery was $209.33 (among the participants who had a prior complicated delivery). **The average cost of complicated deliveries in our scale-up scenarios, which most closely approximate the level of care reported by Bhat et al. [2009], ranged from $172.58 to $207.33.**

Johns et al. [2007] found that increasing coverage scale-up targets by 27% (from an average of 73% coverage to 95%) resulted in a 42% increase in total costs. **We found that our assumptions of increased costs for later phase improvements (e.g., more than 50% of the population covered), implied that a 33% increase in coverage resulted in an approximate 40% increase in total costs and a 36% increase in discounted lifetime costs.**

Johns et al. [2007] found that overall, the primary-care level comprises 29% of costs for moderate scale-up and 30% for rapid scale-up, with referral care accounting for 44% of costs in the moderate scale-up scenario and 47% in the rapid scale-up scenario. The remaining 27% in the moderate scale-up scenario and 23% in the rapid scale-up cover programme development and investments in health infrastructure. The relative costs of secondary versus primary costs were 1.57 times higher. **We found that our assumptions of increased costs for stepwise improvements implied costs of referral care in secondary and tertiary facilities were 1.56 times the costs of primary-care level delivery and management.**

Johns et al. [2007] found that overall, the cost of transport was 5% of the total cost of scale-up, and infrastructure comprised 15% of the total cost of scale-up. **We found that our assumptions of increased costs for transport from birthing centres to secondary and tertiary facilities ranged from 4.3% to 6% of the total cost of scale-up and transport from home to secondary and tertiary facilities ranged from 4.7% to 12%. Our estimates of transport costs included some costs categorized as infrastructure by Johns et al. [2007].**

Johns et al. [2007] found that, using multiple countries within the South-East Asia region, the average cost per capita per year (2006-2015) for rapid scale-up of maternal and newborn health care interventions ranged from $0.66 for moderate scale-up to $1.12 for rapid scale-up, for total incremental costs of $12 billion to $20.5 billion.

**Johns [2007]**	Average cost per capita per year 2006-2015 (US$)	Total incremental cost, 2006-2015
	**South-East Asia population**	
Rapid scale-up	1.12	20,500,000,000
Moderate scale-up	0.66	12,000,000,000

Using these same data, but applying the populations for India from the United Nations Population Division's World Population Prospects database [UN 2007], we approximate their $8.1 billion to $13.8 billion over the same time period.

**Calculations**	Average cost per capita per year 2006-2015 (US$)	Total incremental cost, 2006-2015
	**Using India Population**	
Rapid scale-up	1.12	13,753,439,187
Moderate scale-up	0.66	8,104,705,235

When we divide these totals by the population of women of reproductive age in India in 2006 (ages 15-44) we approximate the total average cost per woman of reproductive age for the 10-year period modeled by Johns et al. [2007] to range from $31.07 (moderate scale-up) to $52.73 (rapid scale-up).

Per woman aged 15-44	Rapid scale-up (achieves 80% coverage by 2015)	52.73	per capita incremental cost of scale-up divided by number of women of reproductive age (for 10-year period 2006-2015)
	Moderate scale-up (achieves 75% coverage by 2015)	31.07	

The undiscounted 10-year costs per woman of reproductive age for scale up of coverage predicted by our model range from $4.39 in the urban setting and $5.30 in the rural setting for Upgrade 1 (increase to 45% facility births) to $24.91 in the urban setting and $51.37 in the rural setting for Upgrade 4 (increase to 80% facility births). The incremental costs per woman of reproductive age over a 10-year period for Upgrades 3 and 4 are similar to the computed costs (above) using the Johns [2007] cost estimates, which both reflect increases in coverage to 75-80%.

**Urban India**	Total undiscounted 10-year life-time costs, per woman of reproductive age		Increased costs of scale-up per woman of reproductive age (for a 10-year period 2006-2015)
Strategy (% births in facility)	No scale-up costs	With scale-up costs included	
Current (40%)	$67.65	---	---
Upgrade 1 (45%)	$69.62	$74.01	**$4.39**
Upgrade 2 (60%)	$71.70	$79.07	**$7.37**
Upgrade 3 (75%)	$73.94	$95.20	**$21.26**
Upgrade 4 (80%)	$76.62	$101.53	**$24.91**
**Rural India**	Total undiscounted 10-year life-time costs, per woman of reproductive age		Increased costs of scale-up per woman of reproductive age (for a 10-year period 2006-2015)
Strategy (% births in facility)	No scale-up costs	With scale-up costs included	
Current (30%)	$65.96	---	**---**
Upgrade 1 (45%)	$69.98	$75.28	**$5.30**
Upgrade 2 (60%)	$74.34	$88.89	**$14.55**
Upgrade 3 (75%)	$79.45	$118.45	**$39.00**
Upgrade 4 (80%)	$84.91	$136.28	**$51.37**

When we divide these totals by the population of women of reproductive age in India in 2006 (ages 15-44) we approximate the total average cost per woman of reproductive age for the 10-year period modeled by Johns et al. [2007] to range from $31.07 (moderate scale-up) to $52.73 (rapid scale-up).

**Our model strategies**	**Incremental Costs Predicted by our model**	**Johns strategies (2007)**	**Incremental Costs Inferred by Johns analysis (10 year period)**
Urban: Upgrade 3 (75%)	$21.26		
Urban: Upgrade 4 (80%)	$24.91		
Rural: Upgrade 3 (75%)	$39.00	Moderate Scale-up	$31.07
Rural: Upgrade 4 (80%)	$51.37	Rapid Scale-up	$52.73

Part IV: Additional Results

Supplemental Results Table 1. Health and economic outcomes Uttar Pradesh and Rajasthan

Supplemental Results Table 2. Sensitivity Analysis: Family Planning

Supplemental Results Table 3. Sensitivity Analysis: Antenatal Care

Supplemental Results Table 4. Sensitivity Analysis: Distribution of Routine Births in Facilities

Supplemental Results Table 5. Sensitivity Analysis: Comparative Analysis to Pagel (2009)

Supplemental Results Table 6: Benefits and cost-effectiveness of improved intrapartum care in India

Supplemental Results: Uttar Pradesh and Rajasthan

Table 1. Strategies to Reduce Maternal Mortality in Uttar Pradesh, Rajasthan [a]

Strategy	Reduction in maternal deaths	MMR	Average lifetime costs		ICER ($/YLS)
			Baseline	*Scale-up*	
Status quo in urban Uttar Pradesh	---	5.84	$254.02	NA	---
Integrated packages of services: family planning, safe abortion, SBA, intrapartum care, transport, EmOC					
Upgrade 1 with 60.2% family planning & 50% safe abortion	18.7%	518	$243.12	$249.27	CS
Upgrade 3 with 67.9% family planning & 75% safe abortion	54.5%	341	$221.87	259.52	100
Status quo in rural Uttar Pradesh	---	633	$294.10	NA	---
Increased access to family planning					
Upgrade 1 with 45.7% family planning & 50% safe abortion	22.4%	527	$272.49	$292.05	CS
Upgrade 3 with 57.6% family planning & 75% safe abortion	58.8%	339	$235.25	$317.93	170
Status quo in Rajasthan	---	528	$242.12		---
Increased access to family planning					
Upgrade 1 with 50.9% family planning & 50% safe abortion	17.7%	459	$225.46	$241.25	CS
Upgrade 3 with 58.2% family planning & 75% safe abortion	52.6%	303	$202.81	$286.92	480

a ICER = incremental cost-effectiveness ratio; YLS = years of life saved; MMR = maternal mortality ratio; CS = cost saving; TFR = total fertility rate.

b Upgrade 1 in urban Uttar Pradesh: 45% facility births; 25% SBA (home births); transport from home (50%), primary-level health centre (60%), bEmOC (70%); recognition of referral need at home (40%), primary-level health centre (60%); availability and quality of EmOC (70%)

c Upgrade 3 in urban Uttar Pradesh: 75% facility births; 50% SBA (home births); transport from home (70%), primary-level health centre (80%), bEmOC (90%); recognition of referral need at home (75%), primary-level health centre (90%); availability and quality of EmOC (90%)

d Upgrade 1 in rural Uttar Pradesh: 40% facility births; 40% SBA (home births); transport from home (25%), primary-level health centre (60%), bEmOC (75%); recognition of referral need at home (50%), primary-level health centre (75%); availability and quality of EmOC (70%)

e Upgrade 3 in rural Uttar Pradesh: 75% facility births; 50% SBA (home births); transport from home (45%), primary-level health centre (80%), bEmOC (90%); recognition of referral need at home (75%), primary-level health centre (90%); availability and quality of EmOC (90%)

f Upgrade 1 in Rajasthan: 45% facility births; 25% SBA (home births); transport from home (50%), primary-level health centre (60%), bEmOC (70%); recognition of referral need at home (40%), primary-level health centre (60%); availability and quality of EmOC (70%)

g Upgrade 3 in Rajasthan: 75% facility births; 50% SBA (home births); transport from home (70%), primary-level health centre (80%), bEmOC (90%); recognition of referral need at home (75%), primary-level health centre (90%); availability and quality of EmOC (90%)

Supplemental Results: Family Planning

Sensitivity analyses that reduce unmet need for spacing versus limiting, without targeting specific age groups, had minimal overall policy impact when varied across plausible ranges. In contrast, focused family planning interventions that increase uptake of contraception in younger women (and therefore weighted towards spacing) had a greater impact than those focused on uptake in older women (and therefore weighted towards limiting). The effect of focused family planning interventions that shift contraceptive choices with higher failure rates to more effective options had minimal effect on the overarching policy choices in large part because of the small proportion of women affected. We also conducted an analysis exploring family options for reducing the unmet need for limiting, spacing and shifting to more effective contraception for women who experienced pregnancy (i.e., failure) in Uttar Pradesh and Rajasthan.

Table 2. Enhanced family planning in Uttar Pradesh and Rajasthan [a]

Strategy	Reduction in maternal mortality	TFR	Average lifetime costs	ICER ($/YLS)
Status quo in urban Uttar Pradesh	---	3.00	$254.02	---
Increased access to family planning				
s 25% reduction in unmet need (60.2%)	9.4%	2.73	$248.74	CS
100% reduction in unmet need (71.8%)	30.3%	2.09	$213.90	CS
Status quo in rural Uttar Pradesh	---	4.15	$294.10	---
Increased access to family planning				
25% reduction in unmet need (60.2%)	8.2%	3.81	$274.50	CS
100% reduction in unmet need (71.8%)	34.4%	2.72	$215.20	CS
Status quo in Rajasthan	---	3.24	$242.12	---
Increased access to family planning				
25% reduction in unmet need (60.2%)	6.3%	3.04	$229.73	CS
100% reduction in unmet need (71.8%)	25.0%	2.42	$192.89	CS

a ICER = incremental cost-effectiveness ratio; YLS = years of life saved; MMR = maternal mortality ratio; CS = cost saving; TFR = total fertility rate. Reduction in direct causes of maternal mortality, including abortion-related complications, postpartum hemorrhage, hypertensive disorders of pregnancy, sepsis and obstructed labor. Anemia was assumed to be responsible for 15% of deaths, exerting mortality impact on direct causes through severity of PPH, sepsis and unsafe abortion.

Supplemental Results: Antenatal Care Sensitivity Analysis

Table 3. Incremental benefit compared with status quo[b]		
	Reduction in Maternal Mortality, %	*Cost-effectiveness Ratio ($/YLS)*
Increase in antenatal care only with no other intervention (42.8%)[c]		
25% increase (53.5%)	.6%	
50% increase (64.2%)	1.2%	>$5,000
75% increase (85.6%)	1.6%	
Increase in antenatal care plus effective treatment with full compliance for anemia[d]		
25% increase (53.5%)	1.5%	
50% increase (64.2%)	3.6%	$1,500-$2,100
75% increase (85.6%)	5.5%	
Increase in antenatal care (42.8%) linked to increase in facility birth/plan to access EmOC[e]		
25% increase (53.5%), 50% facility births	6.3%	
50% increase (64.2%), 60% facility births	10.8%	$640-$790
75% increase (85.6%), 70% facility births	17.8%	

a TBA = traditional birth attendant; SBA = skilled birth attendant; PPH = postpartum hemorrhage; YLS = years of life saved; bEmOC = basic emergency obstetrical care

b Status quo: 31.1% facility births (70% in facility with incomplete bEmOC capabilities); 68.9% home births; 11.6% SBA (home births); transport from home (24.4%), primary-level health centre (48.8%), bEmOC (61%); recognition of referral need at home (20%), primary-level health centre (40%); availability and quality of EmOC (42.5%).

c Antenatal care only assumes no increased services as part of antenatal care, and no linkages with enhanced interventions that might be related.

d Antenatal care with effective treatment for anemia has a greater effect, with up to 5.5% mortality reduction independent of any other interventions.

e Antenatal care that then increases the likelihood of a facility-based delivery (30% in bEmOC capable facility and 70% in health centre) more effective and cost-effective, at 50% to 75% the per capita GDP.

Supplemental Results: Sensitivity Analysis of Distribution of Routine Births in Facilities

Health Centre and Birthing Centres, bEmOC and cEmOC

While the main analysis in the paper presents a number of scenarios that incrementally shift women from home deliveries to facility-based deliveries, here we conduct a sensitivity analysis varying assumptions with respect to the distribution of facilities. The example provided below assumes that 60% of deliveries are facility-based outside the home. In **Table 4A** we varied assumptions (depicted in yellow shading) about the proportion in birthing centres as compared to EmOC facilities while holding the distribution within EmOC facilities constant (90% bEmOC and 10% cEmOC with referral to cEmOC when necessary. We assumed 70% effective referral from the birthing centre and 80% effective referral from bEmOC to cEmOC based on the availability of transport. Results are shown (pink shading) below. Largely due to imperfect availability of prompt transport and accurate referral from the health centre, mortality is reduced most when deliveries occur in EmOC facilities although by only a small margin. Accordingly, given the cost advantage of birthing centres, the incremental cost-effectiveness ratios for Scenario 1 are low (i.e., very attractive), and in fact less than 25% of the per capita GDP. Cost-effectiveness ratios increase from $240 to $810 if one restricts routine deliveries to EmOC facilities only. Of note, provided the EmOC facilities are mainly bEmOC, the ratio is still less than the per capita GDP and thus this would be considered cost-effective

Table 4A. Impact of distribution among facilities (rural India) [a]

Varying proportion between health centre/birthing centre and EmOC

	Scenario 1[b]<br>70% HC<br>30% EmOC	Scenario 2<br>50% HC<br>50% EmOC	Scenario 3<br>25% HC<br>75% EmOC	Scenario 4[c]<br>0%HC<br>100% EmOC
Facility Delivery (60%), Transport HC to EmOC (70%), Transport bEmOC to cEmOC (80%)				
HC or BC	70%	50%	25%	0%
bEmOC	27%	45%	67.5%	90%
cEmOC	3%	5%	7.5%	10%
Mortality	1051	1034	1012	977
Cost-effectiveness ratio [d]	$240	$640	$790	$810

a. HC = health centre, BC = birthing centre. bEmOC = basic emergency obstetric care, cEmOC = comprehensive emergency obstetric care. HC and BC used interchangeably and assume SBA, clean delivery, expectant management, but lack all 6 signal functions. In this particular analysis we also assume SBA-administered misoprostol in birthing centres/health centres.
b. Scenario 1: Assumes facility-based delivery 70% in HC/BC and 30% in EmOC facilities.
c. Scenario 4: Assumes facility-based delivery all occurs in EmOC facilities.
d. Incremental cost-effectiveness ratio calculated as the additional costs divided by the additional health effects.

In **Table 4B** we varied assumptions about the proportion of routine deliveries in bEmOC versus cEmOC. In this analysis we assumed that transport/succesful referral was available for 95% of women delivering in bEmOC who required it. We assumed 70% effective referral from the birthing centre, as we did above. Results for shifting the base case distribution (90% bEmOC and 10% cEmOC, as shown in blue) to a distribution where the majority of routine deliveries occur in cEmOC, are shown in green. As expected, this change results in cost-effectiveness ratios that are clearly well beyond traditionally acceptable thresholds for cost-effectiveness, and they became less

attractive as one shifts a greater number of births to EmOC (e.g., right side of the table, scenario 4). In fact, this is one of the least efficient strategies that we identified.

Table 4B. Varying proportion between basic and comprehensive EmOC (rural India) [a]

	Scenario 1<br>70% HC<br>30% EmOC	Scenario 2<br>50% HC<br>50% EmOC	Scenario 3<br>25% HC<br>75% EmOC	Scenario 4<br>0%HC<br>100% EmOC
Facility Delivery (60%), Transport HC to EmOC (70%), Transport bEmOC to cEmOC (95%)				
Cost-effectiveness ratios				
90% bEmOC<br>10% cEmOC	**$220**	**$530**	**$630**	**$640**
10% bEmOC<br>90% cEmOC	**$8,300**[b]	**$13,800**[b]	**$20,700**[b]	**$27,700**[b]

a. HC = health centre, BC = birthing centre. bEmOC = basic emergency obstetric care, cEmOC = comprehensive emergency obstetric care. HC and BC used interchangeably and assume SBA, clean delivery, expectant management, but lack all 6 signal functions. In this particular analysis we also assume SBA-administered misoprostol in birthing centres/health centres.
b. Incremental cost-effectiveness ratio calculated as the additional costs divided by the additional health effects, in comparison to the base case 90%bEmOC and 10%cEmOC.

Supplemental Results Table 5. Sensitivity Analysis: Comparison of Community-Based Intervention Analysis (Pagel)

We compared our model projected benefits of a community based intervention where antibiotics for sepsis, and misoprostol for PPH were added to births at health centers, and at home when skilled attendants were present to those reported in Pagel et al (2009). We approximated the coverage levels of antibiotics for sepsis and misoprostol distribution (and approximate percentage of facility births) by applying the intervention to our model of rural India. Strategies were similar but not identical as our model includes all causes of maternal mortality, multiple facility-levels, mild, moderate and severe complications, and explicit consideration of the three delays.

	Coverage range (Pagel et al. 2009)		Rural India (Goldie et al) Selected Base Case Strategies		Rural India (Goldie et al) Community Intervention Analysis
**Probability that a woman with sepsis obtains antibiotics [b]**					
Package one [c]	25% (22–30)	90% (85–95)	Status quo	24%	42.8%
Package two [c]	40% (30–50)	95% (90–95)	Upgrade 2	34.6%	75.5%
Package three [c]	70% (35–85)	99% (95–98)	Upgrade 3	63.5%	87.9%
**Coverage of misoprostol distribution [d]**					
Package one [e]	N/A	N/A	Status quo		42.8%
Package two	81% (68–86)	87% (73–92)	Upgrade 2	34.6%	75.5%
Package three	92% (90–98)	100% (97–100)	Upgrade 3	63.5%	87.9%

a In Pagel et al, there is only 1 level of facility; woman would only receive oxytocin if she gave birth in a facility. In our model there are multiple levels of facilities, primary, secondary and tertiary and oxytocin is available in secondary and tertiary facilities. In the base case, birthing centres are intended to be interim primary-level facilities staffed by SBA but not with full bEmOC capabilities. Our analysis of misoprostol assumes use by SBA for both home deliveries and for birthing centre deliveries.

b In Pagel et al, if a woman develops an infection after delivery she could obtain antibiotics either from a facility or privately. In our model a woman would only receive antibiotics if she delivers in or is successfully referred to a facility capable of providing intra-partum care. In both Pagel et al and our model, the effectiveness of antibiotics for reducing sepsis related deaths in the community setting was 85%.

c Package 1 strengthens facilities, ensuring that more facilities have available oxytocin and antibiotics, package 2 combines package 1 with distribution of misoprostol to women attending outreach antenatal care appointments (provided drug was available) and distribution of antibiotics by CHW to women with signs of postnatal infection, and package 3 enhances package two with additional misoprostol and antibiotics via female volunteers in villages.

d In Pagel et al, the probability that a woman would take misoprostol if it was given outside of a facility was 85%, and the probability facility would have uterotonic drugs in packages 1-3 was 95%. We assume that facility based availability of uterotonics is a function of quality and availability of services which were varied across upgrades from 42% to 95%. The relative risk reduction for severe PPH with misoprostol was 0.61 in Pagel et al, and was varied from 0.50 - 0.61 in our model.

Pagel et al (2009) reported annual prevented deaths from PPH and sepsis in both percentage reductions and absolute cases for Malawi and Sub-Saharan Africa. Our model projects the number of deaths that would be averted over the lifetime of a birth cohort of 100,000 girls, as well as the reductions achieved from these interventions. Further, our model includes competing risk from all other causes of maternal mortality, and includes both severe and moderate or less PPH. Correcting for this, we show below the results when expressed in the format projected from Pagel et al (2009). While the absolute numbers are not directly comparable given population differences, the magnitude of mortality risk reduction for these specific community-based interventions is comparable. Interestingly, the results for the lowest three quintiles reported in Pagel et al (2009) most closely approximate our results in rural India.

Comparison of Pagel et al (2009) results (5 quintiles) and Goldie et al (Rural India)

	Pagel et al.(2009)		Goldie et al. (Rural India only)	
	Malawi (Annual mortality)	Sub-Saharan Africa (Annual mortality)	Rural India (per 100,000 females)	Reduction applied to annual population level estimate
PPH/sepsis deaths	2,860	182,000	962	49,374 [Khan 2006]
Reduction in PPH/sepsis related mortality				
Package 1	210 (7%)	21,300 (12%)	128 (13%)	6,586
Package 2	720 (25%)	43,800 (24%)	197 (27%)	13,331
Package 3	1,020 (36%)	59,000 (32%)	159 (28%)	13,825

Comparison of Pagel et al (2009) results (5 quintiles, lowest 3 quintiles) and Goldie et al (Rural India)

Reduction in PPH/sepsis related mortality

	Malawi		Sub-Saharan Africa		Rural India
	5 quintiles	*lowest 3 quintiles*	*5 quintiles*	*lowest 3 quintiles*	
Package 1	7%	4%	12%	7%	13%
Package 2	25%	19%	24%	17%	27%
Package 3	36%	28%	32%	25%	28%

Supplemental Results Table 6: Benefits and cost-effectiveness of improved intrapartum care alone in India [a]

Facility birth (%)	Transport-home[d] (%)	Transport-facility[d] (%)	Quality of care[e] (%)		Decrease in maternal deaths	MMR deaths per 100,000 live births	Maternal deaths as % deaths ages 15-45	Lifetime risk of maternal death	Lifetime costs	Cost-Effectiveness [f]	
										ICER ($/YLS)	ICER (% per capita GDP)
				**Rural India** [b]		*520*	*16.4%*	*1 in 65*	*$218.38*	---	
45	50	65	70	Upgrade 1 alone	9.3%	470	15.1%	1 in 71	$238.64	490	46%
60	60	75	80	Upgrade 2 alone	19.9%	414	13.6%	1 in 81	$257.06	520	49%
75	70	85	90	Upgrade 3 alone	35.8%	332	11.2%	1 in 101	$310.92	970	91%
80	75	95	95	Upgrade 4 alone	59.6%	207	7.3%	1 in 160	$341.35	1,060	99%
				**Urban India** [c]		*407*	*9.6%*	*1 in 119*	*$184.00*	---	
75	60	85	70	Upgrade 1 alone	8.2%	374	8.9%	1 in 129	$194.96	200	19%
80	70	90	80	Upgrade 2 alone	20.3%	323	7.8%	1 in 149	$203.06	500	47%
90	80	92.5	90	Upgrade 3 alone	37.5%	255	6.2%	1 in 190	$232.74	770	72%
95	85	95	95	Upgrade 4 alone	60.9%	158	4.0%	1 in 304	$242.54	990	93%

Supplemental Results Table 6: Benefits and cost-effectiveness of improved intrapartum care alone in India [a]

a ICER = incremental cost-effectiveness ratio; YLS = years of life saved; MMR = maternal mortality ratio; SBA = skilled birth attendant; EmOC = emergency obstetric care; bEmOC = basic emergency obstetric care facility; cEmOC = comprehensive emergency obstetric care facility; Reduction in direct causes of maternal mortality, including abortion-related complications, postpartum hemorrhage, hypertensive disorders, sepsis and obstructed labor.

b Status quo (Rural India): 31.1% facility births; 11.6% SBA (home births); transport from home (24.4%), primary-level health centre (48.8%), bEmOC (61%); recognition of referral need at home (20%), primary-level health centre (40%); availability and quality of EmOC (42.5%).

c Status quo (Urban India): 69.4% facility births; 19% SBA (home births); transport from home (44%), primary-level health centre (69%), bEmOC (81%); recognition of referral need at home (20%), primary-level health centre (40%); availability and quality of EmOC (67.5%).

d Transport encompasses the expedient availability of means of transport (e.g., vehicle, cart), fuel (if needed), driver, and interim attendant care. Facility transport represents a weighted average of transport availability from a health centre or birthing center to an EmOC facility and from a bEmOC facility to a cEmOC if indicated. Accuracy of referral need recognition at home and in health centre with SBA increase, on average, to 60%, 75%, 90%, and 95% (not shown) with upgrade 1, 2, 3 and 4 in both rural and urban India.

e Quality refers to the availability and quality of services at EmOC facilities, including adequate staffing and supplies, expedient attention (e.g., without delay to collect fees or requirement for family to bring supplies), and evidence-based clinical practices.

f Stepwise improvements in maternal health services are assumed to occur in consecutive phases (e.g., first upgrade 1, then upgrade 2, etc.). Therefore, the incremental cost-effectiveness ratio (US dollars per YLS) for each ‘upgrade’ is calculated as the difference in costs relative to the difference in effects, compared with the preceding next best strategy. Cost-effectiveness ratios are also expressed as percent of the per capita GDP (U.S. $1,068), shown in the farthest right column, as interventions with cost-effectiveness ratios of less than the per capita GDP are considered very cost-effective according to criteria proposed by the Commission on Macroeconomics and Health.

Part V: References

AbouZahr C, Wardlaw T (2004) Maternal mortality in 2000: estimates developed by WHO, UNICEF and UNFPA. Geneva: World Health Organization. Available: http://www.who.int/reproductivehealth/publications/monitoring/9241562706/en/index.html. Accessed 23 August 2009.

Adam T, Lim SS, Mehta S, Bhutta ZA, Fogstad H, et al. (2005) Cost effectiveness analysis of strategies for maternal and neonatal health in developing countries. BMJ 331(7525):1107.

Ahman E, Dolea C, Shah I (2006) The global burden of unsafe abortion in the year 2000, Geneva: World Health Organization, Available: http://www.who.int/healthinfo/statistics/bod_abortions.pdf. Accessed 4 August 2008.

Ahman E, Shah I (2004) Unsafe abortion: global and regional estimates of unsafe abortion and associated mortality in 2000 (4th edn). Geneva: World Health Organization. Available: http://www.who.int/reproductivehealth/publications/unsafe_abortion/9241591803/en/index.html. Accessed 24 August 2009.

Ahman E, Shah I (2007) Unsafe abortion: global and regional estimates of unsafe abortion and associated mortality in 2003 (5th edn). Geneva: World Health Organization. Available: http://www.who.int/reproductivehealth/publications/unsafe_abortion/9789241596121/en/index.html. Accessed 24 August 2009.

Alfirevic Z, Blum J, Walraven G, Weeks A, Winikoff B. Prevention of postpartum hemorrhage with misoprostol. Int J Gynaecol Obstet. 2007 Dec; 99 Suppl 2: S198-201

AMDD Working Group on Indicators (2002) Program note: Using UN process indicators to assess needs in emergency obstetric services: Bhutan, Cameroon and Rajasthan, India. Int J Gynaecol Obstet 77(3):277-84.

Amowitz LL, Reis C, Iacopino V (2002) Maternal mortality in Herat Province, Afghanistan, in 2002: an indicator of women's human rights. JAMA 288(10):1284-91.

Automobile India. Auto accident statistics. Available: http://www.automobileindia.com/automobile-industry/auto-accident.html Accessed 25 August 2009.

Bakr AF, Karkour T (2005) Effect of predelivery vaginal antisepsis on maternal and neonatal morbidity and mortality in Egypt. J Womens Health 14(6):496-501

Bang AT, Baitule SB, Reddy HM, Deshmukh MD, Bang RA. (2005a). Low birth weight and preterm neonates: can they be managed at home by mother and a trained village health worker? Journal of Perinatology 25:S72-S81.

Bang AT, Bang RA, Baitule SB, Reddy HM, Deshmukh MD. (2005b) Management of birth asphyxia in home deliveries in rural Gadchiroli: the effect of two types of birth attendants and of resuscitating with mouth-to-mouth, tube-mask or bag-mask. J Perinatol 25 Suppl 1:S82-91.

Bang RA, Bang AT, Reddy MH, Deshmukh MD, Baitule SB, et al. (2004) Maternal morbidity during labour and the puerperium in rural homes and the need for medical attention: A prospective observational study in Gadchiroli, India. BJOG 111(3): 231-8.

Baqui A, Williams EK, Rosecrans AM, Agrawal PK, Ahmed S, Darmstadt GL, Kumar V, Kiran U, Panwar D, Ahuja RC, Srivastava VK, Black RE, Santosham M. (2008) Impact of an integrated nutrition and health programme on neonatal mortality in rural northern India. Bull World Health Organ; 86(10):796-804,

Bartlett LA, Mawji S, Whitehead S, Crouse C, Dalil S, et al. (2005) Where giving birth is a forecast of death: maternal mortality in four districts of Afghanistan, 1999-2002. Lancet 365(9462):864-70.

Berer M (2004) National laws and unsafe abortion: the parameters of change, Reprod Health Matters 12:1–8.

Bhat PN, Navaneetham K, Rajan SI. Maternal mortality in India: estimates from a regression model. *Stud Fam Plann* 1995;26:217-32.

Bhat R, Mavalankar DV, Singh PV, Singh N. (2009) Maternal healthcare financing: Gujarat's Chiranjeevi Scheme and Its Beneficiaries. J Health Popul Nutr 27(2):249-258.

Bhatia J, Cleland J (2004) Health care of female outpatients in South-Central India: comparing public and private sector provision. Health Policy Plann 19(6):402–9.

Biswas AB, Das DK, Misra R, Roy RN, Ghosh D, et al. (2005) Availability and use of emergency obstetric care services in four districts of West Bengal, India. J Health Popul Nutr 23(3):266-74

Borghi J, Ensor T, Somanathan A, Lissner C, Mills A, on behalf of The Lancet Maternal Survival Series steering group (2006) Mobilising financial resources for maternal health. Lancet 368(9545):1457-65

Borghi J, Hanson K, Acquah CA, Ekanmian G, Filippi V, Ronsmans C, Brugha R, Browne E, Alihonou E (2003) Costs of near-miss obstetric complications for women and their families in Benin and Ghana. Health Policy Plann. 18(4):383-90.

Brabin BJ, Hakimi M, Pelletier D (2001) An analysis of anemia and pregnancy-related maternal mortality. J Nutr 131(2 Suppl 2): 604S-614S.

Cahuana-Hurtado L, Sosa-Rubi S, Bertozzi S (2004) The Application of the Mother Baby Package Reproductive Health Costing Spreadsheet in Morelos: National Institute of Public Health, Division of Health Economics and Policy, Mexico.

Campbell OM, Graham WJ, Lancet Maternal Survival Series steering group (2006) Strategies for reducing maternal mortality: getting on with what works. Lancet 368(9543):1284-99.

Carbonell JL, Varela L, Velazco A, Cabezas E, Fernandez C, Sanchez C (1998) Oral methotrexate and vaginal misoprostol for early abortion. Contraception 57(2):83–8.

Carroli et al. (2008) Epidemiology of postpartum haemorrhage: a systematic review Best Practice & Research Clinical Obstetrics & Gynaecology, Volume 22, Issue 6, Pages 999-1012

Carroli G, Villar J, Piaggio G, Khan-Neelofur D, Gulmezoglu M, et al. (2001) WHO systematic review of randomized controlled trials of routine antenatal care. Lancet 357(9268):1565-70.

CEHAT (Centre for Enquiry into Health and Allied Themes) (2001) Unpublished studies relating to abortion in India. Available: http://www.cehat.org/aap.html. Accessed 7 May 2009.

Chandhiok N, Dhillon BS, Datey S, Mathur A, Saxena NC (2006) Oral misoprostol for prevention of postpartum hemorrhage by paramedical workers in India. Int J Gyneacol Obstet 92(2):170–5

Cotter AM, Ness A, Tolosa JE. Prophylactic oxytocin for the third stage of labour. Cochrane Database of Systematic Reviews 2001, Issue 4. Art. No.: CD001808. DOI: 10.1002/14651858.CD001808

Coyaji K, Elul B, Krishna U, Otiv S, Ambardekar S, Bopardikar A, Raote V, Ellertson C, Winikoff B 2002 Mifepristone-misoprostol abortion: a trial in rural and urban Maharashtra, India. Contraception 66: 33-40.

Creinin M, Blumenthal P, Shulman L (2006) Mortality associated with mifepristone-misoprostol medical abortion. Med Gen Med 8(2):26.

Crowther C, Hiller J, Doyle L (2002) Magnesium sulphate for preventing preterm birth in threatened preterm labour. Cochrane Database Syst Rev 4:CD001060.

Danel I, Rivera A (2003) Chapter 3: Honduras, 1990-1997. In Koblinsky M, editor. Reducing maternal mortality: learning from Bolivia, China, Egypt, Honduras, Indonesia, Jamaica, and Zimbabwe. Washington DC: The International Bank for Reconstruction and Development/The World Bank.

Datar A, Mukherji A, Sood N (2007) Health infrastructure and immunization coverage in rural India. Indian J Med Res. 125(1):31–42.

Derman RJ, Kodkany BS, Goudar SS, Geller SE, Naik VA, Bellad M, et al. (2006) Oral misoprostol in preventing postpartum haemorrhage in resource-poor communities: a randomised controlled trial. Lancet 368(9543):1248–53.

Deshpande K, Shankar R, Diwan V, Lonnroth K, Mahadik VK, Chandorkar RK (2004) Spatial pattern of private health care provision in Ujjain, India: a provider survey processed and analysed with a Geographical Information System. Health Policy 68(2):211–22.

Dolea C, AbouZahr C, Stein C (2003a) Global burden of maternal hemorrhage in the year 2000. Geneva, Switzerland: World Health Organization. Available: http://www.who.int/healthinfo/statistics/bod_maternalhaemorrhage.pdf. Accessed 23 August 2009.

Dolea C, Stein C (2003b) Global burden of maternal sepsis in the year 2000. Geneva, Switzerland: World Health Organization. Available: http://www.who.int/healthinfo/statistics/bod_maternalsepsis.pdf. Accessed 23 August 2009.

Dolea C, AbouZahr C (2003c) Global burden of obstructed labor in the year 2000. Geneva, Switzerland: World Health Organization. Available: http://www.who.int/healthinfo/statistics/bod_obstructedlabour.pdf. Accessed 23 August 2009.

Dolea C, AbouZahr C (2003d) Global burden of hypertensive disorders of pregnancy in the year 2000. Geneva, Switzerland: World Health Organization. Available: http://www.who.int/healthinfo/statistics/bod_hypertensivedisordersofpregnancy.pdf. Accessed 23 August 2009.

Duggal R (2004) The political economy of abortion in India: cost and expenditure patterns. Reprod Health Matters. 12(24 Suppl):130-7.

Duley L, Gülmezoglu A, Henderson-Smart D (2003) Magnesium sulphate and other anticonvulsants for women with eclampsia. Cochrane Database Syst Rev 2:CD000025.

Duley L, Henderson-Smart DJ, Walker GJA (2009) Interventions for treating pre-eclampsia and its consequences: generic protocol. Cochrane Database System Rev 4:CD007756

Edelman DA, Brenner WE, Berger GS (1974) The effectiveness and complications of abortion by dilatation and vacuum aspiration versus dilatation and rigid metal curettage, Am J Obstet Gynecol 119(4):473-80.

Elbourne DR, Prendiville WJ, Carroli G, Wood J, McDonald S (2001) Prophylactic use of oxytocin in the third stage of labour Cochrane Database System Rev 4:CD001808

Faúndes A, Fiala C, Tang OS, Velasco A (2007) Misoprostol for the termination of pregnancy up to 12 completed weeks of pregnancy, Int J Gynaecol Obstet, 99(Suppl 2):S172-7.

French LM (2003) Prevention and treatment of postpartum endometritis. Curr Womens Health Rep 4:274-9.

French LM, Smaill FM (2004) Antibiotic regimens for endometritis after delivery. Cochrane Database Syst Rev 4:CD001067.

Ganatra B (2006) Unsafe abortion in South and South-East Asia: a review of the evidence. In: Warriner IK, Shah IH, editors. Preventing unsafe abortion and its consequences: priorities for research and action, New York: Guttmacher Institute. pp. 151–186.

Ganatra B, Hirve S (2002) Induced abortions among adolescent women in rural Maharashtra, India, Reprod Health Matters 10:76–85.

Ganatra BR, Coyaji KJ, Rao VN (1998) Too far, too little, too late: a community-based case-control study of maternal mortality in rural west Maharashtra, India. Bull World Health Organ. 76(6):591-8.

Geelhoed D, Visser L, Agordzo P, Asare K, van Leeuwen JS, van Roosmalen J. Active versus expectant management of the third stage of labor in rural Ghana. Acta Obstet Gynecol Scand 2002; 81: 172–173.

Goodburn EA, Chowdhury M, Gazi R, Marshall T, Graham W (2000) Training traditional birth attendants in clean delivery does not prevent postpartum infection. Health Policy Plan 15(4):394-9

Government of India (2005a) Reproductive and Child Health Programme Document (RCH II – Document 1): The Reproductive and Child Health II Programme Documentation. New Delhi, India: Government of India.

Government of India (2005b) Reproductive and Child Health Programme Document (RCH II – Document 2): The principles and evidence base for State RCH II Programme Implementation Plans (PIPs). New Delhi, India: Government of India. Available: http://www.whoindia.org/LinkFiles/Child_Health_in_India_PIP_Doc_Chapter01.pdf. Accessed 21 August 2009.

Government of India, Department of Road Transport and Highways (2008b) Annual Report. New Delhi, India: Government of India. Available: http://morth.nic.in/writereaddata/sublink2images/AnnualReport2007_083953330780.pdf. Accessed 25 August 2009.

Government of India, Ministry of Health and Family Welfare, Department of Family Welfare (2005c) National rural healthcare mission. New Delhi, India: Government of India. Available: http://mohfw.nic.in/NRHM/Documents/Mission_Document.pdf. Accessed 21 August 2009.

Government of India, Ministry of Rural Development (2009) Annual Report 2007-2008. Available: www.rural.nic.in. Accessed 1 September 2009.

Government of India. (2008a) Bulletin on rural health statistics in India. Rural Health System in India. Available: http://www.mohfw.nic.in/Bulletin%20on%20RHS%20-%20March,%202008%20-%20PDF%20Version/Rural%20Health%20Care%20System%20in%20India.pdf. Accessed 1 September 2009

Graham WJ, Cairns J, Bhattacharya S, Bullough CHW, Quayyum Z, et al. (2006) Chapter 26: Maternal and perinatal conditions. In: Jamison DT, Breman JG, Measham AR, Alleyne G, Claeson M, et al., editors. Disease Control Priorities in Developing Countries (2nd Edition). New York: Oxford University Press. pp. 499-530. Available: http://www.dcp2.org/pubs/DCP/26/. Accessed 23 August 2009.

Grimes DA (2005) Risks of mifepristone abortion in context. Contraception 71(3):161.

Gulmezoglu AM, Villar J, Ngoc NT, Piaggio G, Carroli G, et al. (2001) WHO multicentre randomised trial of misoprostol in the management of the third stage of labour. Lancet 358(9283):689-95.

Gülmezoglu. Prostaglandins for preventing postpartum haemorrhage. Cochrane Database of Systematic Reviews 2007, Issue 3. Art No CD000494. DOI: 10.1002/14651858.CD000494.pub3.

Gupta N, Kumar S, Saxena NC, Nandan D, Saxena BN (2006) Maternal mortality in seven districts of Uttar Pradesh--an ICMR task force study. Indian J Public Health. 2006 Jul-Sep;50(3):173-8.

Haider BA, Bhutta ZA (2009) Community-based intervention package for preventing maternal morbidity and mortality and improving neonatal outcomes. Cochrane Database System Rev 2:CD007754.

Harlap S, Shiono PH, Ramcharan S (1980) A life table of spontaneous abortions and the effects of age, parity and other variables. In: Porter IH, Hook EB, editors. Human Embryonic and Fetal Death. New York: Academic Press. pp 145-158

Health information of India (2005) Chapter 7. Man power statistics. Available: http://www.cbhidghs.nic.in/hia2005/chap7.asp Accessed 1 September 2009.

Henshaw SK, Adewole I, Singh S, Bankole A, Oye-Adeniran B, Hussain R (2008) Severity and cost of unsafe abortion complications treated in Nigerian hospitals. Int Fam Plan Perspect 34:40-50.

Henshaw SK, Singh S, Haas T (1999) The incidence of abortion worldwide. Int Fam Plann Persp 25(Suppl):S30-8.

Hill K, Thomas K, AbouZahr C, Walker N, Say L, Inoue M, Suzuki E, Maternal Mortality Working Group (2007) Estimates of maternal mortality worldwide between 1999 and 2005: an assessment of available data. Lancet 370(9595):1311-9.

Hirve SS (2004) Abortion law, policy and services in India: a critical review. Reprod Health Matters 12:114–21.

Hofmeyr GJ, Gulmezoglu AM, Novikova N, Linder V, Ferreira S, Piaggio G (2009) Misoprostol to prevent and treat postpartum haemorrhage: a systematic review and meta-analysis of maternal deaths and dose-related effects. Bull World Health Organ 87:666-77.

Hofmeyr GJ, Gülmezoglu AM. Misoprostol for the prevention and treatment of postpartum haemorrhage. Best Practice & Research Clinical Obstetrics and Gynaecology 2008; doi:10.1016/j.bplobgyn.2008.08.005

Hofmeyr GJ, Hannah ME (2003) Planned Caesarean section for term breech delivery. Cochrane Database Syst Rev 3:CD000166.

Hofmeyr GJ, Kulier R (2000) External cephalic version for breech presentation at term. Cochrane Database Syst Rev 2: CD000083.

Hofmeyr GJ, Walraven G, Gulmezoglu AM, Maholwana B, Alfirevic Z, Villar J (2005) Misoprostol to treat postpartum haemorrhage: a systematic review. BJOG 112:547-53

Høj L, Cardoso P, Nielsen BB, Hvidman L, Nielsen J, Aaby P (2005) Effect of sublingual misoprostol on severe postpartum haemorrhage in a primary health centre in Guinea-Bissau: randomised double blind clinical trial. BMJ 331(7519):723

Hu D, Bertozzi SM, Gakidou E, Sweet S, Goldie SJ (2007) The costs, benefits, and cost-effectiveness of interventions to reduce maternal morbidity and mortality in Mexico. PLoS ONE. 2(1):e750.

Hu D, Grossman D, Levin C, Blanchard K, Goldie S (2009) Cost-effectiveness analysis of alternative first-trimester pregnancy termination strategies in Mexico City. BJOG 116:768–79

Hussein J, Fortney JA (2004) Puerperal sepsis and maternal mortality: what role can new technologies play? Int J Gynaecol Obstet 85(suppl 1):S52-61

India Facility Survey (Under Reproductive and Child Health Project), Phase II, 2003. International Institute for Population Sciences, Mumbai. Sponsored by Ministry of Health and Family Welfare, Government of India, New Delhi, 2005.

India.co.in. Distance table. Available: http://india.co.in/?q=node/47. Accessed 25 August 2009.

Institute of Medicine (IOM) (1975) Legalized abortion and the public health; report of a study by a committee of the Institute of Medicine, Washington, D.C.: National Academy of Sciences.

International Institute for Population Sciences (IIPS) (2005) Reproductive and Child Health Project. Facility Survey. DLHS-2, 2003. Available: http://www.rchiips.org/pdf/rch2/National_Facility_Report_RCH-II.pdf. Accessed 1 September 2009

International Institute for Population Sciences (IIPS) (2006) Reproductive and Child Health Project. District Level Household Survey. DLHS-2. 2002-2004. Available: http://www.rchiips.org/pdf/rch2/National_Report_RCH-II.pdf. Accessed 1 September 2009.

International Institute for Population Sciences (IIPS) and ORC Macro (2000) National Family Health Survey (NFHS-2), 1998–99: India. Mumbai: IIPS. Available: http://www.nfhsindia.org/pub_nfhs-2.html. Accessed 23 August 2009.

International Institute for Population Sciences (IIPS) and ORC Macro (2007) National Family Health Survey (NFHS-3), 2005-06: India. Mumbai: IIPS. Available: http://www.nfhsindia.org/nfhs3_national_report.html. Accessed 23 August 2009.

International Institute for Population Sciences (IIPS) and ORC Macro (2007) National Family Health Survey (NFHS-3), 2005-06: India. Fertility and fertility preferences, Chapter 4. Mumbai: IIPS. pp 77-109

International Institute for Population Sciences (IIPS) and ORC Macro (2007) National Family Health Survey (NFHS-3), 2005-06: India. Family planning, Chapter 5. Mumbai: IIPS. pp 111-60

International Institute for Population Sciences (IIPS) and ORC Macro (2007) National Family Health Survey (NFHS-3), 2005-06: India. Maternal health, Chapter 8. Mumbai: IIPS. pp 191-222

International Institute for Population Sciences (IIPS) Fact Sheet: Uttar Pradesh. Reproductive and Child Health Project. District Level Household and Facility Survey. DLHS-3. 2007-2008. Available: http://www.rchiips.org/pdf/rch3/state/Uttar-Pradesh.pdf. Accessed 1 September 2009.

International Institute for Population Sciences (IIPS) Fact Sheet: Rajasthan. Reproductive and Child Health Project. District Level Household and Facility Survey. DLHS-3. 2007-2008. Available: http://www.rchiips.org/pdf/rch3/state/Rajsthan.pdf. Accessed 1 September 2009.

International Labour Organization (ILO). Laborsta database. Available: http://laborsta.ilo.org/. Accessed 27 July 2009.

Internatonal Energy Agency (IEA) (2006) Selected indicators for India. Available: http://www.iea.org/textbase/stats/indicators.asp?COUNTRY_CODE=IN. Accessed 25 August 2009.

Ipas, *Medical abortion: making a difference in rural India and around the globe*, Chapel Hill, NC: Ipas, June 2008. [http://www.ipas.org/Library/News/News_Items/Medical_abortion_making_a_difference_in_rural_India_around_the_globe.aspx] Accessed 18 July 2008.

Iyengar K, Iyengar SD, Suhalka V, Dashora K (2009c) Pregnancy-related deaths in rural Rajasthan, India: exploring causes, context, and care-seeking through verbal autopsy J Health Popul Nutr 27(2):293-302

Iyengar SD, Iyengar K, Gupta V (2009a) Maternal health: a case study of Rajasthan. J Health Popul Nutr. 2009 Apr;27(2):271-92.

Iyengar SD, Iyengar K, Suhalka V, Agarwal K (2009b) Comparison of Domiciliary and Institutional Delivery-care Practices in Rural Rajasthan, India J Health Popul Nutr 27(2):303-12.

Jain V, Saha SC, Bagga R, Gopalan S (2004) Unsafe abortion: a neglected tragedy. Review from a tertiary care hospital in India. J Obstet Gynaecol Res 30:197-201.

Johanson RB, Menon BK (2000) Vacuum extraction versus forceps for assisted vaginal delivery. Cochrane Database Syst Rev 2:CD000224.

Johns B, Sigurbjornsdottir K, Fogstad H, Zupan J, Mathai M, et al. (2007) Estimated global resources needed to attain universal coverage of maternal and newborn health services. Bull World Health Organ 85(4):256-63.

Kashanian M, Fekrat M, Masoomi Z, Sheikh Ansari N. Comparison of active and expectant management on the duration of the third stage of labour and the amount of blood loss during the third and fourth stages of labour: a randomised controlled trial. Midwifery. *In press* (PMID: 18706744)

Katrak H (2008) Measuring the shortage of medical practitioners in rural and urban areas in developing countries: a simple framework and simulation exercise with data from India. Int J Health Plann Manage 23(2):93-105

Khan KS, Wojdyla D, Say L, Gulmezoglu AM, Van Look PF (2006) WHO analysis of causes of maternal death: a systematic review. Lancet 367(9516):1066-74.

Koblinsky M, Matthews Z, Hussein J, Mavalankar D, Mridha MK, Anwar I, Achadi E, Adjei S, Padmanabhan P, van Lerberghe W, on behalf of The Lancet Maternal Survival Series steering group. Going to scale with professional skilled care. Lancet. 2006;368(9544):1377-86

Koopmans CM, Bijlenga D, Aarnoudse JG, van Beek E, Bekedam DJ, van den Berg PP, et al. (2007) Induction of labour versus expectant monitoring in women with pregnancy-induced hypertension or mild preeclampsia at term: the HYPITAT trial. BMC Pregnancy Childbirth 7:14.

Koopmans CM, Bijlenga D, Groen H, Vijgen SM, Aarnoudse JG, Bekedam DJ, et al. (2009) Induction of labour versus expectant monitoring for gestational hypertension or mild pre-eclampsia after 36 weeks' gestation (HYPITAT): a multicentre, open-label randomized controlled trial. Lancet 374(9694):979-88.

Koonin LM, Smith JC, Ramick M (1993) Abortion surveillance--United States, 1990, MMWR CDC Surveill Summ 42(6):29-57.

Langer A, Villar J, Tell K, Kim T, Kennedy S (2008) Reducing eclampsia-related deaths—a call to action. Lancet 371(9614):705–6

Laufe LE (1977) The menstrual regulation procedure, Stud Fam Plann 8(10):253-6.

Leduc D, Senikas V, Lalonde AB, Ballerman C, Biringer A, Delaney M, et al. (2009) Active management of the third stage of labour: prevention and treatment of postpartum hemorrhage. J Obstet Gynaecol Can 31(10):980-93

Liabsuetrakul T, Choobun T, Peeyananjarassri K, Islam M (2004) Antibiotic prophylaxis for operative vaginal delivery. Cochrane Database Syst Rev 3:CD004455.

Lule E, Singh S, Chowdury SA (2007) Fertility regulation behaviors and their costs: contraception and unintended pregnancies in Africa and Eastern Europe & Central Asia. Washington, D.C.: World Bank. Available:

http://siteresources.worldbank.org/HEALTHNUTRITIONANDPOPULATION/Resources/281627-1095698140167/FertilityRegulationsFinal.pdf. Accessed 24 August 2009.

Ma S, Sood N (2008) A comparison of the health systems in China and India. Rand Corporation. Available: www.rand.org/pubs/occasional_papers/2008/RAND_OP212.pdf. Accessed 27 July 2009.

Magpie Trial Collaborative Group (2002) Do women with pre-clampsia and their babies benefit from magnesium sulphate? The Magpie Trial: a randomised placebo controlled trial. Lancet 359: 1877-90.

Magpie Trial Follow-up Study Collaborative Group (2007) The Magpie Trial: a randomised trial comparing magnesium sulphate with placebo for pre-eclampsia. Outcome for women at 2 years. BJOG. 114(3):300-9

Management Sciences for Health (MSH). International Drug Price Indicator Guide (IDPIG). Available: http://erc.msh.org/mainpage.cfm?file=1.0.htm&id=1&temptitle=Introduction&module=DMP&language=English. Accessed 27 July 2009.

Maps of India, City Distance Search Engine; Distance Calculator. Available: http://www.mapsofindia.com/distance/distance.jsp; http://www.mapsofindia.com/distances/. Accessed 25 August 2009.

Mavalankar DV, Rosenfield A (2005) Maternal mortality in resource-poor settings: policy barriers to care. Am J Public Health. 95(2):200-3.

Mavalankar DV, Vora KS, Ramani KV, Raman P, Sharma B, et al. (2009) Maternal health in Gujarat, India: a case study J Health Popul Nutr 27(2):235-48.

Menken J, Rahman MO (2006) Chapter 3: Reproductive health. In: Merson MH, Black RE, Mills AJ, editors. International Public Health: Diseases, Programs, Systems, and Policies, 2nd ed. Sudsbury, MA: Jones and Bartlett Publishers. pp. 71-125.

Mills A, Brugha R, Hanson K, McPake B (2002) What can be done about the private health sector in low-income countries? Bull World Health Organ 80(4):325–30.

Mills S, Bos E, Lule E, Ramana GNV, Bulatao R (2007) Obstetric care in poor settings in Ghana, India, and Kenya. Washington, DC: The World Bank. Available: http://www-wds.worldbank.org/servlet/main?menuPK=64187510&pagePK=64193027&piPK=64187937&theSitePK=523679&entityID=000310607_20080123112201. Accessed 27 July 2009.

Ministry of Health (2004) Annual Report. New Delhi: Ministry of Health and Family Welfare, Government of India. Available: http://mohfw.nic.in/reports%5CAnnual2004%5CAnnual%20Report%20Eng%5Ccontents.htm. Accessed 16 May 2009.

Mosha F, Winani S, Wood S, Changalucha J, Ngasalla B (2005) Evaluation of the effectiveness of a clean delivery kit intervention in preventing cord infection and puerperal sepsis among neonates and their mothers in rural Mwanza Region, Tanzania.Tanz Health Res Bull 7(30):185-8.

Murray CJ, Lopez AD (eds) (1998) Health Dimensions of Sex and Reproduction. Cambridge, MA: Harvard School of Public Health.

Murray CJL, Lopez AD (1996) Estimating causes of death: new methods and global and regional applications for 1990. In: Murray CJL, Lopez AD, editors. The global burden of disease, vol. 1 of Global burden of disease and injury series. Cambridge, MA: Harvard University Press; p. 117–200.

Murthy N, Barua A (2004) Non-medical determinants of maternal death in India. J Health Manag 6;47-61.

Neogi AJ (2007) Economic impact of unsafe abortions on the household. Global Safe Abortion Conference, October 23-24, 2007. London. Available: http://www.globalsafeabortion.org/Media/Session03/Presentations/Seminar_2_Armin_Neogi.pdf Accessed 19 August 2009.

Padmanaban P Raman PS, Mavalankar DV. (2009) Innovations and challenges in reducing maternal mortality in Tamil Nadu, India. J Health Popul Nutr 27(2):202-19.

Pagel C, Lewycka S, Colbourn T, Mwansambo C, Mequid T, Chiudzu G et al. (2009) Estimation of potential effects of improved community-based drug provision, to augment health-facility strengthening, on maternal mortality due to post-partum haemorrhage and sepsis in sub-Saharan Africa: an equity-eff ectiveness model *Lancet;* 374: 1441–48.

Pandey A, Aggarwal A, Singh P, Manchanda VK, Maiti KD (2003) Estimates of maternal mortality ratios in India and its states: a pilot study. Department of Family Welfare, Ministry of Health and Family Welfare, Government of India. Institute for Research in Medical Statistics. Indian Council of Medical Research. Ansari Nagar, New Delhi-110029.

Patel AR, Nowalk MP (2010) Expanding immunization coverage in rural India: A review of evidence for the role of community health workers. Vaccine 28(3):604-13

Pendse V (1999) Maternal deaths in an Indian hospital: a decade of (no) change? In: Berer M, Ravindran TKS, editors. Safe motherhood initiatives: critical issues. Oxford, England: Blackwell Science. pp 119-26

PMGSY (Pradhan Mantri Gram Sadak Yojana) (2006) Briefing Book. Available: http://pmgsy.nic.in/. Accessed 27 July 27 2009.

Population Reference Bureau (PRB) (2004) World Population Data Sheet. Washington, DC: Population Reference Bureau.

Prakasamma M. (2009) Maternal mortality-reduction programme in Andhra Pradesh. J Health Popul Nutr 27(2):220-34.

Prata N, Mbaruku G, Campbell M, Potts M, Vahidnia F (2005) Controlling postpartum hemorrhage after home births in Tanzania. Int J of Gynecology and Obstetrics 90:51-55.

Prata N, Sreenivas A, Vahidnia F, Potts M (2009) Saving maternal lives in resource-poor settings: facing reality. Health Policy 89(2):131-48.

Prendiville WJ, Elbourne D, McDonald S (2000) Active versus expectant management in the third stage of labour. Cochrane Database System Rev 3. CD000007

Prendiville WJ, Elbourne D, McDonald S (2009) Active versus expectant management in the third stage of labour. Cochrane Database System Rev 3. CD000007

Ram F, Singh A (2006) Is antenatal care effective in improving maternal health in rural Uttar Pradesh? Evidence from a district level household survey. J Biosoc Sci 38(4):433-48.

Ramachandar L, Pelto PJ (2002) The role of village health nurses in mediating abortions in rural Tamil Nadu, India. Reprod Health Matters 10:64-75

Ramachandar L, Pelto PJ (2004) Abortion providers and safety of abortion: a community-based study in a rural district of Tamil Nadu, India. Reprod Health Matters 12:138-46

Ramachandar L, Pelto PJ (2005) Medical abortion in rural Tamil Nadu, South India: a quiet transformation. Reprod Health Matters 13:54-64

Reeves MF, Creinin MD (2006) Mortality in perspective: mifepristone-misoprostol medical abortion. Johns Hopkins Advanced Studies in Medicine. 6(10):428-30. Available:

http://www.jhasim.com/files/articlefiles/pdf/ASIM%20Master_6_10pOp-Ed.pdf. Accessed 30 July 2008.

Registrar General of India. Sample registration system. New Delhi: Registrar General of India, 1998:20-9. (Analytical studies report no. 1).

Registrar General, India (2006) Maternal mortality in India: 1997-2003; trends, causes and risk factors (Sample Registration System). New Delhi, India. Available: http://www.mp.gov.in/health/Maternal_Mortality_in_India_1997-2003.pdf. Accessed 22 August 2009.

Ronsmans C, Graham WJ, on behalf of The Lancet Maternal Survival Series steering group. Maternal mortality: who, when, where, and why. Lancet. 2006;368(9542):1189-1200.

Sanghvi H, Wiknjosastro G, Chanpong G, Fishel J, Ahmed S, Zulkarnain M (2004) Prevention of postpartum hemorrhage study. West Java, Indonesia.Report. USAID/JHPIEGO/MNH Program. Available: http://www.jhpiego.jhu.edu/resources/pubs/mnh/pphjavastudy.pdf. Accessed 12 December 2009.

Sawhney H, Aggarwal N, Biswas R, Vasishta K, Gopalan S (2000) Maternal mortality associated with eclampsia and severe preeclampsia of pregnancy. J Obstet Gynaecol Res 26:351-6.

Schuitemaker N, van Roosmalen J, Dekker G, Van Dongen P, van Geijn H, et al. (1997) Maternal mortality after cesarean section in The Netherlands. Acta Obstet Gynecol Scand 76: 332-4.

Sedgh G, Henshaw S, Singh S, Ahman E, Shah IH (2007) Induced abortion: estimated rates and trends worldwide. Lancet 370(9595):1338-45.

Shah I, Ahman E (2004) Age patterns of unsafe abortion in developing country regions. Reprod Health Matters 12(24 Suppl):9–17

Shah R, Baji S, Kalgutkar S (2005) Attitudes about medical abortion among Indian women. Int J Gynaecol Obstet 89:69-70.

Shannon C, Brothers LP, Philip NM, Winikoff B (2004) Infection after medical abortion: a review of the literature, Contraception, 70(3):183-90.

Sibley LM, Sipe TA, Brown CM, Diallo MM, McNatt K et al. (2007) Traditional birth attendant training for improving health behaviours and pregnancy outcomes. Cochrane Database System Rev 3:CD005460.

Singh A, Mavalankar DV, Bhat R, Desai A, Patel SR, SinghPV, Singha N (2009) Providing skilled birth attendants and emergency obstetric care to the poor through partnership with private sector obstetricians in Gujarat, India. Bull World Health Organ 87:960–964.

Singh S (2006) Hospital admissions resulting from unsafe abortion: estimates from 13 developing countries. Lancet 368(9550):1887-92.

Sutherland T, Bishai D. (2009) Cost-effectiveness of misoprostol and prenatal iron supplementation as maternal mortality interventions in home births in rural India. International Journal of Gynecology and Obstetrics 104 (2009) 189–193

Thaddeus S, Maine D (1994). Too far to walk: maternal mortality in context. Soc Sci Med 38(8):1091-110.

The Alan Guttmacher Institute (AGI) (2006) Preventing unsafe abortion and its consequences: Priorities for research and action. New York: Alan Guttmacher Institute. Available: http://www.guttmacher.org/pubs/2006/07/10/PreventingUnsafeAbortion.pdf. Accessed 24 August 2009.

The Alan Guttmacher Institute (AGI) (2007) Facts on induced abortion worldwide: worldwide incidence and trends. Available: http://www.guttmacher.org/pubs/fb_IAW.html. Accessed 18 August 2009.

Tietze C, Levit S (1971) Legal abortions: early medical complications. An interim report of the Joint Program for the Study of Abortion, Fam Plann Perspect, 3(4):6-14.

Tietze C, Lewit S (1972) Legal abortions: early medical complications. An interim report of the Joint Program for the Study of Abortion, J Reprod Med. 8(4):193-204.

Trussell J, Kost K (1990) Contraceptive failure in the United States: an update. Stud Fam Plann 21:51–4.

Tsu VD, Coffey PS (2009) New and underutilised technologies to reduce maternal mortality and morbidity: what progress have we made since Bellagio 2003? BJOG 116(2):247-56

Tukur J (2009) The use of magnesium sulphate for the treatment of severe pre-eclampsia and eclampsia. Ann Afr Med 8(2):76-80

UNFPA (2007) Reproductive Health Costing Tools Model. New York: UNFPA. Available : http://www.who.int/pmnch/topics/economics/costing_tools/en/index15.html. Accessed 27 July 2009.

UNFPA, PRB. Country Profiles for Population and Reproductive Health, 2005. Available online at http://www.unfpa.org/upload/lib_pub_file/524_filename_country_profiles_2005.pdf Page 126-127.

UNICEF (2004) State of the world's children. Available: http://www.unicef.org/sowc/archive/ENGLISH/The%20State%20of%20the%20World%27s%20Children%202004.pdf. Accessed 7 May 2009.

UNICEF Supply Division. Available: http://www.supply.unicef.dk/catalogue/. Accessed 27 July 2009.

UNICEF, WHO, UNFPA (2007) Maternal mortality declining in middle-income countries; women still die in pregnancy and childbirth in low-income countries. UNICEF, WHO, UNFPA Joint Press Release. Available: http://www.unicef.org/media/media_41208.html. Accessed 27 July 2009.

United Nations (UN), Department of Economic and Social Affairs, Population Division (2007) World Population Prospects: The 2006 Revision. CD-ROM Edition - Extended Dataset in Excel and ASCII formats (United Nations publication, Sales No. E.07.XIII.7).

United Nations Population Fund (UNFPA) (2003) Maternal mortality update 2002: a focus on emergency obstetric care. New York: UNFPA.

Vlassoff M, Shearer J, Walker D, Lucas H (2008) Economic impact of unsafe abortion-related morbidity and mortality: evidence and estimation challenges. Institute of Development Studies Report 59. Available: http://www.abortionresearchconsortium.org/reports/Rr59.pdf. Accessed 19 August 2009.

Vora KS, Mavalankar DV, Ramani KV, Upadhyaya M, Sharma B, Iyengar S, Gupta V, Iyengar K (2009) Maternal health situation in India: a case study. J Health Popul Nutr. 27(2):184-201.

Walraven G, Blum J, Dampha Y, Sowe M, Morison L, Winikoff B, et al. (2005) Misoprostol in the management of the third stage of labour in the home delivery setting in rural Gambia: a randomised controlled trial. BJOG 112(9):1277–83.

Weissman E, Sentumbe-Mugisa O, Mbonye AK, Lissner C (1999) Costing of safe motherhood in Uganda. In Berer M, Sundari Ravindran TK, editors. Reproductive health matters: Safe Motherhood initiatives: Critical issues. Oxford: Blackwell Science. pp 85-92.

WHO, UNICEF (1996) Revised 1990 estimates of maternal mortality: a new approach by WHO and UNICEF. Geneva: World Health Organization.Available: http://whqlibdoc.who.int/hq/1996/WHO_FRH_MSM_96.11.pdf. Accessed 1 September 2009.

WHO, UNICEF, UNFPA (2001) Maternal mortality in 1995: estimates developed by WHO, UNICEF, UNFPA. Geneva: World Health Organization. Available: http://whqlibdoc.who.int/hq/2001/WHO_RHR_01.9.pdf. Accessed 1 September 2009.

Winani S, Wood S, Coffey P, Chirwa T, Mosha F, Changalucha J (2007) Use of a clean delivery kit and factors associated with cord infection and puerperal sepsis in Mwanza, Tanzania. J Midwifery Womens Health 52(1):37-43

World Bank (2002) India's transport sector: the challenges ahead. Vol 1. Washington, D.C.: World Bank Group. Available: http://www-wds.worldbank.org/external/default/main?pagePK=64193027&piPK=64187937&theSitePK=523679&menuPK=64187510&searchMenuPK=64187283&theSitePK=523679&entityID=000094946_02070604022321&searchMenuPK=64187283&theSitePK=523679 . Accessed 23 August 2009.

World Bank. India Transport Sector http://web.worldbank.org/WBSITE/EXTERNAL/COUNTRIES/SOUTHASIAEXT/EXTSARREGTOPTRANSPORT/0,,contentMDK:20703625~menuPK:868822~pagePK:34004173~piPK:34003707~theSitePK:579598,00.html Accessed 25 August 2009.

World Bank. South Asia: data, projects, and research http://web.worldbank.org/WBSITE/EXTERNAL/COUNTRIES/SOUTHASIAEXT/0,,menuPK:158937~pagePK:158889~piPK:146815~theSitePK:223547,00.html. Accessed 25 August 2009.

World Health Organization (WHO) (2002) Estimates of DALYs by sex, cause and WHO mortality sub-region, estimates for 2001. Geneva: WHO. Available: http://www.who.int/healthinfo/global_burden_disease/estimates_regional_2001/en/index.html. Accessed 23 August 2009.

World Health Organization (WHO) (2005) World Health Report: make every mother and child count. Geneva: World Health Organization. Available http://www.who.int/whr/2005/en/ Accessed 3 September 2009.

World Health Organization (WHO) (2006) World Health Report: working together for health. Geneva: World Health Organization. Available: http://www.who.int/whr/2006/en/index.html. Accessed 23 August 2009.

World Health Organization (WHO) (2007a) World Health Statistics. Estimates by WHO Subregion. Available: http://www.who.int/healthinfo/statistics/gbdwhoregionincidence2002.xls. Accessed 7 May 2009.

World Health Organization (WHO) (2007b) Maternal mortality in 2005: estimates developed by WHO, UNICEF, UNFPA, and The World Bank. Available: http://www.who.int/whosis/mme_2005.pdf. Accessed 19 August 2009.

World Health Organization (WHO) CHOICE: CHOosing Interventions that are Cost-Effective. Available: http://www.who.int/choice/en/. (last accessed 12 July 2007)

World Health Organization (WHO). 1998. Unsafe abortion: global and regional estimates of incidence of and mortality due to unsafe abortion with a listing of available country data. Geneva, World Health Organization. (WHO/RHT/MSM/97

Yarrow C, Benoit G, Klein MC (2004) Outcomes after vacuum-assisted deliveries. Births attended by community family practitioners. Can Fam Physician 50(50):1109-14
